# An extraterrestrial trigger for the mid-Ordovician ice age: Dust from the breakup of the L-chondrite parent body

**DOI:** 10.1126/sciadv.aax4184

**Published:** 2019-09-18

**Authors:** Birger Schmitz, Kenneth A. Farley, Steven Goderis, Philipp R. Heck, Stig M. Bergström, Samuele Boschi, Philippe Claeys, Vinciane Debaille, Andrei Dronov, Matthias van Ginneken, David A.T. Harper, Faisal Iqbal, Johan Friberg, Shiyong Liao, Ellinor Martin, Matthias M. M. Meier, Bernhard Peucker-Ehrenbrink, Bastien Soens, Rainer Wieler, Fredrik Terfelt

**Affiliations:** 1 Astrogeobiology Laboratory, Department of Physics, Lund University, Lund, Sweden.; 2 Division of Geological and Planetary Sciences, California Institute of Technology, Pasadena, CA, USA.; 3 Department of Chemistry, Vrije Universiteit Brussel, Brussels, Belgium.; 4 Robert A. Pritzker Center for Meteoritics and Polar Studies, The Field Museum of Natural History, Chicago, IL, USA.; 5 Department of the Geophysical Sciences, The University of Chicago, Chicago, IL, USA.; 6 School of Earth Sciences, The Ohio State University, Columbus, OH, USA.; 7 Analytical, Environmental, and Geo-Chemistry, Vrije Universiteit Brussel, Brussels, Belgium.; 8 Laboratoire G-Time, Université Libre de Bruxelles, Brussels, Belgium.; 9 Geological Institute, Russian Academy of Sciences, Moscow, Russia.; 10 Institute of Geology and Oil and Gas Technologies, Kazan (Volga Region) Federal University, Kazan, Russia.; 11 Royal Belgian Institute of Natural Sciences, Brussels, Belgium.; 12 Department of Earth Sciences, Durham University, Durham, UK.; 13 Purple Mountain Observatory, Chinese Academy of Sciences, Nanjing, China.; 14 CAS Center for Excellence in Comparative Planetology, Hefei, China.; 15 Department of Earth Sciences, ETH Zürich, Zürich, Switzerland.; 16 Naturmuseum St. Gallen, St. Gallen, Switzerland.; 17 Department of Marine Chemistry and Geochemistry, Woods Hole Oceanographic Institution, Woods Hole, MA, USA.

## Abstract

The breakup of the L-chondrite parent body in the asteroid belt 466 million years (Ma) ago still delivers almost a third of all meteorites falling on Earth. Our new extraterrestrial chromite and ^3^He data for Ordovician sediments show that the breakup took place just at the onset of a major, eustatic sea level fall previously attributed to an Ordovician ice age. Shortly after the breakup, the flux to Earth of the most fine-grained, extraterrestrial material increased by three to four orders of magnitude. In the present stratosphere, extraterrestrial dust represents 1% of all the dust and has no climatic significance. Extraordinary amounts of dust in the entire inner solar system during >2 Ma following the L-chondrite breakup cooled Earth and triggered Ordovician icehouse conditions, sea level fall, and major faunal turnovers related to the Great Ordovician Biodiversification Event.

## Teaser

The disruption of a 150-km large asteroid filled the inner solar system with dust that cooled Earth and caused faunal turnovers.

## INTRODUCTION

During the past 500 million years (Ma), Earth has experienced three major ice ages (*1*). We live in the latest ice age that began in the Late Eocene, ~35 Ma ago, after more than 230 Ma of ice-free high-latitude continental regions. The preceding major ice age lasted from the Late Devonian to the mid-Permian, leaving behind extensive glacial deposits over ancient Gondwanaland. The oldest major Phanerozoic ice age peaked in the Late Ordovician, as indicated by glacial deposits in, e.g., North and South Africa and South America (*2*, *3*), but sea level records indicate that ice age conditions may have started already in the mid-Ordovician (*4*–*7*). Although much of Earth’s short-term climate variability is astronomically paced, as expressed by the Milankovitch cycles, the fluctuations on a 10- to 100-Ma scale between greenhouse and icehouse climates are generally explained in terms of Earth-bound causes, such as the closing or opening of seaways, uplift of mountain chains, or changes in atmospheric CO_2_ concentrations (*1*).

Here, we focus on an interval of the geological record that has been proposed to represent the onset of the Ordovician ice age and where important faunal turnovers occurred worldwide (*4*, *8*). The interval has been studied in particular detail in Baltoscandia where many well-preserved sedimentary sections are exposed. In these sections, shortly following the transition between the regional Volkhov and Kunda stages (~466 Ma ago), one of the major steps in the so-called Great Ordovician Biodiversification Event (GOBE) is registered (*4*). Similar biodiversity changes are seen in coeval Laurentian sections, indicating a global event (*8*). The GOBE concept refers to a stepwise change over ~30 Ma from a world with relatively low marine invertebrate biodiversity in the Cambrian and Early Ordovician to near-modern levels at the end of the Ordovician (*9*). The literature discusses two seemingly opposing explanations for the faunal and climatic changes in the earliest Kundan (*4*, *10*). Schmitz *et al.* (*10*) showed that the changes appear to coincide with the breakup of the L-chondrite parent body (LCPB; diameter, ~150 km) in the asteroid belt, the largest documented breakup during the past 3 billion years. Besides the abundant L-chondritic meteorites still falling on Earth from this event, common fossil meteorites (1 to 20 cm large) in mid-Ordovician sediments attest to the breakup (*11*). Schmitz *et al.* (*10*) argued that recurrent asteroid impacts on Earth after the LCPB breakup may have spurred increases in biodiversity. This is consistent with the “intermediate disturbance hypothesis” that explains biodiversity increases in recent rain forests under mild stress (*12*). This proposed GOBE-LCPB relation has been challenged on the basis of oxygen-isotope temperature records, interpreted to indicate that the Ordovician biodiversity expansion, including the mid-Ordovician biota turnover, instead relates to a gradual cooling of Earth, culminating with the icehouse conditions in the Late Ordovician (*4*, *13*). Over the past decade, further evidence has accumulated in support of an increase in asteroid impacts during the extended period when the main phase of the GOBE took place (*14*). The craters from these impacts, however, are typically small (diameter, <10 km), and no direct links between these craters and faunal turnovers have been found. The debate about a possible causal connection between the LCPB breakup and GOBE has suffered from a lack of data concerning the precise, high-resolution timing of the breakup in relation to terrestrial biotic and climatic events (*10*). Our previous less detailed chrome-spinel data placed the breakup at a stratigraphic level somewhere in the lower part of the *Lenodus variabilis* Conodont Zone, corresponding to an age of ~466 Ma ago according to the 2012 Geologic Time Scale (*15*). This age agrees with ~470-Ma Ar-Ar isotopic ages for shocked, recently fallen L chondrites [e.g., (*16*)].

**Fig. 1:**
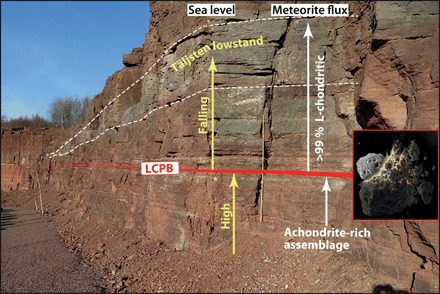
The mid-Ordovician Hällekis section in southern Sweden. The red line represents the stratigraphic level (at −1 m in this study) that corresponds to the time of the breakup of the LCPB in the asteroid belt. At this level, there is a change in the strata in abundance and types of extraterrestrial chrome-spinel grains. A low-abundance, mixed micrometeorite assemblage is replaced by a high-abundance assemblage completely dominated by L-chondritic grains. At the same level, the grain size of bioclastic limestone fragments begins to increase, indicating onset of a gradual sea level fall that culminates with the conspicuous Täljsten lowstand deposit traceable over most of Baltoscandia and likely also globally. Asteroid breakup artwork by Don Davis. (Photo credit: Birger Schmitz, Lund University)

The abundant fossil meteorites in mid-Ordovician limestone are associated with the conspicuous Täljsten interval that represents a unique episode of eustatic sea level lowstand (Fig. 1 and fig. S3) (*11*, *17*–*19*). The Täljsten has been interpreted as a lowstand systems tract of the Kunda depositional cycle (*18*, *19**)* and is traceable over most of Baltoscandia but also in Laurentia, Siberia, Gondwana, and the Yangtze platform (fig. S3). To resolve whether the LCPB breakup directly affected Earth’s climate and biota, we here use new high-resolution, multiparameter data (chrome spinel and He and Os isotopes) to locate the precise level in the sedimentary strata that corresponds to the LCPB event. We compare these data with previous noble gas data for chromite grains from large fossil meteorites (*20*, *21*) that can be used for an independent assessment of the timing of the LCPB breakup. A multiparameter approach is required because of the potentially different transport mechanisms and routes to Earth for different size fractions of asteroid breakup products, as well as uncertainties in sedimentation rates. As will be discussed, it is not simple to relate the first signal of L-chondritic material in a stratigraphic column to the precise time of the breakup of the LCPB in the asteroid belt.

### A multiparameter approach

**Fig. 2:**
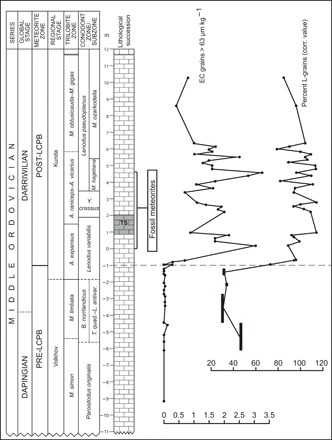
Stratigraphic scheme for the composite Hällekis-Thorsberg section and distribution of equilibrated ordinary chondritic chromite (EC) grains through the section. The stratigraphic interval is marked over which >130 fossil meteorites have been found in the Thorsberg Quarry. In 18 samples studied representing 791 kg of rock spanning the interval from −9 to −1 m relative to the base of the Arkeologen bed, we found only 15 EC grains >63 μm, i.e., 2 grains per 100 kg, which is about the same number of grains that we find per kilogram in samples in the overlying ca. 7 m of section. At −1 m, we also see a change in the abundance ratios of H, L, and LL chondritic grains from an evenly mixed assemblage to one completely dominated by L-chondritic grains. The results in the figure build on a total of 1320 kg of limestone dissolved in acids and searched for chrome spinels (see also fig. S1). TS, Täljsten lowstand deposit.

**Fig. 3:**
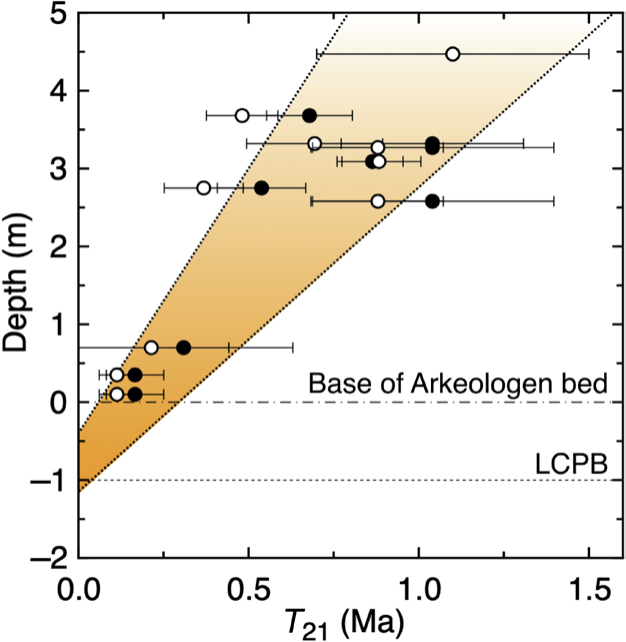
The ^21^Ne CRE ages (*T*_21_) of fossil L chondrites from the Thorsberg Quarry inversely correlate with sediment ages. Solid symbols are ages with cosmogenic ^21^Ne production rates from Heck *et al.* (*20*), and open symbols are ages with production rates determined by Heck *et al.* (*21*) (see Supplementary Text). The interval between linear regressions on the lower and upper limits, respectively, of the two data sets is shaded orange and sets the LCPB breakup at *T*_21_ = 0 Ma between −0.4 and –1.2 m relative to the base of the Arkeologen bed.

We investigated marine limestone exposed in the composite Hällekis-Thorsberg section at Kinnekulle in southern Sweden and in the Lynna River section near St. Petersburg in Russia. These are two “classical” sections studied in detail from many paleontological and sedimentological aspects [see (*4*, *11*)]. The abandoned Hällekis Quarry encompasses also the interval of limestone that has yielded >130 fossil meteorites in the active Thorsberg Quarry 4 km to the southeast (Fig. 1) (*11*, *22*). The meteorites are all (except one) L chondrites, and they have been found over the entire 5-m stratigraphic interval quarried at Thorsberg, starting at the base of the bed informally named Arkeologen by quarry workers (Fig. 2). Measurements of ^21^Ne in chromite grains from meteorites at different levels in the quarry have yielded consecutively longer cosmic ray exposure (CRE) ages, from ca. 0.1 to 1.2 Ma, with increasing stratigraphic height (*20*, *21*). This progression is best explained by all the meteorites originating from a single breakup event and reaching Earth after different exposure times to cosmic rays in space. The age succession is consistent with generally accepted average sedimentation rates for the strata (2 to 4 mm ka^−1^) [e.g., (*23*)]. The ^21^Ne data place the LCPB breakup at a stratigraphic level between ~0.4 and 1.2 m below the base of the Arkeologen bed (Fig. 3; see Supplementary Text regarding the uncertainties of this dating approach).

Shortly after the LCPB breakup, the flux of L-chondritic material to Earth may have increased gradually, a change that would be difficult to determine with confidence because a constant meteorite flux during a period when sedimentation rates decrease would yield the same grain abundance signal in the limestone as a gradually increasing flux. We can circumvent this problem by studying the change in the ratios between grains from L chondrites and other meteorite groups. Existing chrome-spinel data show that the meteorite flux to Earth before and after the LCPB breakup was very different (*24*). Studies of sediments that formed about 1 Ma before the breakup show that different types of, today very rare, achondritic micrometeorites made up ~15 to 34% of the flux. In the same beds, the three different groups of ordinary chondrites, H, L, and LL, make up about a third each of the ordinary chondritic micrometeorites. After the LCPB breakup, the total flux of extraterrestrial chromite grains (>63 μm) increased by two to three orders of magnitude, and >99% of the chromites found are L chondritic (Figs. 1 and 2).

We know that Ordovician sediment-dispersed chromite grains originate from micrometeorites because of their high concentrations of solar wind–implanted Ne and He (*21*, *25*). Solar wind ions only penetrate a few nanometers; hence, our chromite grains must, at some time, have shared surface with the enclosing silicate micrometeoroid. Large micrometeoroids (>0.1 mm) containing >32-μm chromite grains would normally require on the order of 0.3 to 2 Ma to reach Earth through Poynting-Robertson light drag from a breakup in the inner asteroid belt (table S5) (*21*). Their first appearance in the strata may thus not reflect the precise time of the LCPB breakup. The most fine-grained, micrometer-sized dust from a breakup will have much shorter transfer times, in the order of 10 thousand years (ka). The ^3^He content in sediments has been shown to be a reliable and robust indicator of this fine-grained dust (*26*). Thus, we have established a detailed extraterrestrial ^3^He profile across the extended stratigraphic interval in which the first arrival of dust from the LCPB likely is registered. In another approach, we have performed refined, high-resolution Os isotope analyses over the same strata. Previously, ^187^Os/^188^Os isotope ratios in the Hällekis section were shown to drop markedly after the LCPB breakup, reflecting abundant extraterrestrial matter in the sediment (*10*). Here, we try to find the precise (centimeter resolution) stratigraphic level where this change starts.

It is not known in what state the abundant, relict L-chondritic chromite grains reached the Ordovician seafloor. In an attempt to clarify this, we have here separated 2792 melted and 190 scoriaceous and unmelted micrometeorites in the size range of 80 to 2000 μm from recent micrometeorite-rich deposits in Antarctica. All the micrometeorites were dissolved in hydrofluoric (HF) acid, and residual chrome-spinel grains were recovered and analyzed.

## RESULTS

**Fig. 4:**
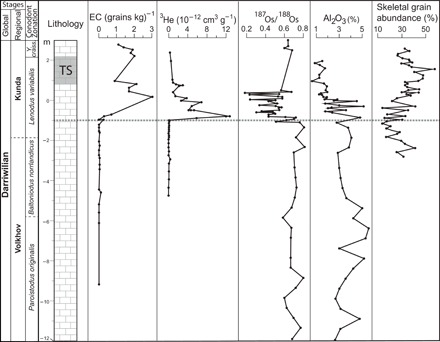
The lower part of the Hällekis section with plots of bulk-rock concentrations of equilibrated ordinary chondritic chromite (EC) grains, ^3^He and Al_2_O_3_, and ^187^Os/^188^Os ratios. In the far-right column, skeletal grain abundance according to (*41*, *42*) is shown. The chromite and He and Os isotopes indicate a sudden increase in extraterrestrial material in the sediment at −1 m, whereas the Al_2_O_3_ and skeletal grain abundances illustrate the change to a more clean and coarse-grained limestone that can be used for production of industrial limestone slabs. The coarsening of the sediment reflects stronger hydrodynamic forcing with shallowing, leading to winnowing of the fine fraction.

The results of all the parameters studied across the Hällekis section show that the first signal of the LCPB breakup occurs 1 m below the base of the Arkeologen bed, in the *L. variabilis* Conodont Zone (Figs. 2 and 4). At this level, we see the gradual onset of a marked rise in the number of extraterrestrial chromite grains. There is also a sharp change at −1 m in the ratios between different meteorite groups toward complete dominance of L-chondritic grains (Fig. 2). Extremely high numbers of extraterrestrial chromite grains continue upward through the section for at least 8 m, corresponding to 2 to 4 Ma. Extraterrestrial ^3^He shows a marked and sudden rise at the −1-m level, which indicates that the first fine-grained dust arrived on Earth at the same (±50 ka) time as the first abundant LCPB-related micrometeorites (Fig. 4). Osmium isotopes also show a major shift at this level, indicative of increased delivery of extraterrestrial matter to the seafloor. Both the He and Os isotopes indicate elevated abundances of extraterrestrial matter for some meters upward through the section, in concordance with the chromite data. It is clear from our detailed studies that the sudden increase in extraterrestrial material at −1 m reflects the arrival of the first LCPB dust on Earth and cannot be explained by a stratigraphic hiatus (fig. S2).

**Fig. 5:**
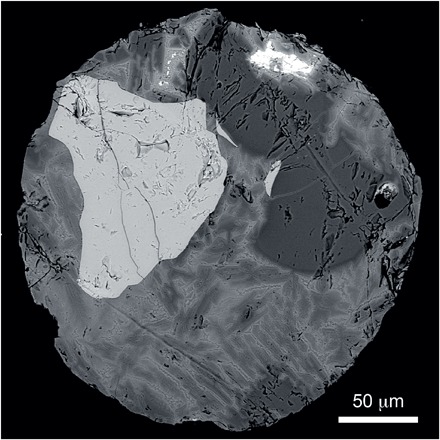
A chromite grain in an Antarctic micrometeorite. Back-scattered electron image of a porphyritic olivine spherule with large (>63 μm) chromite relict grain (light gray). This grain is not included in the present study but is shown here to illustrate the distribution of relict grains in cosmic spherules, often found near the particle edge. This relict chromite grain is angular, with limited indication of melting during atmospheric passage.

In the almost 3000 Antarctic micrometeorites dissolved in HF, we only found a single ordinary chondritic chromite grain of >32 μm, an angular grain of ~60 μm in diameter, and two chrome-spinel grains ~20 μm large from other meteorite types (see data file S4 for details). The grains were found in the melted micrometeorites, which is consistent with chromite and other chrome-spinel types being among the minerals in meteorites with the highest melting temperatures. Chromite apparently can survive atmospheric passage when most other minerals melt or recrystallize (Fig. 5). From other studies of recent micrometeorite assemblages, we can deduce that about one-fifth of the micrometeorites that we dissolved were ordinary chondrites (*27*, *28*). This means that about 1 in 600 ordinary chondritic micrometeorites contains a chromite grain of >32 μm. Thus, the up to ~50 chromite grains >32 μm kg^−1^ in post–LCPB breakup limestone represent the residue on the order of 30,000 micrometeorites (>0.1 mm) per kilogram of sediment.

## DISCUSSION

On the basis of our new results, we place the breakup of the LCPB at a time corresponding to the −1-m level in the section, consistent with previous ^21^Ne CRE ages for the fossil meteorites (Fig. 3). As Poynting-Robertson transfer times to Earth increase with increasing particle size, ^3^He hosted primarily in the fine fraction would be expected to increase in the strata ~1 to 2 m below the level where abundant large chromite grains first appear. The simultaneous appearance on Earth of fine dust and coarser micrometeorites can be reconciled in light of the low ^21^Ne CRE ages for the fossil meteorites. Recent ordinary chondrites typically have CRE ages in the range of 3 to 60 Ma, compared to ~0.1 to 1.2 Ma for the Ordovician fossil L chondrites (*20*, *21*). The latter short durations have been explained by positioning the LCPB breakup close to an orbital resonance that would have sent dust and meteoroids to Earth at much shorter time scales than normal Poynting-Robertson drag would alone (*29*). Considering the very short (~100 to 200 ka) ^21^Ne CRE ages of the oldest recovered L-chondritic fossil meteorites (from the Arkeologen bed) (Fig. 3), we argue that also millimeter-sized micrometeoroids had very short travel times. This would explain why the fine-grained and coarser dust arrived to Earth at about the same time. We note that our ^21^Ne data have been used by others to place the LCPB breakup at 0.2 m below the base of the Arkeologen bed (*30*), an interpretation that we dispute because it is impossible that the breakup took place 200 to 400 ka after the first arrival of extraordinary amounts of L-chondritic micrometeorites on Earth.

On the basis of comparisons of the content of extraterrestrial chromite in the post-LCPB breakup sediments with similar condensed sediments from the Late Silurian, Middle and Late Devonian, Early and Late Cretaceous, and the Early Paleocene, we know that the chromite flux in the >32-μm fraction during >2 Ma after the disruption of the LCPB was two to three orders of magnitude higher than the background flux through the rest (or most) of the Phanerozoic [e.g., (*31*, *32*)]. There is a size dependency in the flux of extraterrestrial material to Earth after a breakup event. In agreement with modeling scenarios, our empirical data indicate that the post–LCPB breakup increase in the flux of 100-m-sized asteroids, decimeter-sized meteoroids, and millimeter-sized micrometeoroids, was one, two, and two to three orders of magnitudes, respectively (*11*, *14*, *31*, *32*). The increase in the flux of the most fine-grained, micrometer-sized material was certainly even higher, but our Ordovician ^3^He data cannot resolve the magnitude because gas loss from the 466-Ma-old sediment has occurred (*33*). However, on the basis of the ^3^He versus chromite relation in ^3^He anomalies in much younger sediments (~36 and ~91 Ma old) (*32*, *34*), we are confident about a three to four orders of magnitude increase in the fine-fraction flux after the LCPB breakup.

We argue here that ice age conditions in the mid-Ordovician, postulated by other groups [e.g., (*4*–*7*)], were triggered or intensified by the LCPB breakup. We hypothesize that the origin of the prominent Täljsten lowstand deposit (Fig. 1) that has puzzled regional geologists for more than a century (*35*) can be explained by a eustatic sea level fall related to global cooling triggered by the dust from the LCPB breakup. In Earth’s atmosphere today, extraterrestrial dust represents about 1% of the total stratospheric dust and has no direct climatic significance (*36*, *37*). However, cooling is to be expected if the amount of extraterrestrial dust in the atmosphere for several 100 ka or longer increases by more than three orders of magnitude. Following the LCPB breakup, not only Earth’s atmosphere but also much of interplanetary space in the inner solar system became dusty, further shading Earth from sunlight [see e.g., *38*, *39*]. Dust from the LCPB breakup may also have fertilized large areas of the ocean, which could have led to drawdown of CO_2_ from the atmosphere (*40*). The mechanisms leading to cooling are complex and are influenced by the character of the dust, e.g., size, albedo, mineralogy, and chemical composition. Further research is needed to explore the full parameter space that connects enhanced dust delivery to climate cooling.

Previous studies of the Täljsten sea level fall have placed its onset, in both the Hällekis and the Lynna sections, at the precise level where our new data show arrival of the first LCPB dust (Fig. 4 and fig. S5) (*4*, *41*, *42*). In the Hällekis section, the bioclastic particles (from invertebrate skeletal material) making up the limestone are generally coarser in the Täljsten interval than through the rest of the section. The coarser grain size indicates stronger (i.e., with shallower water) hydrodynamic forcing that preferentially removes finer particles from the seafloor sediments. This change to coarser grain size starts at the −1-m level (Fig. 4) (*41*, *42*). The Al_2_O_3_ profile, which reflects the amount of fine-grained clay in the limestone, shows a drop at the −1-m level, illustrating the onset of the change toward more coarse-grained and cleaner limestone (Fig. 4). Quarry workers have known for centuries that, in the ~54-m section of Ordovician limestone exposed at Hällekis, only a ~3-m interval of the strata, with the Täljsten at its core, is sufficiently clean to be used for the production of commercial limestone slabs (*35*). We note that, if the LCPB breakup had not led to a change in Ordovician seafloor hydrodynamics, then there would have been no rocks to quarry for limestone slabs and no fossil LCPB meteorites would have been found. Another argument is based on the detailed studies by Rasmussen *et al.* (*4*) of brachiopod faunas in the Lynna River valley section and our chromite reconstructions for the same section (fig. S5). The first LCPB dust in the strata occurs close to the stratigraphic level where a shallow-water fauna of brachiopods replaces deeper-water faunas. This faunal change then culminates with the low-stand deposits ca. 2 m higher in the section, which are coeval with the Täljsten in Sweden. The shallowing recorded at Lynna is attributed by Rasmussen *et al.* (*4*) to ice buildup on continents at the onset of the Ordovician ice age. In another mid-Ordovician section that we previously studied at Puxi River in China, we observe a similar trend. There, unusual biogenic micromounds, which may reflect shallowing, formed at the seafloor shortly after the first abundant chromite grains from the LCPB event arrived on Earth (*11*).

Establishing sea level curves from sedimentary sections is a difficult task involving also subjective considerations. Therefore, various sea level curves for Darriwilian deposits in Baltoscandia are sometimes contradictory. In particular, the magnitude of a sea level change is often very difficult to quantify. However, the prominent sea level lowstand associated with the Täljsten is a very obvious feature [e.g., *17*–*19*, *41*–*44*]. The 1.4-m thick, gray Täljsten in the middle of a ca. 27-m-thick section of otherwise red Ordovician limestone starts ca. 0.8 m above the base of the Arkeologen bed. Besides the coarser grain size, the Täljsten shows many other features indicative of marked shallowing, such as anomalous shallow-water fossil faunas of gastropods, echinoderms, and ostracods. In some beds, centimeter-sized echinoderm fossils build the rock (fig. S4). Oncoids and stromatolites, both clear shallow-water indicators, have also been reported (*44*).

The Täljsten lowstand occurred simultaneously over Baltoscandia (e.g., Fig. 1 and fig. S3) consistent with a eustatic origin (*18*, *19*). The setting on the interior of a large, stable craton also supports a eustatic signal (*4*). Detailed studies of the mid-Ordovician sea level evolution on the Siberian craton, which at the time represented a separate paleocontinent from Baltica, place the most prominent sea level lowstand at the base of the Mukteian-Vikhorevian regional stage, which correlates with the base of the Baltic Kunda stage (*45*). A coeval major sea level fall has been proposed for other paleocontinents, including Gondwana and Laurentia [(*6*); see fig. S3], although the biostratigraphic correlations have uncertainties related to the provincialism in mid-Ordovician invertebrate faunas. Future studies using chrome-spinel grains as a precise global correlation tool will refine our understanding of the age relations between sediment sections on different paleocontinents.

Our revised “astrogeobiological” explanation for the conspicuous faunal diversifications observed in the mid-Ordovician involves breakup of an ~150-km large asteroid in the asteroid belt (*16*, *46*), which flooded the inner solar system with dust. The sudden global change from an equable greenhouse situation to a climatically more heterogeneous icehouse world spurred the GOBE. The cooling increased latitudinal temperature gradients, requiring adaptions by the biota acclimatized to a warm climate. Faunal changes are therefore expected to be more pronounced at mid-to-high latitudes, such as in mid-Ordovician Baltoscandia, than in low-latitude regions. The prominent Täljsten sea level fall may reflect the sudden onset of a continuous, mid-to-late Ordovician ice age, or it may only represent a shorter, intensified icehouse period superimposed on a general cooling trend that culminated in the Late Ordovician (*13*). The dust from the LCPB could have been the tipping factor triggering an icehouse world. Some oxygen isotope data indicate very warm oceans in the Early Ordovician, and authors have been puzzled by the apparently sudden evolution of icehouse conditions in the mid-Ordovician (*7*, *47*). The enigma could be an artifact of the many difficulties in interpreting Early Paleozoic oxygen isotope data (*48*), but an alternative explanation links sudden cooling to dust from the LCPB.

In an effort to mitigate ongoing global warming, it has been suggested to capture a large near-Earth asteroid and position it at the first Lagrange point as a source of dust that could help to reduce solar insolation on Earth (*39*). Gravitationally “anchoring” such a dust cloud at this point would reduce dust particle dispersion and create a prolonged cooling effect. Such an anchored cloud can lead to insolation reductions to Earth three times larger than the reduction required to offset a CO_2_-induced increase of 2°C in mean global temperature. The >2 Ma of strongly enhanced dust flux to Earth after the LCPB breakup must reflect a complex series of events, including secondary collisions of asteroid fragments from the LCPB, greatly enhanced numbers of near-Earth asteroids, and, speculatively, even dust clouds anchored at unusual gravitational locations. In any case, studies of the extraterrestrial fraction of mid-Ordovician sediment provide new empirical knowledge that is relevant in the context of present-day climate mitigation.

## MATERIALS AND METHODS

### Separation of chrome-spinel and chemical analyses

The present study builds on the results from previous chrome-spinel studies of the Hällekis-Thorsberg and Lynna River sections, as well as new samples prepared for this study (see data files S1 and S5) for further details. A total of 1320 and 188 kg of limestone at Hällekis-Thorsberg and Lynna River, respectively, have been dissolved in acids and searched for chrome-spinel. The method description here discusses only the treatment of the new samples. The sample preparations were conducted at the Astrogeobiology Laboratory at Lund University (www.astrogeobiology.org) especially built for the separation and extraction of extraterrestrial minerals from sediments. The laboratory has a capacity to dissolve 5 to 10 metric tons of limestone in hydrochloric (HCl) and HF acid per year. The samples were thoroughly cleaned with a high-pressure washer to remove the weathered material and detritus and then placed in 500-liter plastic barrels with 6 M HCl acid. After ca. 48 hours, the insoluble residue was neutralized with sodium hydroxide and sieved through a 32-μm mesh. The residue was once again treated with HCl for ca. 24 hours and then neutralized with sodium hydroxide and sieved. The resulting residual >32-μm fraction was treated with 11 M HF acid for 48 hours to dissolve the siliciclastic material. After neutralization of the HF acid by means of repeated water decanting, the remaining mineral residue was treated with 18 M sulfuric acid for 12 hours to dissolve hydroxide minerals. After neutralization with sodium hydroxide and sieving through a 32-μm mesh, most of the samples were further treated with undiluted high-density LST (lithium heteropolytungstate) liquid to remove the organic material. The heavy residues in the two size fractions 32 to 63 μm and 63 to 355 μm were recovered and searched beneath a stereo microscope for opaque and transparent spinel grains that were picked with a fine brush. The grains were mounted in epoxy resin, together with analytical standard UWCr-3 *(**49*), and polished with 1-μm diamond paste. The polished grains were then coated with carbon and quantitatively analyzed at Lund University for chemical composition with a calibrated Oxford INCA X-Sight energy-dispersive spectrometer with a Si detector, mounted on a Hitachi scanning electron microscope (SEM-EDS). Cobalt was used as a standard to monitor instrumental drift. An acceleration voltage of 15 kV, a sample current of ~1 nA, and a counting live time of 80 s was used. Precision of the analyses was typically better than 1 to 4%. Typically, three spots were analyzed on each grain, and the average result is used here. Analysis spots were selected away from grain fractures or rims with signs of diagenetic alteration.

### Helium isotope analyses

Helium isotope concentrations were measured using standard practices at Caltech (*50*). Briefly, each sample of sedimentary rock was dried at 90°C for several hours and crushed in a jaw crusher to ~250-μm chips. Several grams of crushate were then leached of carbonate in 10% acetic acid until no CO_2_ evolution was observed even after agitation, and the residue was centrifuged and transferred into a tin sample cup. After drying at 90°C, the amount of residual mass was determined. This provided the operationally defined noncarbonate fraction estimate. Multiple samples were transferred simultaneously into the vacuum system and pumped for at least several hours. Helium was extracted sequentially from each sample by heating to 1200°C in a double-walled resistance furnace for 30 min. Evolved helium was purified and cryo-focused and then analyzed using a simultaneous detection mode on a GV-SFT mass spectrometer. Typical blank levels were 0.75 × 10^−15^ cm^3^ at standard temperature and pressure (STP) ^3^He and 0.2 × 10^−9^ cm^3^ STP ^4^He. With the exception of a few samples, these blank levels were less than a few percent on ^3^He and ≪1% of ^4^He. For the least ^3^He-rich samples, the blank makes up as much as 10% of the total ^3^He measured. Estimated uncertainties were derived from the uncertainty in the blank correction and the reproducibility of standards of size comparable to the sample.

### Osmium analyses

Here, we used the results of previous osmium (Os) analyses across the Hällekis section, described in (*10*), as well as new analyses of nine samples collected at a high resolution between 0.54 and 1.1 m below the base of the Arkeologen bed. For the Os analyses, whole-rock samples were ground into a fine powder with an agate mortar. Between 3 and 10 g of powdered sample were mixed with an isotopically enriched spike (containing ^99^Ru, ^105^Pd, ^190^Os, ^191^Ir, and ^198^Pt), dried at room temperature overnight, and then mixed with a flux consisting of borax, nickel, and sulfur powder. The typical sample/flux weight ratio used was 2. After fusing the mixture for 90 min at 1000°C in a glazed ceramic crucible, the melt was allowed to cool and the NiS bead was separated from the glass. The bead was then dissolved in HCl acid (6.2 M HCl), and the residue was filtered through 0.45-μm cellulose filter paper. Insoluble platinum group element–containing particles on the filter paper were dissolved together with the filter paper in 1 ml of concentrated nitric acid (HNO_3_) in a tightly closed Teflon vial at ~100°C for about 60 min immediately before Os isotope analysis. After dissolution of the filter paper and oxidation of Os, the Teflon vial was chilled in ice water to minimize the escape of volatile OsO_4_. Osmium was then extracted from this vial with the sparging method (*51*) that relies on purging dissolved OsO_4_ with inert Ar carrier gas and by transferring directly the gas mixture into the torch of a multicollector inductively coupled plasma mass spectrometer (ThermoFinnigan Neptune) and analyzing Os isotopes and potential interferences with a multidynamic data acquisition procedure using three continuous dynode electron multipliers. The details of the method are described in (*52*). The accuracy and precision of the analytical data have been evaluated in detail by (*52*) and (*53*) using various international reference materials and community standards.

### Bulk-rock Al_2_O_3_ analyses

The Al_2_O_3_ results in Fig. 4 are for ground whole-rock samples that were fused with LiBO_2_ and dissolved in HNO_3_. Analyses were made with an inductively coupled plasma atomic emission spectrometer. The relative reproducibility (2σ) of the analyses is better than 5% [see further details in (*54*) and references therein].

### Division of chrome-spinel grains

The interpretation of the origin of recovered chrome-spinel grains follows practices developed in a number of studies over the past decade [see, e.g., (*11*, *24*, *31*, *32*, *49*, *55*, *56*)]. Below is given the full description of how grains are divided into different groups, but in the present study, with focus on the consequences of dust from the LCPB breakup on Earth’s climate and biota, only the results for the equilibrated ordinary chondritic chromite (EC) grains are relevant. We deal with the abundance variations of EC grains through the section and how the ratios between H, L, and LL grains among the EC grains vary through the section. Other aspects of the results, such as the high numbers of achondritic grains in pre-LCPB sediments (*24*), or the variations in terrestrial chrome-spinel grains through the section, will be dealt with elsewhere, although all data are presented in data file S1.

#### Division into main groups

1) EC: Grains from equilibrated ordinary chondrites (petrological types 4 to 6) with oxide weight percentages within the ranges of ~53.0 to 62.0 Cr_2_O_3_, ~23.0 to 32.0 FeO, ~4.5 to 8.5 Al_2_O_3_, ~1.3 to 4.5 MgO, ~0.55 to 0.95 V_2_O_3_, and ~1.40 to 4.50 TiO_2_ [for more detailed discussions, see (*11*), p. 127]. The FeO values of EC grains can sometimes be lower than 23% because of replacement by MnO and/or ZnO [see (*57*)].

2) OtC-V: Other chrome-spinel, i.e., grains that do not have the typical equilibrated ordinary chondritic composition but contain ≥0.45 weight % (wt %) V_2_O_3_ and a Cr_2_O_3_/FeO ratio of ≥1.45, indicating a likely meteoritic origin.

3) OtC: Other chrome-spinel grains but with V_2_O_3_ < 0.45 wt % or V_2_O_3_ ≥ 0.45 wt % together with a Cr_2_O_3_/FeO ratio of <1.45. Most or all the OtC grains are likely of terrestrial origin.

The type of grains here referred to as OtC grains have, in our previous studies, been referred to as OC grains, but we have changed the acronym to avoid confusion with OC being used for “ordinary chondrites” in other research.

#### Division of EC grains in H, L, and LL groups

The EC grains can be further divided into the three groups H, L, and LL based on their oxygen isotope and TiO_2_ contents, but here, we used only the latter parameter [see (*24*, *31*, *32*, *55*, *56*)]. The three groups of ordinary chondrites, H, L, and LL, have different average values of Δ^17^O (0.73, 1.07, and 1.26‰) and TiO_2_ (2.2, 2.7, and 3.4 wt %, respectively) (*56*, *58*, *59*). Around these averages, the Δ^17^O and TiO_2_ values are spread following a Gaussian distribution, but the distributional tails overlap (*56*). Although the distinction between the equilibrated ordinary chondritic groups can be done with oxygen-3-isotopic analysis, separating them with TiO_2_ has been proven as effective (*55*, *56*). The exact definitions of the ranges for dividing grains based on TiO_2_ can, in principle, be arbitrarily set but must be used consistently when comparing different time periods. We used the following ranges in TiO_2_ concentration: H ≤ 2.50 wt %, L = 2.51 to 3.39 wt %, and LL ≥ 3.40 wt % (*55*). The TiO_2_ content of each group follows a Gaussian distribution with about 10% overlap between the groups. This overlap is insignificant when each of the three groups has similar abundances, but when one group strongly dominates, such as the L chondrites after the LCPB, the overlap creates false high numbers of grains in the other groups. We therefore present our data in two ways: corrected for a 10% overlap between groups and uncorrected for overlap in TiO_2_ concentration ranges (Fig. 2, fig. S1, and tables S2 and S3).

### Separation of Antarctic micrometeorites

A total of 2.8 kg of micrometeorite-rich sediment from Antarctica (see fig. S6 and Supplementary Text for collection sites) was processed by washing in Milli-Q H_2_O and sieving at the Vrije Universiteit Brussel (Brussels, Belgium) to separate size fractions of <125 μm, 125 to 200 μm, 200 to 400 μm, 400 to 800 μm, 800 to 2000 μm, and >2000 μm, while the remaining half is kept for reference and other research purposes. All size fractions were subjected to magnetic separation using hand magnets. Using optical microscopy and micro x-ray fluorescence spectrometry, 2039 cosmic spherules and 190 partially melted (scoriaceous) and unmelted micrometeorites were handpicked from the four magnetic fractions between 125 and 2000 μm. On the basis of the inspection of multiple small subsamples, no significant number of residual micrometeorites remained in the nonmagnetic fractions. Surficial textural characteristics and diameters were determined for these particles using a FEI ESEM Quanta 200 environmental scanning electron microscope at the Royal Belgian Institute of Natural Sciences (Fig. 5 and figs. S7 and S8). Additional but smaller subsamples of sediment (<150 g) were processed at the Astrogeobiology Laboratory at Lund University following a protocol similar to the one described above. Here, the studied size fractions of 80 to 200 μm, 200 to 300 μm, 300 to 500 μm, and 500 to 700 μm led to the recovery of an additional 753 cosmic spherules. The total of 2982 micrometeorites was dissolved in HF acid at Lund University. All residual mineral grains were collected on filter paper and analyzed by SEM-EDS for elemental composition (data file S4).

## Supplementary Material

http://advances.sciencemag.org/cgi/content/full/5/9/eaax4184/DC1

Download PDF

Data file S1

Data file S2

Data file S3

Data file S4

Data file S5

An extraterrestrial trigger for the mid-Ordovician ice age: Dust from the breakup of the L-chondrite parent body

## SUPPLEMENTARY MATERIALS

Supplementary material for this article is available at http://advances.sciencemag.org/cgi/content/full/5/9/eaax4184/DC1 Supplementary Text Fig. S1. Distribution of equilibrated ordinary chondritic chromite (EC) grains through the Hällekis-Thorsberg section. Fig. S2. High-resolution ^187^Os/^188^Os isotope profile across a proposed discontinuity surface. Fig. S3. The gray Täljsten in the Degerhamn Quarry, southern Öland. Fig. S4. Cystoid echinoderms in the Likhall bed of the Täljsten. Fig. S5. Distribution of extraterrestrial chromite across the Lynna River section. Fig. S6. Map of Antarctic micrometeorite localities. Fig. S7. Back-scattered electron images of Antarctic micrometeorites. Fig. S8. Size distribution of Antarctic micrometeorites. Table S1. Chrome-spinel distribution through the Hällekis-Thorsberg section. Table S2. Extraterrestrial chromite division below reference level in Hällekis section. Table S3. Extraterrestrial chromite division above reference level in Thorsberg section. Table S4. Published abundances of micrometeorite types from different collections. Table S5. Poynting-Robertson transfer times (Ma) from the outer solar system to Earth. Data file S1. Hällekis-Thorsberg section—chrome-spinel chemical results. Data file S2. Helium isotope data. Data file S3. Osmium isotope data. Data file S4. Spinels in Antarctic micrometeorite. Data file S5. Lynna River section—chrome-spinel chemical results. References (*60*–*88*)
