# Supplementary material for: An extraterrestrial trigger for the mid-Ordovician ice age: Dust from the breakup of the L-chondrite parent body
Source: Sci Adv. 2019 Sep 18;5(9):eaax4184. doi: 10.1126/sciadv.aax4184 (PMC6750910; doi:10.1126/sciadv.aax4184)
Supplement: http://advances.sciencemag.org/cgi/content/full/5/9/eaax4184/DC1 [file supp_5_9_eaax4184__index.html]

Science Advances | Science AdvancesAAASSearchScience AdvancesMenu

## Supplementary Materials

**The PDF file includes:**

- Supplementary Text
- Fig. S1. Distribution of equilibrated ordinary chondritic chromite (EC) grains through the Hällekis-Thorsberg section.
- Fig. S2. High-resolution 187Os/188Os isotope profile across a proposed discontinuity surface.
- Fig. S3. The gray Täljsten in the Degerhamn Quarry, southern Öland.
- Fig. S4. Cystoid echinoderms in the Likhall bed of the Täljsten.
- Fig. S5. Distribution of extraterrestrial chromite across the Lynna River section.
- Fig. S6. Map of Antarctic micrometeorite localities.
- Fig. S7. Back-scattered electron images of Antarctic micrometeorites.
- Fig. S8. Size distribution of Antarctic micrometeorites.
- Table S1. Chrome-spinel distribution through the Hällekis-Thorsberg section.
- Table S2. Extraterrestrial chromite division below reference level in Hällekis section.
- Table S3. Extraterrestrial chromite division above reference level in Thorsberg section.
- Table S4. Published abundances of micrometeorite types from different collections.
- Table S5. Poynting-Robertson transfer times (Ma) from the outer solar system to Earth.
- References (*60*–*88*)

Download PDF

**Other Supplementary Material for this manuscript includes the following:**

- Data file S1 (.pdf format). Hällekis-Thorsberg section—chrome-spinel chemical results.
- Data file S2 (Microsoft Excel format). Helium isotope data.
- Data file S3 (Microsoft Excel format). Osmium isotope data.
- Data file S4 (Microsoft Excel format). Spinels in Antarctic micrometeorite.
- Data file S5 (.pdf format). Lynna River section—chrome-spinel chemical results.

**Files in this Data Supplement:**

- Adobe PDF - aax4184\_SM.pdf
- Adobe PDF - aax4184\_Data\_file\_S1.pdf
- Adobe PDF - aax4184\_Data\_file\_S5.pdf
