# Supplementary material for: An extraterrestrial trigger for the mid-Ordovician ice age: Dust from the breakup of the L-chondrite parent body
Source: Sci Adv. 2019 Sep 18;5(9):eaax4184. doi: 10.1126/sciadv.aax4184 (PMC6750910; doi:10.1126/sciadv.aax4184)
Supplement: Download PDF [file aax4184_SM.pdf]

Science Advances
AAAS

advances.sciencemag.org/cgi/content/full/5/9/eaax4184/DC1

Supplementary Materials for

An extraterrestrial trigger for the mid-Ordovician ice age: Dust from the breakup of the L-chondrite parent body

Birger Schmitz*, Kenneth A. Farley, Steven Goderis, Philipp R. Heck, Stig M. Bergström, Samuele Boschi, Philippe Claeys, Vinciane Debaille, Andrei Dronov, Matthias van Ginneken, David A.T. Harper, Faisal Iqbal, Johan Friberg, Shiyong Liao, Ellinor Martin, Matthias M. M. Meier, Bernhard Peucker-Ehrenbrink, Bastien Soens, Rainer Wieler, Fredrik Terfelt

*Corresponding author. Email: birger.schmitz@nuclear.lu.se

Published 18 September 2019, *Sci. Adv.* **5**, eaax4184 (2019)
DOI: 10.1126/sciadv.aax4184

The PDF file includes:

Supplementary Text
Fig. S1. Distribution of equilibrated ordinary chondritic chromite (EC) grains through the Hällekis-Thorsberg section.
Fig. S2. High-resolution $^{187}Os/^{188}Os$ isotope profile across a proposed discontinuity surface.
Fig. S3. The gray Täljsten in the Degerhamn Quarry, southern Öland.
Fig. S4. Cystoid echinoderms in the Likhall bed of the Täljsten.
Fig. S5. Distribution of extraterrestrial chromite across the Lynna River section.
Fig. S6. Map of Antarctic micrometeorite localities.
Fig. S7. Back-scattered electron images of Antarctic micrometeorites.
Fig. S8. Size distribution of Antarctic micrometeorites.
Table S1. Chrome-spinel distribution through the Hällekis-Thorsberg section.
Table S2. Extraterrestrial chromite division below reference level in Hällekis section.
Table S3. Extraterrestrial chromite division above reference level in Thorsberg section.
Table S4. Published abundances of micrometeorite types from different collections.
Table S5. Poynting-Robertson transfer times (Ma) from the outer solar system to Earth.
References (*60–88*)

Other Supplementary Material for this manuscript includes the following:

(available at advances.sciencemag.org/cgi/content/full/5/9/eaax4184/DC1)

Data file S1 (.pdf format). Hällekis-Thorsberg section—chrome-spinel chemical results.
Data file S2 (Microsoft Excel format). Helium isotope data.
Data file S3 (Microsoft Excel format). Osmium isotope data.
Data file S4 (Microsoft Excel format). Spinels in Antarctic micrometeorite.
Data file S5 (.pdf format). Lynna River section—chrome-spinel chemical results.

Supplementary Text

Additional description of results

In the eighteen samples studied from the Hällekis section, and representing 791 kg of rock spanning the interval from 9 m to 1.05 m below the base of the Arkeologen bed, we found only 15 EC grains >63 µm, i.e. 2 grains per 100 kg, which is about the same number of grains that we find per kilogram in samples in the overlying ca. 7 meters of section (Fig. 2 and fig. S1). In the 32-63 µm size fraction we find about 20 to 60 grains per 100 kg in the interval below -1.05 m, whereas in the interval above we estimate there is on the order of 2000 to 7000 such grains per 100 kg sediment (see also, *31;* data S5). In the interval from 1.05 to 0.5 m below the base of the Arkeologen bed there appears to be a gradual increase upward in the number of EC grains in the sediment. Because there are also changes in the character of the sediments over this interval (see below) this change could have reflected decreasing sedimentation rates rather than the arrival of the first dust from the LCPB. The synchronous change in ratios between H, L, and LL chondrites, however, can only be explained in terms of the arrival of abundant L-chondritic dust (Fig. 2 and fig. S1; tables S2 and S3).

The $^{187}Os/^{188}Os$ values through the lower 11 m of section lie stable at terrestrial values of ca. 0.8, then at one meter below the Arkeologen bed there is a dramatic change to values of 0.3-05, values which persist upward over the 3 meters of section studied for Os isotope ratios (Fig. 4). In this interval the values scatter more than in the interval with terrestrial values below, which likely reflects that the extraterrestrial micron-sized particles carrying Os can be quite heterogeneously distributed in a sediment. This nugget effect has also been observed in other Os-isotopic studies of sedimentary strata (*60*).

Is there a hiatus at ca. -1 meter?

The sudden increase in extraterrestrial matter registered at the -1 m level raised the possibility that this is an artefact related to a stratigraphic unconformity. There are no sudden changes in the fossil ostracode, trilobite or conodont faunas that could indicate any significant stratigraphic break at or close to this level (*61-63*). However, there is an unusual sedimentological feature at -0.82 m that has been described as a "planar discontinuity surface" by Lindskog et al. [(*41*) at -1.45 m in their profile, with another zero level than in our study]. We therefore collected samples

at a high (cm-) resolution for analyses of Os isotopes and chrome-spinel grains across this surface in order to assess where exactly the first LCPB dust occurs. The bulk-rock $^{187}Os/^{188}Os$ values clearly show their first drop towards lower values about 20 cm below the "planar surface" (fig. S2). Because of the generally heterogeneous distribution of Os-carrying extraterrestrial particles in sediments (see previous section) there is scatter in the data, but there is a trend that can best be described as a gradual decline in $^{187}Os/^{188}Os$, starting at about -1 m. In addition two 4.3 and 5.5 kg large limestone samples were collected each spanning an interval immediately above (-0.54 to -0.82 m) and below (-0.82 to -1.10 m), respectively, the "planar surface". Particular care was taken to avoid admixture of any material from above the "planar surface" into the sample from below the surface. The upper sample contained 9.3 EC grains >32 μm per kg, compared to 2.2 grains per kg in the lower sample. The latter value is a factor 4 to 10 higher than typical background values in limestone in the section below, indicating that the increase in the LCPB starts somewhere in the -1.10 to -0.82 m interval, and not above the "planar surface". Another larger sample (62 kg), spanning from -0.82 to -1.05 m clearly contained a factor of 10 more EC grains (>63 μm) than background limestone below, but also a factor of 10 fewer such grains than limestone in the upward continuation of the profile. In this sample we see the first change toward dominance of L- over H- and LL-chondritic grains. The fraction of L-chondritic grains, 74%, is intermediate between the average values of 33% and 99% in the limestone samples below and above this sample, respectively (tables S2 and S3). It is clear from our data that the first signature of the LCPB occurs below the "planar surface".

When sampling for analyses of extraterrestrial ^{3}He, we did not focus on the interval where we had found the rise in L-chondritic chromite grains at ca. -1 m. We assumed the fine fraction of extraterrestrial matter would have arrived earlier, and executed great care in establishing data with precise depth assignments over a slightly older interval (Fig. 4). We were more relaxed in the intervals that we assumed represented post- and pre-LCPB background conditions. To our surprise the ^{3}He data showed a dramatic rise between samples marked in the field as "-1.05 m" and "-0.9 m". Although the distance between the two samples is correct, there is an uncertainty regarding where exactly (on a cm resolution) the "-0.9 m" sample in the ^{3}He profile was collected relative to the planar surface at the -0.82 m level. Sedimentary beds are irregular features and when measuring a section one can get slightly different depths relative to a

reference level on a cm-scale depending on the specific outcrop where the measurements are performed. There is little doubt, however, that the rise in ^{3}He occurs at or very close to 1 m below the base of the Arkeologen bed.

Both the Os-isotope and the chrome-spinel results indicate that the "planar surface" does not represent a significant discontinuity or omission surface. The surface occurs in the middle of a ~14 cm thick homogeneous, red limestone bed with little clay or marl. When exposed, the surface is perfectly flat, which is very different from the hard grounds that appear throughout the section. These omission surfaces are generally uneven with cm-deep sinks and ridges and often phosphatized. We tentatively interpret the "planar surface" at -0.82 m as a shear face, but the origin is not well understood. Lindskog et al. (*41*) show in thin sections that even at a microscopic scale the surface appears exceptionally smooth. The authors write that this planar surface marks the beginning of shallowing, "with overall lowstand conditions persisting through the Täljsten and its immediately superjacent strata".

<u>The Täljsten interval</u>

Already Holm (*35*) in 1901 notes that the Täljsten interval contains the "cleanest" limestone at Kinnekulle and is therefore used for limestone production. The 8-60 cm thick limestone beds that make up the Täljsten and immediately adjacent strata are stable lithological features that can be traced for hundreds of kilometers through southern Sweden (Fig. 1 and fig. S3). Different beds have been used for different types of industrial stone products, and each bed has a name used by the quarry workers (e.g. Botten, Golvsten, Likhall). These names have remained essentially unchanged for at least a century and some of the names are much older than so. The Swedish name for one of the cleanest beds, Likhall, can be translated with "Corpse Plate" reflecting the fact that corpses were buried under large plates of this bed. In some intervals of the Likhall bed mass occurrences of the cystoid echinoderm *Sphaeronites pomum* occur (fig. S4). The high concentration of these cm-sized fossils probably reflects strong winnowing of the finer fractions through wave action during the sea-level lowstand. The Täljsten interval represents a conspicuous regressive event observed over most of Baltoscandia, (see further e.g., *4, 64-66*), and is traceable also in Laurentia (*67*), Siberia (*68, 69*), Yangtze Platform (*70*) Tarim (*71*), and Gondwana (*6, 72*).

Lindskog et al. (*41*) note that: "it has long been known that excluding color, the characteristics of the uppermost beds of the lower red interval at Kinnekulle are more similar to the Täljsten than to the strata below [see Westergård, 1943 (*73*); Bohlin, 1960 (*74*)], but this knowledge has mostly fallen into oblivion". It is true that the uppermost meter of the lower red limestone at Kinnekulle is similar to the overlying grey Täljsten in being very clean and with coarse grain size. Therefore also this limestone, like the red, 62 cm thick Arkeologen bed, is used for industrial production of limestone slabs. The change towards cleaner and more coarse-grained limestone appears to start close to the -1 m level (relative to the base of the Arkeologen bed), corresponding to when the LCPB event took place (Fig. 4).

Uncertainties in cosmic-ray Ne-21 ages

Published ^{21}Ne cosmic ray exposure ages (*20, 21*) shown in Fig. 3 have uncertainties of about 40% (*20*), mainly reflecting the way ^{21}Ne production rates (P21) had to be determined. P21 uncertainties are relatively large on the one hand due to the lack of a proper shielding correction, i. e. the unknown size of each preatmospheric meteoroid from which the analysed chromite grains were taken as well as an unknown position of these grains within the meteoroid. We assumed a typical preatmospheric size for chondrites (25 cm radius) and a sample position below the surface of 10 cm. On the other hand, also the elemental production rate of ^{21}Ne from Cr is not well known and the concentrations of Mg and Al - although only minor elements in chromite - critically influence P21 values in each individual analysed grain batch, with about 75% of the cosmogenic ^{21}Ne having been produced by spallation of these elements (*20*). In Fig. 3 we show two different ages for each sample. The higher age in each case is based on the P21 values published by Heck et al. (*20*), which rely on the cosmogenic nuclide production model by Leya et al. (*75*) taking into account average Mg and Al concentrations of L chondritic chromites from fossil meteorites (*57*). The concentrations of Mg and Al in chromites from fossil equilibrated L chondrites have relatively low variability (MgO = 2.57±0.83 wt% Avg±1SD; Al_2O_3 = 5.53±0.29 wt% Avg±1SD, (*57*). The lower age is calculated with P21 values determined by Heck et al. (*21*) which reflect the average concentrations of cosmogenic ^{21}Ne in chromite grains separated from five modern chondrite falls with independently determined exposure ages.

Some of the samples shown in Fig. 3 have identical ^{3}He and ^{21}Ne exposure ages, indicating no loss of cosmogenic ^{3}He and ^{21}Ne, whereas other samples - with lower ^{3}He than ^{21}Ne ages -

indicate partial loss of ^{3}He (*20*). We therefore only show ^{21}Ne ages in Fig. 3. A minor loss of cosmogenic ^{21}Ne of these samples cannot strictly be excluded but would not compromise the validity of the extrapolation shown in Fig. 3. As discussed by Heck et al. (*20*), large losses of cosmogenic ^{21}Ne can be excluded, however. Evidence for this is provided by the regular age progression in the sediment column and the agreement of the age difference between samples from the lowest and highest positions, respectively, with the estimated average sedimentation rate of a few millimeters per thousand years based on conventional stratigraphic approaches (see references in *11*). The fact that ^{21}Ne ages of different samples from the same meteorite always agree with each other within error limits provides further evidence for a good retention of cosmogenic ^{21}Ne in our chromite samples. The extrapolation in Fig. 3 assumes constant sedimentation rates throughout the entire sampled section. Uncertainties caused by this assumption are difficult to estimate and have not been included in Fig. 3.

Chrome-spinel results for Lynna River valley section

Here we have also extended the study of Lindskog et al. (*76)* of chrome-spinel abundances across the Lynna River Valley section. Because of a change in customs regulations in Russia we could no longer export limestone samples for acid dissolution in Sweden. Therefore our study of the chrome-spinel distribution across this section is unfinished, however, already the preliminary results show that the trends from the Hällekis section can be reproduced, although not at the same high resolution. In the Lynna River section an increase in abundance of EC grains is first apparent in the sample G1 (fig. S5, data S5). The change in H-L-LL ratio from about equal abundance of each type, to complete dominance of the L chondrites takes place somewhere between samples G1 and 30. The samples G2, G3, G3.5 and G4 where too small (ca. 10 kg each) to provide a sufficient number of chrome-spinel grains for statistically robust interpretations. There is thus presently a 1.5 meter interval that would need to be studied in further detail in order to pinpoint the exact level for the first traces of material from the LCPB breakup. In any case, it is in this 1.5 meter stratigraphic interval where Rasmussen et al. (*4*) show that a shallow-water fauna of brachiopods begins to replace a deeper-water fauna.

Antarctic micrometeorites, sampling, processing and results

The Sør Rondane Mountains within Dronning Maud Land of East Antarctica cover a surface area of approximately 2000 km^2, mainly composed of low- to high-grade metamorphic lithologies that were intruded by various plutonic rocks, e.g. (*77*). Sensitive high-resolution ion microprobe U-Pb zircon ages indicate that the last tectonothermal event in the Sør Rondane mountain range took place 650-500 Ma ago, after which the Sør Rondane Mountains have remained geologically stable (*78*). Ice sheet surfaces reach an elevation of about 1000 meter above sea level (masl) north of the Sør Rondane Mountains and rise to 2500 masl to the South (*77*). Based on a previous field campaign (*79*) and using satellite images together with geological maps, micrometeorite-rich erosional and eolian sediment was sampled from exposed cracked and fissured surfaces at wind-exposed, high altitude (>1650 masl) granitoid summits in the western part of the Sør Rondane Mountains (fig. S6).

Most of the micrometeorites recovered for the purpose of this work were extracted from granitic/gneissic detritus collected at Walnumfjellet (~2450 meter above sea level; S72˚ 07.188’, E24˚ 12.525’). An abraded coherent surface showing glacial striations with an orientation varying between N 0˚E and N 30˚E was determined to have a ^{10}Be exposure age of 1.9±0.2 Ma (*80*). A total of 6.4 kg sediment was collected at Walnumfjellet site #3 using dedicated polypropylene sampling tools and gradually defrosted at the Princess Elisabeth Antarctica station after return from daily field trips. After sample splitting, 2.8 kg of this sediment was processed by washing in milli-Q H_2O and sieving at the Vrije Universiteit Brussel (Brussels, Belgium) to separate size fractions of less than < 125 µm, 125-200 µm, 200-400 µm, 400-800 µm, 800-2000 µm, and > 2000 µm, while the remaining half is kept for reference and other research purposes. All size fractions were subjected to magnetic separation using hand magnets. Using optical microscopy and micro X-ray fluorescence spectrometry (microXRF), 2039 cosmic spherules and 190 partially melted (scoriaceous) and unmelted micrometeorites were handpicked from the four magnetic fractions between 125 and 2000 µm. Based on the inspection of multiple small subsamples, no significant number of residual micrometeorites remain in the non-magnetic fractions. Surficial textural characteristics and diameters were determined for these particles using a FEI ESEM Quanta 200 *e*nvironmental scanning electron microscope at the Royal Belgian Institute of Natural Sciences (Brussels, Belgium; Fig. 5, figs. S7, S8).

Additional but smaller subsamples of sediment (<150 g) from Widerøefjellet (S72˚ 08.746’ E23˚, 16.582’), Svindlandfellet (S72˚ 07.201’, E23˚ 45.689’), and Walnumfjellet were processed at the Astrogeobiology Laboratory of the Department of Physics, Lund University (Lund, Sweden) following a protocol similar to the one described above. Here, the studied size fractions of 80-200 μm, 200-300 μm, 300-500 μm and 500-700 μm led to the recovery of an additional 753 cosmic spherules. The total of 2982 micrometeorites was dissolved in HF acid at Lund University. In the dissolved micrometeorites we found four Cr-rich spinel grains, see further main text and data S4.

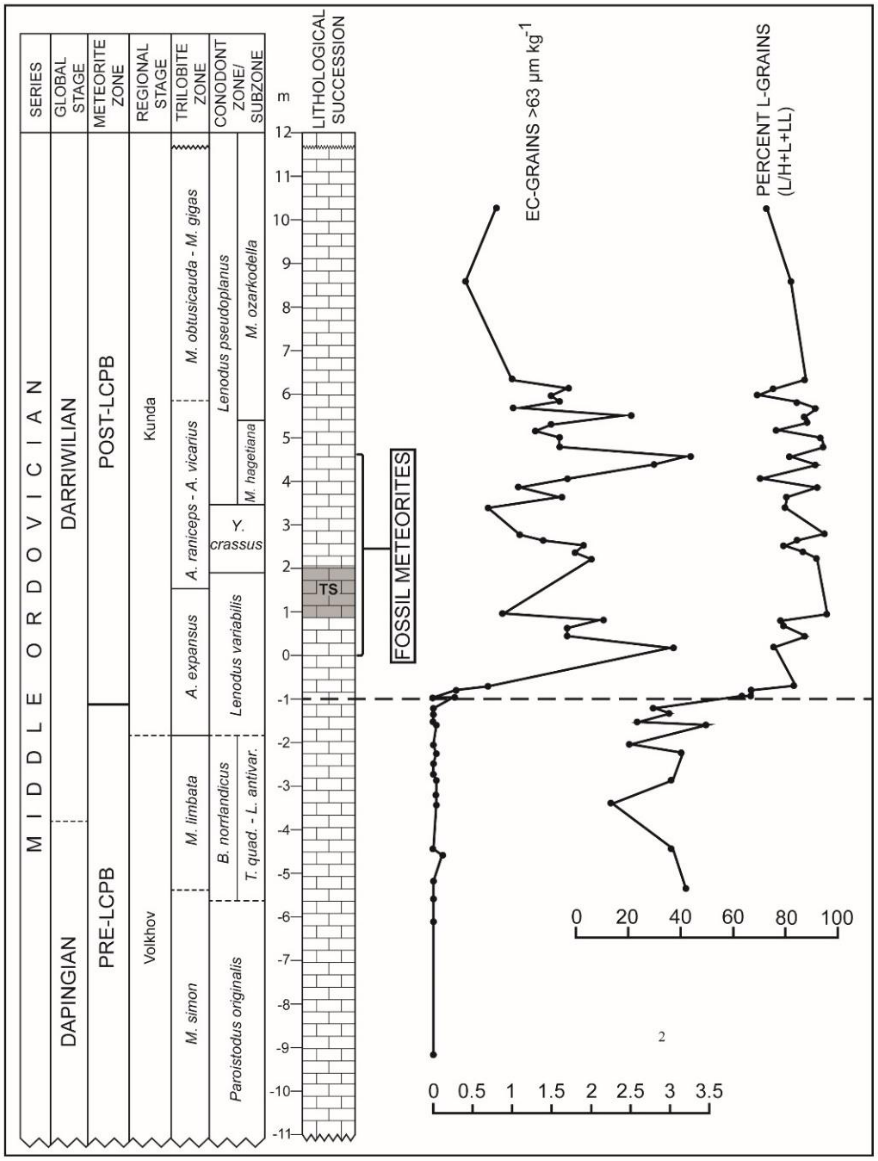

Fig. S1. Distribution of equilibrated, ordinary chondritic chromite (EC) grains through the Hällekis-Thorsberg section. This is the same figure as Figure 2 in the main text, but with the division of chromite grains into H, L, and LL groups based on TiO_2 uncorrected for the 10% overlap between the groups. For more information, see tables S2 and S3. Biostratigraphy in this figure and Figs. 2 and 4 based on (*81*) and references therein.

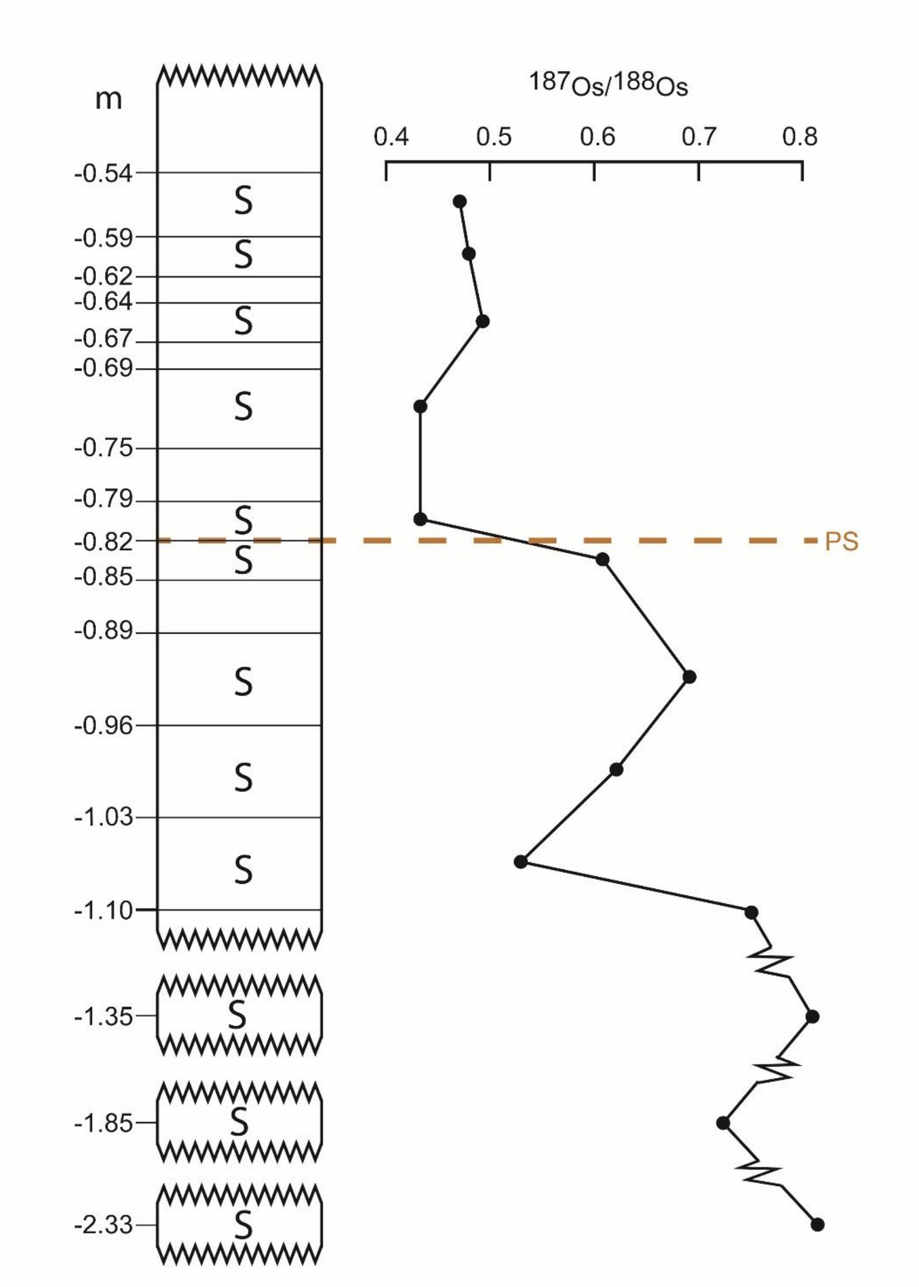

Fig. S2. High-resolution $^{187}Os/^{188}Os$ isotope profile across a proposed discontinuity surface. The figure shows an $^{187}Os/^{188}Os$ isotope profile across a "planar surface" (PS in figure) at -0.82 m below our reference level. The surface is suggested by (*41*) to represent a discontinuity surface. However, the decline in $^{187}Os/^{188}Os$ starts about 20 cm below the "planar surface", and there is no indication in the observed Os isotope trend in support of a significant hiatus at the "planar surface". This is also supported by our chrome-spinel data, and biostratigraphic information.

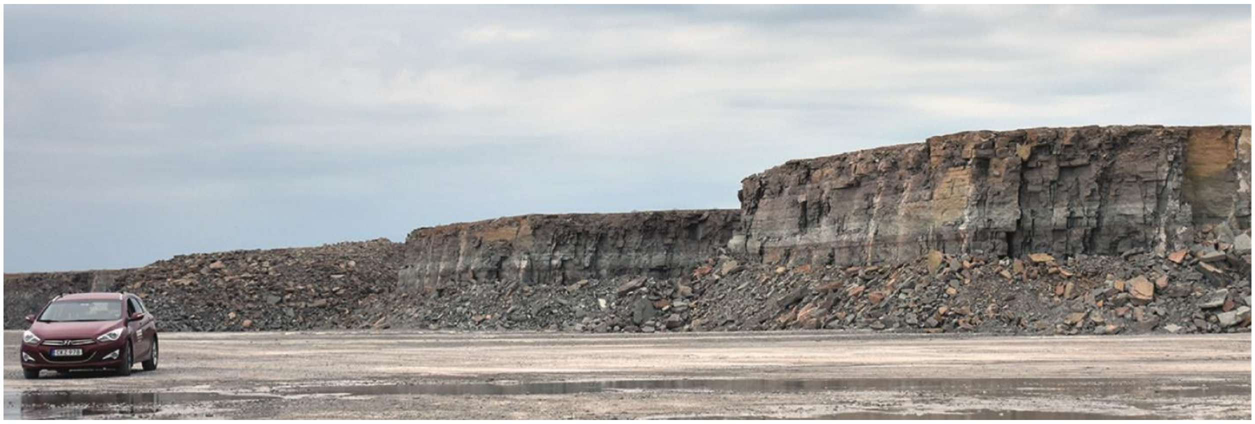

Fig. S3. The gray Täljsten in the Degerhamn Quarry, southern Öland. In this marine, Darriwilian limestone section 300 km southeast of the Hällekis Quarry the Täljsten occurs as a prominent grey band, just like at Hällekis. Even the succession of named beds (Arkeologen, Golvsten, Botten etc.) recognized in the Täljsten at Hällekis can be identified in the Degerhamn quarry, although the thicknesses of the beds are different. The Täljsten interval represents a conspicuous regressive event observed over most of Baltoscandia, (see e.g., *4, 64*-*66*), and is traceable also in Laurentia (*67*), Siberia (*68*, *69*), Yangtze Platform (*70*) Tarim (*71*), and Gondwana (*6*, *72*). (Photo credit: Birger Schmitz, Lund University)

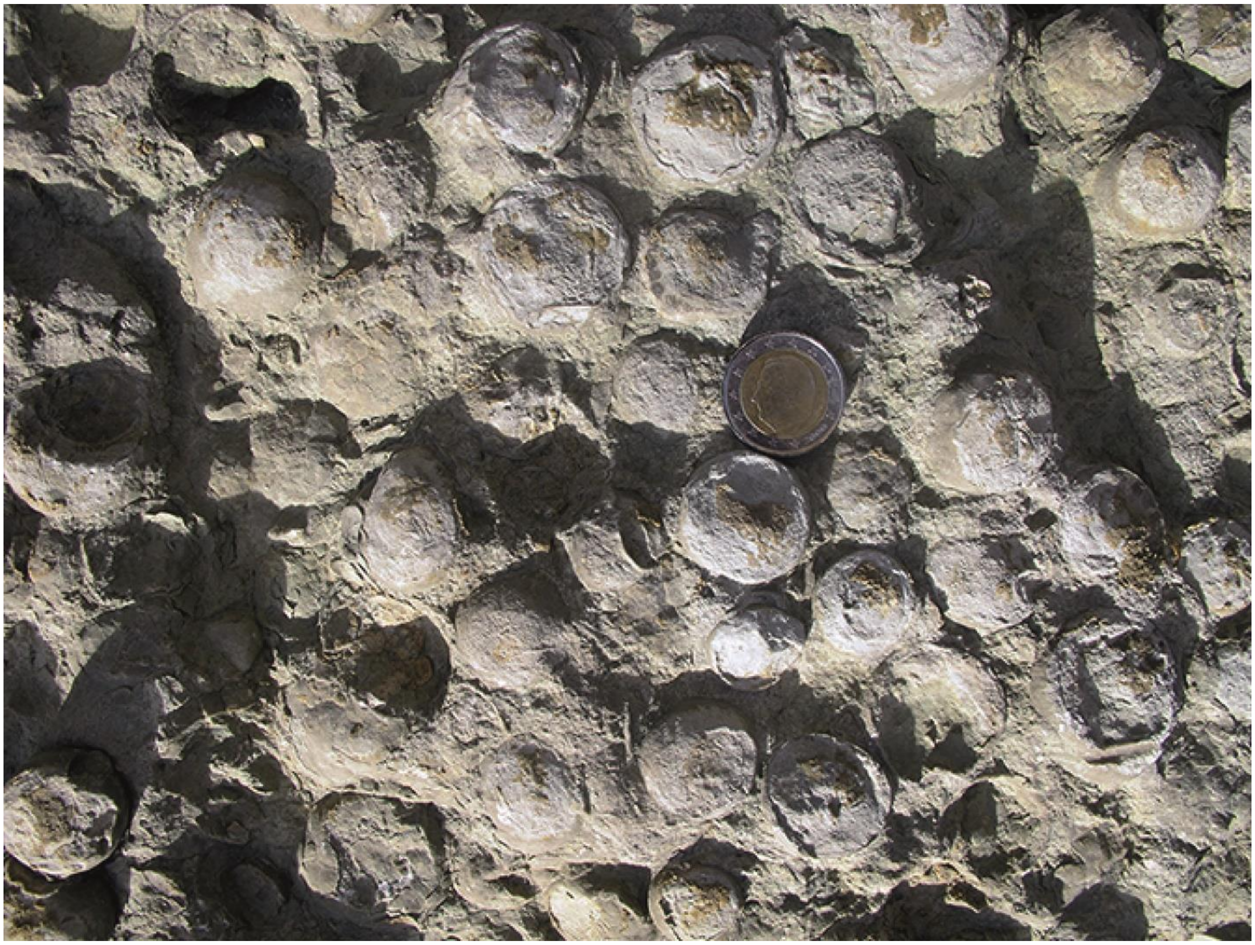

Fig. S4. Cystoid echinoderms in the Likhall bed of the Täljsten. Mass occurrences of 1.5 - 4 cm in diameter cystoid echinoderms can be found in the Likhall bed. Often the upper part of the cystoids have been destroyed, whilst the lower part is embedded in calcareous mud. "(Photo credit: Fredrik Terfelt, Lund University)

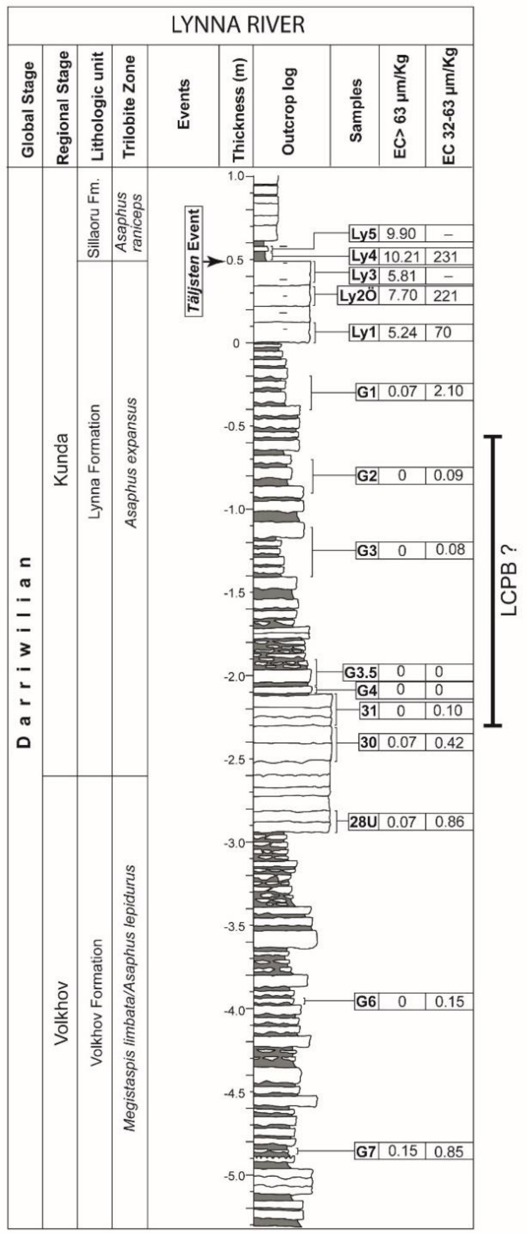

Fig. S5. Distribution of extraterrestrial chromite across the Lynna River section. The first abundant equilibrated ordinary chondritic chromite (EC) grains were found in sample G1. The EC grains in this sample are completely dominated by L-chondritic grains (data S5). In sample 30 the seven EC grains recovered represent a mix of H-, L- and LL-chondritic grains. The samples 31, G4, G3.5, G3 and G2 were too small to yield a sufficient number of extraterrestrial chromite grains for robust interpretations. There is thus an uncertainty over 1.5 m of section about the exact level corresponding to the LCPB breakup. For further details, see data S5.

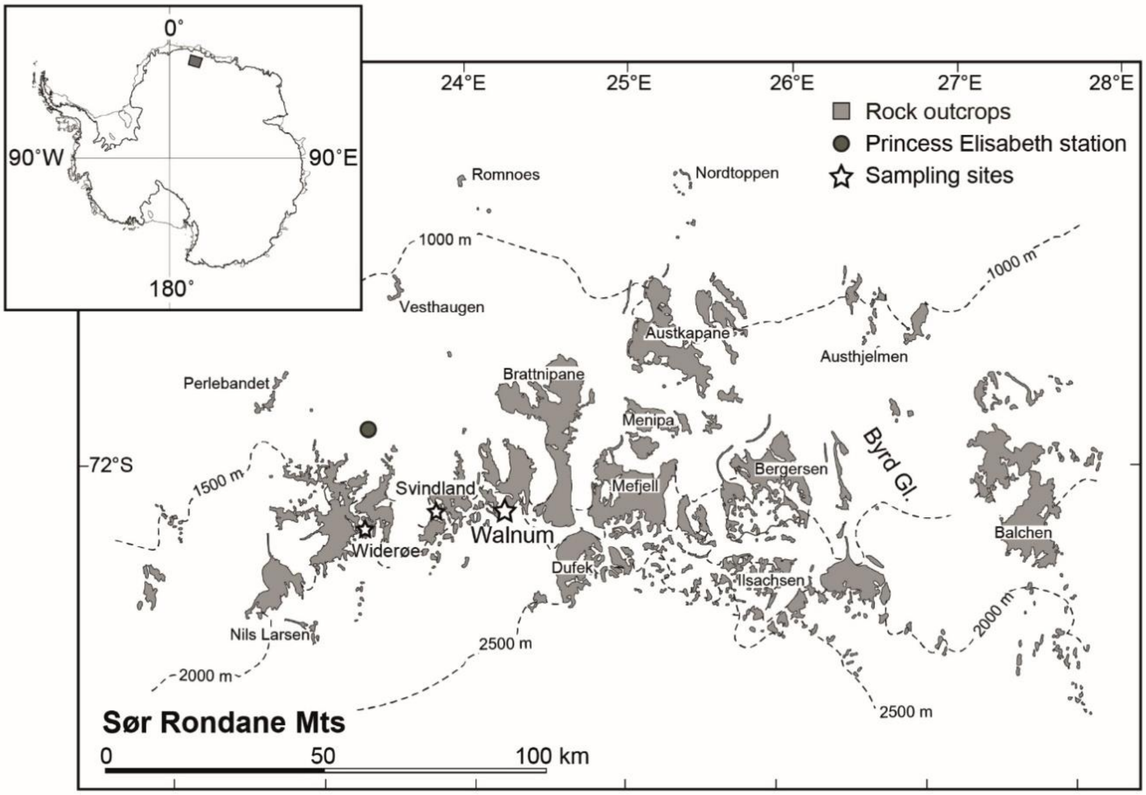

Fig. S6. Map of Antarctic micrometeorite localities. Location of the Sør Rondane Mountains within Antarctica (inset), the Belgian Princess Elisabeth Antarctic research station (filled circle), and the Walnumfjellet, Widerøefjellet, and Svindlandfjellet micrometeorite accumulation sites that were processed in this work (stars). Map adapted after Suganuma et al. (*80*).

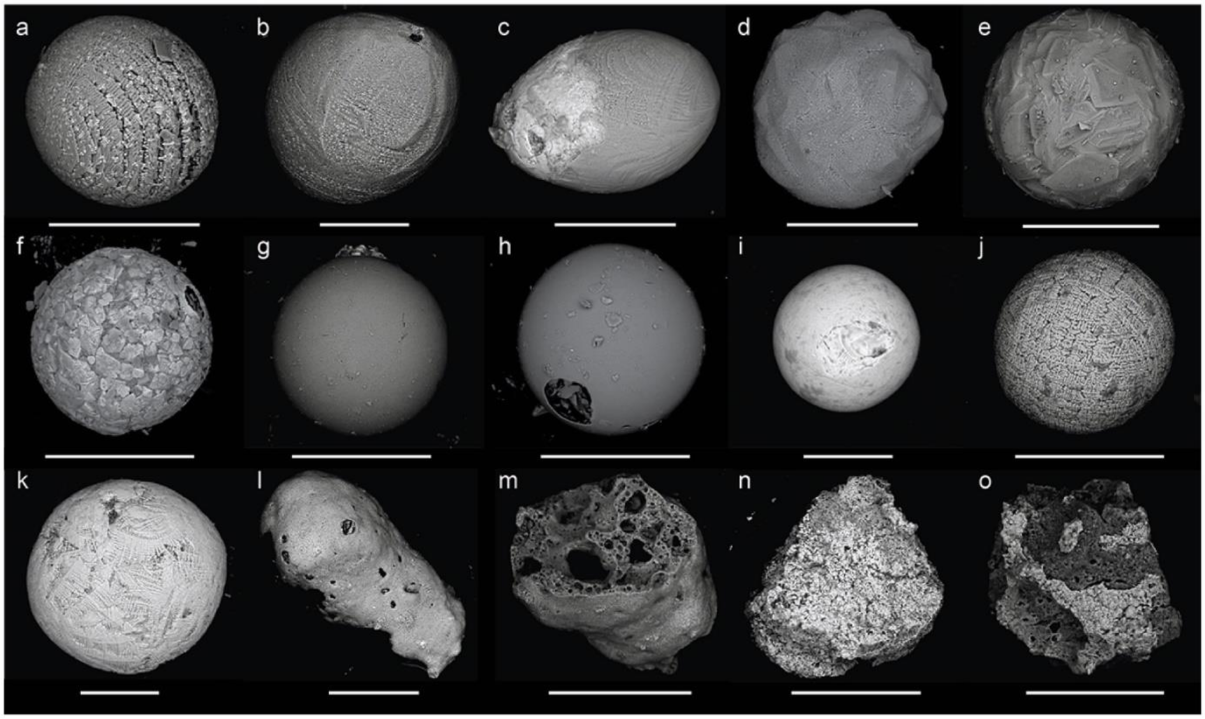

Fig. S7. Back-scattered electron images of Antarctic micrometeorites. Shown are a representative subset of micrometeorites dissolved in this work, illustrating the different textural and chemical types, particle sizes, and range in alteration and preservation states found at Walnumfjellet. White bars represent 100 µm. Based on surficial characteristics, the following micrometeorite classes have been documented: *(a-b)* Barred olivine cosmic spherules with typical striations. *(c-d)* Cryptocrystalline cosmic spherules, with turtle-back (polyhedral-like) morphology in *(d)*. *(e-f)* Porphyritic olivine cosmic spherules. *(g-h)* Glass cosmic spherules. *(i)* I-type spherule. *(j-k)* G-type cosmic spherules consisting of bright magnetite in dark silicate glass. *(l-m)* Partially melted or scoriaceous micrometeorites, fine-grained in the case of *(m)*. *(n-o)* Unmelted micrometeorites.

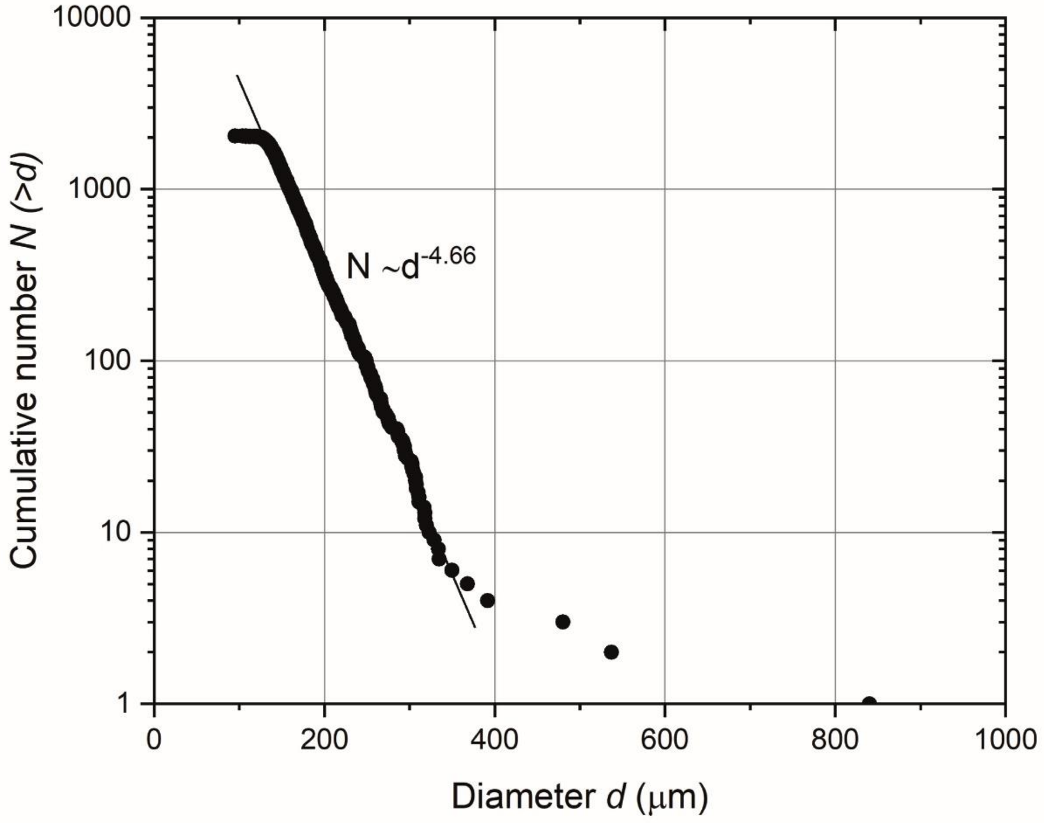

Fig. S8. Size distribution of Antarctic micrometeorites. Cumulative size distribution for cosmic spherules recovered from Walnumfjellet in the Sør Rondane Mountains. The distribution is fitted to a power law function between 127 μm and 334 μm with an exponent of -4.66 but shows divergence from the power law at both high and low diameters. The size distributions of spherules studied at Walnumfjellet are consistent across different size fractions and follow a power law distribution with an exponent similar to that determined for the Transantarctic Mountain collection (*82*) and the South Pole Water Well collection (*83, 84*), implying an accumulation mechanism dominated by direct infall.

Table S1. Chrome-spinel distribution through the Hällekis-Thorsberg section. Samples, their weights and contents of different types of chrome-spinel grains in the 32-63 and >63 μm size fractions. See Materials and Methods section for explanation of the EC, OtC-V and OtC division of the grains. The zero level in the profile is at the base of the Arkeologen bed.

Meter level	Sample weight (kg)	No. of EC-grains > 63 μm	EC-grains > 63 μm/kg	No. of EC-grains 32-63 μm	EC-grains 32-63 μm/kg	No. of OtC-V grains > 63 μm	OtC-V-grains > 63 μm/kg	No. of OtC-V grains 32-63 μm	OtC-V grains 32-63 μm/kg	No. of OtC-grains > 63 μm	OtC-grains > 63 μm/kg	No. of OtC-grains 32-63 μm	OtC-grains 32-63 μm/kg
+10.2-10.3	18.8	15	0.8	-	-	0	0	-	-	5	0.3	-	-
+8.50-8.65	28.0	11	0.4	-	-	0	0	-	-	1	0.04	-	-
+6.21-6.45	8.4	8	1.0	-	-	0	0	-	-	39	4.6	-	-
+6.06-6.21	9.9	17	1.7	-	-	1	0.1	-	-	33	3.3	-	-
+5.90-6.06	8.6	13	1.5	-	-	0	0	-	-	43	5.0	-	-
+5.76-5.90	8.3	13	1.6	-	-	1	0.1	-	-	12	1.4	-	-
+5.59-5.76	10.9	12	1.1	-	-	0	0	-	-	8	0.7	-	-
+5.43-5.59	9.9	24	2.4	-	-	0	0	-	-	56	5.7	-	-
+5.26-5.43	11.1	18	1.5	-	-	0	0	-	-	10	0.9	-	-
+5.09-5.26	9.9	13	1.3	-	-	1	0.1	-	-	5	0.5	-	-
+4.90-5.09	11.1	18	1.6	-	-	0	0	-	-	3	0.3	-	-
+4.68-4.90	13.1	21	1.6	-	-	1	0.1	-	-	18	1.4	-	-
+4.47-4.68	14.2	45	3.2	-	-	0	0	-	-	20	1.4	-	-
+4.16-4.47	12.9	36	2.8	-	-	0	0	-	-	99	7.7	-	-
+3.96-4.16	12.2	21	1.7	-	-	1	0.1	-	-	2	0.2	-	-
+3.76-3.96	12.2	13	1.1	-	-	0	0	-	-	1	0.1	-	-
+3.52-3.76	15.9	26	1.6	-	-	0	0	-	-	5	0.3	-	-
+3.27-3.52	13.6	10	0.7	-	-	0	0	-	-	9	0.7	-	-
+2.73-2.83	19.0	21	1.1	-	-	0	0	-	-	5	0.3	-	-
+2.58-2.73	19.0	26	1.4	-	-	1	0.05	-	-	24	1.3	-	-
+2.50-2.58	10.7	20	1.9	-	-	0	0	-	-	2	0.2	-	-
+2.29-2.48	13.0	23	1.8	-	-	0	0	-	-	7	0.5	-	-
+2.15-2.29	11.8	24	2.0	-	-	0	0	-	-	2	0.2	-	-
+0.87-1.05	24.6	23	0.9	-	-	2	0.1	-	-	32	1.3	-	-
+0.77-0.87	13.3	28	2.1	-	-	1	0.1	-	-	3	0.2	-	-
+0.62-0.77	17.7	30	1.7	-	-	0	0	-	-	1	0.06	-	-
+0.32-0.56	24.0	41	1.7	-	-	0	0	-	-	3	0.1	-	-
+0.08-0.32	26.0	79	3.0	-	-	1	0.04	-	-	48	1.8	-	-
-0.54-0.82	4.3	3	0.7	40	9.3	0	0	7	1.6	2	0.5	42	9.8
-0.77-0.82	22.8	6	0.3	-	-	0	0	-	-	2	0.1	-	-
-0.82-0.97	25.4	0	0	-	-	0	0	-	-	1	0.04	-	-
-0.82-1.10	5.5	0	0	12	2.2	0	0	1	0.2	0	0	28	5.1
-0.82-1.05	62.0	16	0.26	>119	>1.9	1	0.02	>10	>0.2	7	0.1	>141	>2.3
-1.05-1.35	101.0	2	0.02	21	0.2	5	0.05	9	0.09	3	0.03	127	1.3
-1.22-1.42	103.0	2	0.02	20	0.2	1	0.01	7	0.07	2	0.02	93	0.9
-1.47-1.69	28.0	1	0.04	11	0.4	0	0	1	0.4	6	0.2	26	0.9
-1.27-1.77	103.0	1	0.01	32	0.3	2	0.02	10	0.1	5	0.05	116	1.1
-1.97-2.12	26.0	0	0	14	0.5	0	0	1	0.04	0	0	52	2.0
-2.12-2.30	32.0	1	0.03	26	0.8	0	0	5	0.2	1	0.03	51	1.6
-2.35-2.60	31.7	0	0	-	-	0	0	-	-	0	0	-	-
-2.60-3.10	104.0	4	0.04	55	0.5	4	0.04	4	0.04	0	0	49	0.5
-2.60-2.85	30.2	0	0	-	-	0	0	-	-	0	0	-	-
-3.12-3.27	28.1	1	0.04	16	0.6	1	0.04	2	0.07	1	0.04	31	1.1
-3.27-3.45	27.7	1	0.04	13	0.5	0	0	3	0.11	0	0	19	0.7
-4.30-4.50	28.1	0	0	8	0.3	0	0	1	0.04	0	0	14	0.5
-4.50-4.58	20.6	2	0.1	7	0.3	0	0	0	0	0	0	11	0.3
-5.00-5.25	30.5	0	0	-	-	0	0	-	-	0	0	-	-
-5.45-5.70	28.0	0	0	-	-	0	0	-	-	0	0	-	-
-5.88-6.08	23.1	0	0	3	0.1	1	0.04	3	0.1	1	0.04	31	1.3
-6.08-6.18	20.0	0	0	2	0.1	0	0	3	0.1	0	0	18	0.9
-9.05-9.15	26.4	0	0	-	-	0	0	-	-	0	0	-	-

Table S2. Extraterrestrial chromite division below reference level in Hällekis section. Samples below the reference (zero) level in the Hällekis section and their content of EC grains, and the division of the EC grains in H, L and LL groups based on their TiO_2 content. For the latter division both uncorrected values, and values corrected for 10% overlap between the three groups are given, see further Materials and Methods section. The zero level in the profile is at the base of the Arkeologen bed. The data from the literature represent uncorrected values.

Meter level	Sample weight (kg)	No. of EC-grains > 63 µm	EC-grains > 63 µm/kg	No. of EC-grains 32-63 µm	EC-grains 32-63 µm/kg	No. of H-chondritic grains	Percent H-chondritic grains	No. of L-chondritic grains	Percent L-chondritic grains	No. of LL-chondritic grains	Percent LL-chondritic grains
-0.54-0.82	4.3	3	0.7	40	9.3	7	16	36	84	0	0
-0.77-0.82	22.8	6	0.3	-	-	2		4		0	
Sum:						9	18	40	82	0	0
Corrected:						5.9	12	47.1	96	-4	-8
-0.82-1.10	5.5	0	0	12	2.2	0		8		4	
-0.82-1.05	62.0	16	0.26	70	-	18		55		13	
Sum:						18	18	63	64	17	17
Corrected:						13.5	14	72.1	73	12.4	13
-1.05-1.35	101.0	2	0.02	21	0.2	12		7		4	
-1.22-1.42	103.0	2	0.02	20	0.2	12		8		2	
-1.47-1.69	28.0	1	0.04	11	0.4	5		6		1	
-1.27-1.77	103.0	1	0.01	32	0.3	9		8		16	
Sum:						38	42	29	32	23	26
Corrected:						38.9	43	28.7	32	22.4	25
-1.97-2.12	26.0	0	0	14	0.5	4		3		7	
-2.12-2.30	32.0	1	0.03	26	0.8	10		11		6	
Sum:						14	34	14	34	13	32
Corrected:						14	34	14.1	34	12.9	32
-2.60-3.10	104.0	4	0.04	55	0.5	18		22		19	
-3.12-3.27	28.1	1	0.04	16	0.6	8		4		5	
-3.27-3.45	27.7	1	0.04	13	0.5	6		2		6	
-4.30-4.50	28.1	0	0	8	0.3	3		3		2	
Sum:						35	36	31	32	32	33
Corrected:						35.4	36	30.5	31	32.1	33
-4.50-4.58	20.6	2	0.1	7	0.3	4		5		0	
-5.88-6.08	23.1	0	0	3	0.1	0		0		3	
-6.08-6.18	20.0	0	0	2	0.1	0		1		1	
Sum:						4		6		4	
Corrected:						3.8	27	6.4	46	3.8	27
Total sum below -1.05 m (n=243):						91	37	80	33	72	30
Corrected:						92.1	38	79.7	33	71.2	29
Post-LCPB Heck et al. (*56*) n=119:						10	8	102	86	7	6
Post-LCPB Martin et al. (*31*) n=444:						63	14	361	81	20	5
Pre-LCPB GAP7 Schmitz et al. (*55*) n=215:						80	37	71	33	64	30

Table S3. Extraterrestrial chromite division above reference level in Thorsberg section. Samples above the reference (zero) level in the Thorsberg section and their content of EC grains, and the division of the EC grains in H, L and LL groups based on their TiO_2 content. For the latter division both uncorrected values, and values corrected for 10% overlap between the three groups are given, see further Materials and Methods section. The zero level in the profile is at the base of the Arkeologen bed.

Meter level	Sample weight (kg)	No. of EC-grains > 63 µm	EC-grains > 63 µm/kg	No. of H-chondritic grains	Percent H-chondritic grains	No. of L-chondritic grains	Percent L-chondritic grains	No. of LL-chondritic grains	Percent LL-chondritic grains
+10.2-10.3	18.8	15	0.8	1	7	11	73	3	20
Corrected				0	0	12.8	85	2.2	15
+8.50-8.65	28.0	11	0.4	0	0	9	82	2	18
Corrected				-0.9	-8	10.6	96	1.3	12
+6.21-6.45	8.4	8	1.0	1	12	7	88	0	0
Corrected				0.4	5	8.3	104	-0.7	-9
+6.06-6.21	9.9	17	1.7	4	24	13	76	0	0
Corrected				3.1	18	15.2	90	-1.3	-8
+5.90-6.06	8.6	13	1.5	2	15	9	70	2	15
Corrected				1.3	10	10.4	80	1.3	10
+5.76-5.90	8.3	13	1.6	2	15	11	85	0	0
Corrected				1.1	8	13	100	-1.1	-8
+5.59-5.76	10.9	12	1.1	1	8	11	92	0	0
Corrected				0	0	13.1	109	-1.1	-9
+5.43-5.59	9.9	24	2.4	3	12	21	88	0	0
Corrected				1.2	5	24.9	104	-2.1	-9
+5.26-5.43	11.1	18	1.5	2	11	16	89	0	0
Corrected				0.6	3	19	106	-1.6	-9
+5.09-5.26	9.9	13	1.3	2	15	10	77	1	8
Corrected				1.2	9	11.7	90	0.1	1
+4.90-5.09	11.1	18	1.6	1	6	17	94	0	0
Corrected				-0.6	-3	20.3	113	-1.7	-10
+4.68-4.90	13.1	21	1.6	1	5	20	95	0	0
Corrected				-0.9	-4	23.9	114	-2	-10
+4.47-4.68	14.2	45	3.2	4	9	37	82	4	9
Corrected				0.7	1.5	43.6	97	0.7	1.5
+4.16-4.47	12.9	36	2.8	2	5	33	92	1	3
Corrected				-1.1	-3	39.3	109	-2.2	-6
+3.96-4.16	12.2	21	1.7	2	10	15	71	4	19
Corrected				0.7	3	17.4	83	2.9	14
+3.76-3.96	12.2	13	1.1	1	8	12	92	0	0
Corrected				-0.1	-1	14.3	110	-1.2	-9
+3.52-3.76	15.9	26	1.6	4	15	21	81	1	4
Corrected				2.3	9	24.7	95	-1	-4
+3.27-3.52	13.6	10	0.7	2	20	8	80	0	0
Corrected				1.4	14	9.4	94	-0.8	-8
+2.73-2.83	19.0	21	1.1	1	5	20	95	0	0
Corrected				-0.9	-4	23.9	114	-2	-10
+2.58-2.73	19.0	26	1.4	2	8	22	85	2	8
Corrected				0	0	26	100	0	0
+2.50-2.58	10.7	20	1.9	2	10	16	80	2	10
Corrected				0.6	3	18.8	94	0.6	3
+2.29-2.48	13.0	23	1.8	3	13	20	87	0	0
Corrected				1.3	6	23.7	103	-2	-9
+2.15-2.29	11.8	24	2.0	2	8	22	92	0	0
Corrected				0	0	26.2	109	-2.2	-9
+0.87-1.05	24.6	23	0.9	0	0	22	96	1	4
Corrected				-2.2	-9	26.3	114	-1.1	-5
+0.77-0.87	13.3	28	2.1	3	11	22	79	3	11
Corrected				1.1	4	25.8	92	1.1	4
+0.62-0.77	17.7	30	1.7	4	13	24	80	2	7
Corrected				2	7	28.2	94	-0.2	-1
+0.32-0.56	24.0	41	1.7	3	7	36	88	4	10
Corrected				-0.3	-1	42.5	99	0.8	2
+0.08-0.32	26.0	79	3.0	4	5	60	76	15	19
Corrected				-1.6	-2	70.1	89	10.5	13
**Total:**	**651**			**59**	**9**	**545**	**84**	**47**	**7**
**Corrected:**				**10.4**	**1.5**	**643.4**	**99**	**-2.8**	**-0.5**

Table S4. Published abundances of micrometeorite types from different collections. The abundances of S-, I-, and G-type cosmic spherules are given as a percentage of the total number of spherules, while the abundances of Po-, BO-, C-, and V-type spherules are given as a percentage of S-type cosmic spherules.

	WN-MT#3	TAM[a]	Walcott Névé[a]	Larkman[b]	SPWW[c]	CONCORDIA[d]	Indian Ocean[e]
CS	91.5	96.0	100.0	97.2	na	44	100.0
S	95.7	96.0	98.0	92.1	97.0	na	90.7
Po	35.5	na	na	34.0	23.0	na	25.1
BO	32.6	na	na	21.7	41.0	na	50.1
Cc	26.7	na	na	24.9	12.0	na	7.5
V	0.9	na	na	19.4	17.0	na	7.9
I	3.0	3.0	1.0	5.4	2.0	na	5.5
G	1.3	1.0	1.0	2.3	1.0	na	3.5
ScMM	3.2	na	na	1.7	na	22	na
UMM	5.3	4.0	na	1.1	na	34	na

[a]Transantarctic Mountains (*82*)

[b](*85*)

[c]South Pole Water Well (*83*). Note that Po-types spherules include relict-bearing and vesicular spherules.

[d](*86*)

[e](*87*)

na = not available; CS = cosmic spherules; S = silicate-dominated; I = iron-dominated; G = intermediate between S- and I-type; Po = porphyritic olivine; BO = barred olivine; C = cryptocrystalline; V = glassy; ScMM = scoriaceous micrometeorites; UMM = unmelted micrometeorites.

Table S5. Poynting-Robertson transfer times (Ma) from the outer solar system to Earth. Typical transfer times from the inner rim of the asteroid belt (1.9 AU), the division between the inner and middle asteroid belt (2.5 AU), the division between the middle and outer asteroid belt (2.8 AU), the outer rim of the asteroid belt (3.5 AU), the Jupiter trojans (5.2 AU) and the inner rim of the Kuiper belt (30 AU), based on Burns et al. (*88*) and assuming $\beta = 1$. Densities of 4.4 g/cm^3 and 3.0 g/cm^3 were used for chromite and silicate, respectively. All ages are given in millions of years (Ma).

Diameter, type	1.9 AU	2.5 AU	2.8 AU	3.5 AU	5.2 AU	30 AU
40 µm, chromite	0.16	0.33	0.42	0.70	1.6	56
60 µm, chromite	0.24	0.49	0.64	1.0	2.4	83
80 µm, chromite	0.32	0.65	0.85	1.4	3.2	110
100 µm, silicate	0.28	0.55	0.72	1.2	2.8	95
200 µm, silicate	0.55	1.1	1.4	2.4	5.5	190
300 µm, silicate	0.83	1.7	2.2	3.6	8.2	280
1000 µm, silicate	2.8	5.5	7.2	12	27	950
