# Supplementary material for: An extraterrestrial trigger for the mid-Ordovician ice age: Dust from the breakup of the L-chondrite parent body
Source: Sci Adv. 2019 Sep 18;5(9):eaax4184. doi: 10.1126/sciadv.aax4184 (PMC6750910; doi:10.1126/sciadv.aax4184)
Supplement: Data file S1 [file aax4184_Data_file_S1.pdf]

DATA S1: Hällekis-Thorsberg section - chrome-spinel chemical results

Element composition (wt%) of chrome-spinel grains from a composite section at Kinnekulle (Hällekis and Thorsberg quarries). Most data are from the present study, but some data for grains >63 µm from Schmitz and Häggström (2006) (*88*) and Häggström and Schmitz (2007) (*89*). All analyses on polished grains. In the table: n.d. = not detected. Total weight percentages below ~97 wt% and above ~103 wt% are marked in red.

Sample depths are given relative to the base of the Arkeologen bed. All samples from below the reference zero level are from the Hällekis Quarry, and all samples above this level are from the Thorsberg Quarry. Because of the high concentrations of extraterrestrial grains in the sediment following the LCPB event the samples from the Thorsberg Quarry were only studied in the >63 µm fraction.

Division of grains in different groups

Chrome-spinel grains are categorized as follows:

- EC – Grains from equilibrated ordinary chondrites (petrological types 4-6) with oxide weight percentages within the ranges of Cr_2O_3: ~53.0-62.0, FeO: ~23.0-32.0, Al_2O_3: ~4.5-8.5, MgO: ~1.3-4.5, V_2O_3: ~0.55-0.95, and TiO_2: ~1.40-4.50 (for more extensive discussions, see *11*, p. 127). Small deviations from these values in one or two oxides are marked with red color in the table. The FeO values of EC grains can sometimes be lower than 23% because of replacement by MnO and/or ZnO, see (*57*).
- OtC-V – Other chrome spinel, i.e. grains that do not have the typical equilibrated ordinary chondritic composition, but contain ≥0.45 wt% V_2O_3 and a Cr_2O_3/FeO ratio ≥1.45, indicating a likely meteoritic origin.
- OtC – Other chrome-spinel grains, but with V_2O_3 <0.45 wt% or ≥0.45 wt% V_2O_3 together with a Cr_2O_3/FeO ratio <1.45. The OtC grains are likely of terrestrial origin.

The type of grains here referred to as OtC grains have in our previous studies been referred to as OC grains, but we have changed acronym in order to avoid confusion with OC as being used for "ordinary chondrites" in other research.

Results

+10.20 to +10.30 m

Sample weight 18.8 kg.

EC grains >63 µm

Grain	MgO	Al_2O_3	TiO_2	V_2O_3	Cr_2O_3	MnO	FeO	ZnO	Total

Cr 1	6.27	4.89	2.66	0.67	61.75	0.49	23.01	0.40	100.1
Cr 2	2.81	6.08	3.09	0.70	61.07	0.80	26.44	0.50	101.5
Cr 4	2.82	5.92	3.01	0.69	59.27	0.82	28.44	n.d.	101.0
Cr 5	3.61	6.15	3.52	0.69	60.18	0.86	25.02	n.d.	100.0
Cr 6	2.87	5.78	3.34	0.78	58.65	0.81	27.49	0.41	100.1
Cr 7	3.36	5.59	3.56	0.72	60.09	0.62	27.27	n.d.	101.2
Cr 8	2.50	4.70	3.18	0.85	60.06	1.00	28.32	0.68	101.3
Cr 9	2.96	5.82	3.39	0.66	59.17	0.93	27.91	n.d.	100.9
Cr 11	2.80	5.71	3.31	0.69	59.96	0.81	27.73	0.49	101.5
Cr 12	4.29	6.66	2.03	0.66	60.83	1.24	24.68	0.98	101.4
Cr 13	3.68	5.67	3.21	0.75	58.78	0.97	26.29	0.38	99.74
Cr 14	6.07	5.84	2.78	0.70	59.16	0.70	24.44	n.d.	99.68
Cr 15	2.89	5.49	3.23	0.75	59.09	1.02	27.86	0.42	100.8
Cr 16[§]	3.35	6.31	2.80	0.87	59.74	0.71	25.76	0.44	100.7
Cr 18	2.54	5.96	3.88	0.86	57.81	0.75	28.80	n.d.	100.6

[§]Also NiO 0.71 wt%.

OtC grains >63 μm

Grain	MgO	Al_2O_3	TiO_2	V_2O_3	Cr_2O_3	MnO	FeO	ZnO	Total
Cr 3	11.03	17.15	n.d.	n.d.	53.13	0.52	17.94	n.d.	99.77
Cr 10	9.66	13.38	n.d.	n.d.	52.38	0.52	23.99	n.d.	99.94
Cr 17	6.20	16.41	n.d.	n.d.	48.13	0.74	28.18	0.42	100.1
Cr 19	18.09	23.18	n.d.	n.d.	40.07	0.52	17.53	n.d.	99.39
Cr 20	9.40	9.01	0.32	n.d.	56.86	n.d.	23.45	n.d.	99.05

+8.50 to +8.65 m

Sample weight 28.0 kg. From Schmitz and Häggström (2006).

EC grains >63 μm

Grain	MgO	Al_2O_3	TiO_2	V_2O_3	Cr_2O_3	MnO	FeO	ZnO	Total
1	1.61	5.54	2.78	0.69	60.58	0.87	26.65	0.43	99.13
2	2.37	6.25	3.47	0.76	60.74	0.94	24.52	0.30	99.35
3	4.13	6.40	2.54	0.84	60.47	0.92	24.03	0.35	99.68
4	2.84	5.78	3.49	0.72	60.66	0.94	25.13	0.64	100.2
5	2.50	6.25	3.10	0.78	62.47	0.93	23.01	0.56	99.61
6	2.51	6.20	3.12	0.72	59.34	0.97	26.60	0.33	99.80
7	2.81	5.87	3.27	0.69	58.39	1.13	26.95	0.48	99.60
8	4.32	6.45	3.14	0.80	60.80	0.81	22.77	0.23	99.32
9	1.97	6.44	3.11	0.76	60.83	0.97	25.08	0.26	99.42
10	2.82	6.13	3.01	0.73	60.41	0.95	25.00	0.45	99.50
11	2.45	6.05	2.96	0.76	59.20	1.01	26.43	0.33	99.19

OtC grains >63 μm

Grain	MgO	Al_2O_3	TiO_2	V_2O_3	Cr_2O_3	MnO	FeO	ZnO	Total
1	2.15	9.32	0.67	n.d.	49.15	0.80	37.30	n.d.	99.44

+6.21 to +6.45 m

Sample weight 8.4 kg.

EC grains >63 μm

Grain	MgO	Al_2O_3	TiO_2	V_2O_3	Cr_2O_3	MnO	FeO	ZnO	Total
85-1	2.03	5.33	2.75	0.82	58.74	0.52	27.16	0.47	97.81
85-5	4.06	5.04	2.35	0.58	57.74	0.48	26.90	0.38	97.52
85-14	2.25	6.03	3.33	0.68	58.78	0.39	26.89	0.27	98.63
85-22	1.74	5.59	3.07	0.71	59.13	0.42	26.91	0.62	98.19
85-29	2.04	5.57	3.14	0.67	58.75	0.45	27.07	1.03	98.73
85-36	2.20	5.28	2.66	0.69	57.80	0.49	29.86	0.45	99.42
85-40	2.69	5.21	2.97	0.74	57.68	0.62	28.70	0.51	99.12
85-47	2.43	5.44	3.16	0.73	59.18	0.49	28.45	0.36	100.2

OtC grains >63 μm

Grain	MgO	Al_2O_3	TiO_2	V_2O_3	Cr_2O_3	MnO	FeO	ZnO	Total
85-2	9.82	16.17	0.71	0.21	41.45	0.15	27.47	0.11	96.09
85-3	9.58	8.22	0.54	0.10	55.52	0.14	22.31	0.29	96.71
85-4	9.98	9.27	0.41	0.14	54.62	0.10	22.08	0.10	96.70
85-6	9.16	8.85	0.28	0.04	55.60	0.17	23.08	0.06	97.24
85-7	8.62	24.00	0.74	0.17	35.68	0.14	26.80	0.09	96.24
85-8	15.78	39.58	0.56	0.13	20.30	0.10	21.52	0.07	98.03
85-9	9.03	7.95	0.31	0.11	58.49	0.13	21.16	0.09	97.28
85-10	10.15	10.18	0.27	0.06	55.05	0.16	22.42	0.08	98.38
85-11	15.87	41.77	0.50	0.10	20.44	0.11	19.90	0.12	98.81
85-12	9.89	16.12	0.09	0.20	53.16	0.20	19.23	0.19	99.07
85-13	9.20	11.90	0.54	0.10	50.68	0.12	24.76	0.04	97.35
85-15	10.06	9.31	0.42	0.13	54.30	0.13	22.69	0.07	97.10
85-16	6.93	6.66	0.23	0.08	62.06	0.11	20.00	0.32	96.39
85-17	9.88	6.29	0.21	0.11	57.07	0.29	22.87	0.14	96.85
85-18	7.70	16.16	1.28	0.33	23.51	0.23	47.79	0.07	97.06
85-19	5.96	15.61	0.21	0.16	45.86	0.20	28.44	0.13	96.58
85-20	7.58	9.50	0.68	0.10	49.84	0.16	29.21	0.09	97.15
85-21	9.48	6.96	0.32	0.08	61.34	0.10	17.57	0.44	96.29
85-23	9.78	10.35	0.42	0.07	54.41	0.11	22.26	0.08	97.47
85-24	8.87	7.26	0.37	0.07	60.50	0.09	18.91	0.10	96.17
85-25	8.52	9.71	0.42	0.06	55.24	0.11	22.53	0.11	96.69
85-26	5.13	7.45	0.61	0.09	53.11	0.22	30.68	0.23	97.53
85-27	10.70	23.27	0.66	0.22	37.31	0.13	23.41	0.08	95.77
85-28	10.46	23.11	0.11	0.16	43.19	0.15	20.49	0.27	97.95
85-30	14.72	39.56	0.53	0.15	20.42	0.13	21.34	0.03	96.88
85-31	7.22	8.32	0.57	0.28	51.29	0.10	27.72	0.07	95.57
85-32	7.55	12.74	0.95	0.20	45.12	0.11	26.22	0.17	93.05
85-33	10.10	16.54	0.98	0.23	37.15	0.21	32.63	0.06	97.89
85-34	8.88	14.33	0.71	0.08	45.94	0.12	26.16	0.09	96.31
85-35	8.54	10.35	0.80	0.14	51.00	0.11	26.51	0.12	97.56
85-37	11.66	23.78	0.65	0.20	36.48	0.13	24.63	0.06	97.58
85-38	7.44	7.83	0.43	0.10	55.85	0.12	26.21	0.10	98.08
85-39	6.98	8.67	0.67	0.10	53.63	0.12	26.90	0.11	97.18
85-41	9.73	5.15	0.21	0.06	62.55	0.14	20.49	0.11	98.44
85-42	10.36	15.89	0.73	0.18	43.41	0.16	26.08	0.00	96.81
85-43	8.26	10.35	0.80	0.13	48.51	0.12	27.16	0.06	95.40
85-44	9.52	5.36	0.25	0.06	59.82	0.16	21.93	0.50	97.61
85-45	8.78	5.32	0.27	0.01	59.29	0.18	24.07	0.13	98.05

85-46	8.39	10.98	0.72	0.11	49.34	0.07	27.08	0.06	96.75

+6.06 to +6.21 m

Sample weight 9.9 kg.

EC grains >63 µm

Grain	MgO	Al_2O_3	TiO_2	V_2O_3	Cr_2O_3	MnO	FeO	ZnO	Total
86-5	2.66	5.84	2.50	0.85	58.42	0.62	26.40	0.53	97.82
86-8	2.93	5.44	3.36	0.63	58.69	0.47	28.14	0.26	99.92
86-10	3.76	5.44	2.95	0.72	56.80	0.28	23.24	0.89	94.08
86-21	2.51	5.43	3.09	0.70	58.29	0.50	27.46	0.33	98.31
86-23	2.22	5.63	3.14	0.66	58.31	0.60	27.55	0.33	98.45
86-25	3.76	6.13	2.39	0.76	57.62	0.93	26.89	0.71	99.19
86-26	3.72	5.35	3.10	0.70	57.48	0.42	28.93	0.24	99.92
86-27	2.80	5.55	3.04	0.73	57.41	0.61	29.00	0.36	99.51
86-31	5.41	5.73	2.71	0.71	56.60	0.36	25.09	0.37	96.98
86-33	4.35	5.68	3.22	0.69	57.96	0.44	24.46	0.48	97.27
86-34	1.21	5.32	1.79	0.54	55.17	0.43	29.76	0.94	95.16
86-36	2.33	5.43	2.98	0.57	56.13	0.49	28.39	0.30	96.61
86-40	2.96	4.99	2.35	0.63	58.35	0.20	22.80	0.29	92.56
86-41	2.30	5.61	3.27	0.75	58.88	0.48	25.48	0.45	97.23
86-45	2.55	6.11	2.38	0.69	58.75	0.50	27.54	0.54	99.06
86-46	3.13	5.60	1.72	0.68	58.44	0.19	23.32	0.75	93.83
86-49	2.07	5.69	3.18	0.70	59.75	0.51	24.73	0.59	97.20

OtC-V grains >63 µm

Grain	MgO	Al_2O_3	TiO_2	V_2O_3	Cr_2O_3	MnO	FeO	ZnO	Total
86-18	4.79	5.51	0.43	0.78	62.36	0.61	24.35	0.17	99.00

OtC grains >63 µm

Grain	MgO	Al_2O_3	TiO_2	V_2O_3	Cr_2O_3	MnO	FeO	ZnO	Total
86-1	5.60	9.20	0.26	0.20	56.30	0.11	25.42	0.12	97.22
86-3	5.92	11.62	0.07	0.19	53.70	0.18	24.20	0.20	96.08
86-4	8.37	17.55	0.21	0.17	47.04	0.11	22.04	0.10	95.60
86-6	3.78	8.78	0.94	0.23	41.98	0.21	40.50	0.15	96.57
86-7	6.90	13.76	0.62	0.25	50.09	0.08	23.73	0.12	95.55
86-9	5.35	13.82	0.28	0.19	49.53	0.29	27.86	0.17	97.49
86-11	9.10	16.65	0.28	0.10	46.25	0.15	23.69	0.06	96.27
86-12	6.39	17.12	0.76	0.25	34.80	0.20	36.28	0.11	95.91
86-13	7.71	24.43	0.30	0.19	35.80	0.13	27.27	0.13	95.95
86-14	8.92	24.75	0.23	0.19	37.26	0.09	24.34	0.19	95.97
86-15	7.35	13.86	0.43	0.15	47.65	0.15	25.62	0.09	95.29
86-16	4.33	11.07	0.21	0.17	47.60	0.20	32.32	0.15	96.04
86-17	9.74	11.57	0.23	0.10	54.02	0.20	23.04	0.07	98.96
86-19	11.63	13.20	0.24	0.13	52.84	0.09	18.74	0.05	96.90
86-20	7.51	17.65	0.29	0.13	44.64	0.17	25.45	0.10	95.93
86-22	3.35	4.78	0.34	0.06	26.25	0.32	62.09	0.11	97.30
86-24	7.79	17.07	0.07	0.27	49.52	0.16	21.10	0.28	96.26
86-29	10.80	18.11	0.25	0.07	46.94	0.14	20.89	0.13	97.31

86-30	10.22	27.36	0.49	0.27	28.74	0.12	28.17	0.13	95.50
86-32	6.59	16.73	1.11	0.40	31.64	0.18	40.65	0.08	97.38
86-35	6.81	8.65	0.49	0.14	54.28	0.12	23.86	0.04	94.39
86-37	7.44	17.02	0.12	0.25	48.76	0.18	22.44	0.21	96.42
86-38	4.31	5.53	0.35	0.07	57.71	0.22	27.11	0.10	95.38
86-39	9.36	17.48	0.47	0.09	46.54	0.10	21.42	0.00	95.46
86-42	6.90	9.85	0.59	0.15	52.29	0.12	25.57	0.03	95.49
86-43	10.95	25.34	0.73	0.24	27.09	0.18	33.00	0.12	97.65
86-44	6.54	15.45	0.35	0.20	47.74	0.08	24.39	0.42	95.17
86-47	5.16	14.06	0.08	0.35	45.12	0.40	30.36	0.77	96.29
86-48	6.96	16.55	0.31	0.27	45.93	0.14	25.39	0.05	95.61
86-50	6.54	11.61	0.24	0.17	51.57	0.15	25.62	0.11	96.00
86-51	3.94	11.54	0.22	0.12	45.31	0.17	33.74	0.21	95.25
86-2	7.35	14.12	2.08	0.54	28.68	0.24	43.96	0.10	97.07
86-28	6.77	10.74	2.14	0.66	29.95	0.21	47.39	0.12	97.97

+5.90 to +6.06 m

Sample weight 8.6 kg.

EC grains >63 μm

Grain	MgO	Al_2O_3	TiO_2	V_2O_3	Cr_2O_3	MnO	FeO	ZnO	Total
87-7	2.60	5.44	3.51	0.66	60.17	0.42	24.73	0.35	97.88
87-8	2.86	5.70	3.10	0.69	59.24	0.61	27.35	0.54	100.1
87-13	2.15	4.94	3.93	0.79	57.22	0.47	31.14	0.39	101.0
87-14	3.01	5.86	2.84	0.71	57.48	0.63	27.99	0.45	98.96
87-15	2.77	5.58	3.29	0.71	60.07	0.45	25.53	0.33	98.73
87-18	2.65	5.46	3.15	0.66	58.55	0.60	27.04	0.48	98.58
87-22	3.03	8.18	1.99	0.61	56.52	0.46	24.25	0.09	95.13
87-23	2.38	5.57	3.17	0.74	60.31	0.50	25.99	0.41	99.07
87-26	3.14	5.67	2.64	0.72	60.19	0.78	27.07	0.56	100.8
87-31	2.36	5.49	3.30	0.73	58.46	0.59	29.43	0.32	100.7
87-40	1.86	5.64	3.15	0.46	61.99	0.35	24.17	0.22	97.84
87-44	2.13	5.90	3.29	0.68	60.98	0.40	21.31	1.04	95.72
87-55	2.56	4.95	2.31	0.59	57.89	0.44	30.42	0.19	99.34

OtC grains >63 μm

Grain	MgO	Al_2O_3	TiO_2	V_2O_3	Cr_2O_3	MnO	FeO	ZnO	Total
87-1	10.41	12.01	0.35	0.10	54.00	0.06	21.67	0.05	98.66
87-2	9.54	13.26	0.13	0.19	58.75	0.14	15.10	1.03	98.13
87-3	9.75	20.98	0.12	0.25	44.07	0.14	23.26	0.15	98.72
87-4	19.74	14.59	0.22	0.14	40.96	0.36	21.28	0.09	97.39
87-5	6.50	7.81	0.18	0.05	60.39	0.29	22.52	0.67	98.42
87-6	8.72	16.15	0.54	0.21	47.09	0.07	23.45	0.08	96.30
87-9	3.88	17.97	0.16	0.17	40.21	0.10	34.46	0.14	97.09
87-10	3.56	4.93	0.16	0.20	36.28	0.42	52.68	0.16	98.39
87-11	6.02	10.06	0.25	0.11	55.60	0.14	24.41	0.08	96.67
87-12	4.57	9.95	0.18	0.16	50.11	0.27	32.44	0.30	97.98
87-16	6.45	8.49	0.12	0.12	61.27	0.19	20.32	0.21	97.17
87-17	8.29	19.54	1.11	0.13	43.37	0.11	23.42	0.08	96.04
87-19	3.42	4.71	3.19	0.31	18.95	0.29	62.86	0.08	93.81
87-20	9.02	14.53	0.11	0.15	51.73	0.18	22.60	0.20	98.53

87-21	5.93	7.94	0.29	0.06	56.47	0.12	26.09	0.31	97.19
87-24	8.02	18.38	0.93	0.32	32.29	0.22	37.89	0.12	98.17
87-25	9.60	20.03	0.54	0.22	42.57	0.09	23.61	0.08	96.74
87-27	4.41	10.26	0.72	0.26	47.43	0.17	33.70	0.11	97.06
87-28	7.41	11.53	0.42	0.14	53.49	0.12	23.22	0.15	96.49
87-29	7.86	16.85	0.19	0.21	47.16	0.22	25.89	0.18	98.56
87-30	7.51	7.36	0.38	0.10	61.49	0.06	17.19	1.11	95.19
87-32	7.98	21.40	0.60	0.31	36.37	0.15	29.08	0.14	96.02
87-33	7.12	9.01	0.99	0.18	48.76	0.17	30.45	0.06	96.74
87-34	2.96	21.52	0.84	0.32	34.87	0.17	34.08	0.14	94.89
87-35	4.74	8.87	0.07	0.23	62.96	0.23	17.13	1.45	95.67
87-36	12.58	18.64	0.13	0.09	52.20	0.03	13.53	0.03	97.23
87-37	6.07	10.36	0.11	0.15	57.43	0.15	22.50	0.17	96.94
87-38	7.16	10.74	0.90	0.21	53.53	0.13	25.51	0.19	98.38
87-39	7.00	20.13	0.07	0.27	48.69	0.11	19.90	0.17	96.36
87-41	5.60	12.76	0.07	0.17	52.60	0.20	25.39	0.23	97.01
87-42	8.51	14.13	0.76	0.21	43.79	0.13	28.43	0.14	96.08
87-43	4.98	15.06	1.50	0.35	29.14	0.29	46.15	0.13	97.58
87-45	4.97	13.63	0.43	0.09	49.96	0.23	26.75	0.17	96.22
87-46	6.45	9.22	0.09	0.14	56.36	0.26	26.52	0.21	99.25
87-47	7.41	15.50	0.12	0.08	53.23	0.11	18.46	0.18	95.09
87-48	5.20	12.43	0.22	0.24	55.28	0.09	21.59	0.51	95.55
87-49	10.58	29.53	0.56	0.22	37.25	0.07	18.74	0.13	97.08
87-50	10.34	13.81	0.15	0.14	55.05	0.19	18.65	0.53	98.84
87-51	8.33	17.42	0.58	0.21	42.79	0.13	27.31	0.11	96.89
87-52	10.32	12.16	3.35	0.18	39.02	0.19	33.81	0.07	99.11
87-53	8.45	13.85	0.27	0.18	51.72	0.12	22.25	0.07	96.92
87-54	9.00	25.21	1.75	0.27	29.92	0.19	30.89	0.14	97.38
87-56	7.76	8.01	0.30	0.14	58.10	0.14	24.04	0.11	98.60

+5.76 to +5.90 m

Sample weight 8.3 kg.

EC grains >63 μm

Grain	MgO	Al_2O_3	TiO_2	V_2O_3	Cr_2O_3	MnO	FeO	ZnO	Total
88-1	2.70	5.67	2.72	0.67	59.64	0.52	27.05	0.79	99.76
88-5	2.62	5.34	3.04	0.64	58.90	0.49	29.09	0.31	100.4
88-6	4.27	5.50	3.17	0.69	58.76	0.48	25.60	0.52	99.00
88-11	3.03	5.88	3.15	0.75	59.07	0.55	27.75	0.31	100.5
88-13	2.45	5.47	3.12	0.76	58.69	0.54	29.38	0.32	100.7
88-14	2.92	5.43	3.15	0.60	58.62	0.63	29.10	0.33	100.8
88-17	2.80	5.41	3.02	0.61	58.31	0.59	28.79	0.49	100.0
88-19	3.42	5.13	3.17	0.65	58.66	0.68	28.22	0.75	100.7
88-21	2.05	5.49	2.68	0.61	58.18	0.36	25.36	1.28	96.02
88-22	2.06	4.42	1.93	0.59	53.77	0.37	33.94	0.35	97.43
88-25	3.47	5.07	3.04	0.70	57.21	0.70	28.27	0.51	98.97
88-26	1.85	5.60	2.23	0.63	56.72	0.35	27.83	0.79	96.00
88-27	2.86	5.67	3.14	0.60	57.88	0.64	29.32	0.41	100.5

OtC-V grains >63 μm

Grain	MgO	Al_2O_3	TiO_2	V_2O_3	Cr_2O_3	MnO	FeO	ZnO	Total

88-12	5.32	10.72	0.92	0.54	56.26	0.44	25.14	0.12	99.47

OtC grains >63 μm

Grain	MgO	Al_2O_3	TiO_2	V_2O_3	Cr_2O_3	MnO	FeO	ZnO	Total
88-2	12.28	27.72	0.04	0.20	40.15	0.10	17.55	0.24	98.28
88-3	9.34	11.20	0.11	0.17	58.77	0.25	19.87	0.65	100.4
88-4	8.99	26.93	0.12	0.20	36.95	0.19	23.87	0.17	97.41
88-7	7.65	11.72	0.18	0.20	53.50	0.25	26.03	0.15	99.67
88-8	7.67	17.47	0.23	0.11	46.51	0.15	24.54	0.19	96.88
88-9	6.90	9.96	0.10	0.26	60.10	0.22	17.76	0.59	95.87
88-10	9.00	13.54	0.20	0.27	53.15	0.21	23.04	0.17	99.59
88-15	7.55	19.67	2.20	0.40	31.14	0.25	37.05	0.10	98.34
88-16	5.01	13.96	0.19	0.18	47.47	0.16	30.20	0.16	97.33
88-18	7.87	15.72	0.14	0.18	51.37	0.19	21.94	0.21	97.61
88-20	10.24	19.41	0.86	0.16	34.34	0.17	32.62	0.12	97.92
88-24	10.65	21.01	0.84	0.29	32.20	0.18	33.01	0.12	98.32

+5.59 to +5.76 m

Sample weight 10.9 kg.

EC grains >63 μm

Grain	MgO	Al_2O_3	TiO_2	V_2O_3	Cr_2O_3	MnO	FeO	ZnO	Total
89-5	2.76	5.35	3.19	0.63	57.58	0.50	29.18	0.63	99.82
89-6	1.82	5.75	3.17	0.69	57.54	0.57	24.33	1.75	95.62
89-7	5.10	5.93	3.10	0.68	57.40	0.35	23.90	0.38	96.84
89-9	2.41	5.71	3.06	0.67	56.79	0.52	27.78	0.21	97.15
89-10	1.24	6.64	2.34	0.56	55.90	0.37	22.90	0.87	90.82
89-11	2.42	5.89	2.91	0.64	56.51	0.52	26.64	0.46	96.01
89-13	2.51	5.25	2.85	0.64	56.61	0.57	29.73	0.51	98.68
89-14	2.86	5.48	2.93	0.61	56.96	0.64	28.90	0.54	98.93
89-16	2.85	5.16	3.18	0.69	57.52	0.52	27.84	0.30	98.06
89-18	3.26	5.52	2.87	0.60	57.78	0.55	27.87	0.38	98.81
89-19	2.41	5.58	2.85	0.67	57.04	0.61	29.32	0.43	98.91
89-4	9.32	6.21	2.55	0.74	59.37	0.27	19.34	0.27	98.05

OtC grains >63 μm

Grain	MgO	Al_2O_3	TiO_2	V_2O_3	Cr_2O_3	MnO	FeO	ZnO	Total
89-1	7.01	19.18	0.12	0.17	47.83	0.14	22.06	0.26	96.77
89-2	5.19	10.25	0.20	0.16	51.45	0.25	29.36	0.19	97.04
89-3	5.43	17.03	0.23	0.18	45.64	0.19	27.94	0.20	96.85
89-8	8.77	15.49	0.16	0.19	49.74	0.23	24.20	0.15	98.94
89-12	8.55	12.56	0.08	0.21	55.01	0.17	20.08	0.23	96.89
89-15	11.04	22.76	0.10	0.18	42.33	0.11	20.69	0.18	97.38
89-17	7.12	9.02	0.14	0.11	58.42	0.19	21.66	0.16	96.83
89-20	10.21	25.01	0.15	0.15	40.25	0.13	20.32	0.20	96.42

+5.43 to +5.59 m

Sample weight 9.9 kg.

EC grains >63 μm

Grain	MgO	Al_2O_3	TiO_2	V_2O_3	Cr_2O_3	MnO	FeO	ZnO	Total
90-1	5.16	5.43	3.11	0.70	59.85	0.32	22.80	0.28	97.65
90-3	2.33	5.41	3.08	0.59	57.15	0.53	29.03	0.34	98.48
90-8	2.21	5.78	2.77	0.69	58.62	0.48	22.55	2.05	95.15
90-9	2.27	5.74	2.33	0.67	58.06	0.39	25.59	0.50	95.53
90-17	2.96	5.18	3.37	0.69	56.41	0.59	29.05	0.39	98.64
90-18	2.12	5.23	3.14	0.68	56.38	0.48	29.62	0.20	97.84
90-20	1.98	5.45	1.85	0.62	55.48	0.48	22.44	1.53	89.82
90-26	3.19	5.52	2.79	0.70	58.24	0.74	29.09	0.52	100.8
90-28	4.21	5.71	3.23	0.66	62.14	0.35	21.16	0.60	98.10
90-30	2.43	5.41	2.89	0.68	58.10	0.50	27.14	0.75	97.90
90-33	3.09	5.80	3.15	0.68	59.08	0.44	26.78	0.31	99.34
90-38	4.06	5.54	3.33	0.72	61.12	0.25	21.54	0.20	96.78
90-43	4.00	5.27	3.08	0.68	58.91	0.62	26.36	0.48	99.40
90-46	3.58	5.74	3.18	0.71	58.23	0.54	26.83	0.45	99.27
90-47	2.29	5.21	3.02	0.72	58.88	0.51	27.94	0.29	98.85
90-59	2.99	5.16	3.20	0.67	58.41	0.48	28.48	0.57	99.98
90-60	1.39	6.45	1.23	0.54	55.10	0.43	17.39	1.57	84.13
90-65	2.40	5.33	3.13	0.68	59.55	0.54	25.64	0.40	97.71
90-66	2.77	5.41	3.19	0.73	60.26	0.60	24.21	0.50	97.69
90-68	2.60	5.53	3.19	0.55	58.60	0.61	28.35	0.47	99.90
90-69	2.59	5.41	2.95	0.74	57.35	0.49	26.63	0.64	96.84
90-70	1.67	6.19	2.67	0.62	55.70	0.53	23.36	2.18	92.91
90-72	4.86	5.45	2.83	0.56	61.73	0.23	21.79	0.26	97.76
90-78	3.35	5.50	3.09	0.70	57.87	0.70	28.21	0.82	100.3

OtC grains >63 μm

Grain	MgO	Al_2O_3	TiO_2	V_2O_3	Cr_2O_3	MnO	FeO	ZnO	Total
90-2	3.53	13.19	1.35	0.23	41.51	0.41	33.99	0.37	94.57
90-4	5.13	14.09	0.24	0.12	49.87	0.13	25.98	0.14	95.70
90-5	8.46	13.83	0.49	0.18	48.17	0.09	24.64	0.06	95.92
90-6	9.53	13.93	0.49	0.14	48.38	0.09	22.79	0.04	95.39
90-7	8.19	11.87	0.38	0.15	52.21	0.10	22.45	0.07	95.43
90-10	4.21	16.91	0.76	0.20	39.42	0.28	33.59	0.19	95.57
90-11	9.06	14.59	0.48	0.24	45.13	0.09	25.65	0.12	95.36
90-12	9.70	16.07	0.53	0.23	44.65	0.13	24.53	0.16	96.00
90-13	8.79	31.10	0.21	0.22	29.82	0.15	25.70	0.23	96.23
90-14	9.16	13.66	0.46	0.18	48.35	0.11	23.67	0.06	95.65
90-15	8.53	14.15	0.16	0.17	49.28	0.24	25.23	0.13	97.89
90-16	9.24	13.69	0.42	0.14	48.82	0.11	23.22	0.07	95.69
90-19	3.23	13.22	2.64	0.38	31.33	0.65	44.10	1.16	96.71
90-21	6.57	8.73	0.13	0.23	55.56	0.30	27.51	0.20	99.24
90-23	4.77	9.38	2.59	0.33	29.25	0.43	51.20	0.43	98.39
90-22	9.28	17.25	0.52	0.21	42.37	0.12	26.60	0.06	96.40
90-24	9.45	15.08	0.49	0.17	46.33	0.13	24.20	0.06	95.91
90-25	3.74	12.10	0.33	0.13	50.86	0.36	30.26	0.30	98.09
90-27	9.28	13.37	0.44	0.19	48.52	0.12	24.82	0.11	96.98
90-29	0.18	1.69	3.19	0.34	34.07	0.09	15.13	17.83	72.60
90-31	9.52	14.70	0.51	0.18	47.00	0.10	24.68	0.13	96.91
90-32	8.56	12.61	0.40	0.13	50.76	0.13	24.35	0.08	97.09

90-34	9.36	15.17	0.50	0.21	46.90	0.10	24.46	0.10	96.91
90-35	9.16	13.50	0.42	0.17	50.36	0.13	23.69	0.12	97.64
90-36	9.95	13.36	0.48	0.17	48.92	0.15	24.12	0.06	97.31
90-37	10.58	12.49	0.38	0.16	50.66	0.14	23.51	0.10	98.09
90-39	9.77	13.26	0.47	0.18	48.85	0.11	23.93	0.12	96.83
90-40	9.38	14.87	0.49	0.18	48.16	0.11	23.42	0.02	96.75
90-41	8.87	15.93	0.52	0.18	45.02	0.13	26.09	0.12	96.99
90-42	8.75	13.80	0.43	0.17	50.34	0.06	23.06	0.08	96.83
90-44	7.60	15.91	0.52	0.17	44.94	0.13	27.87	0.07	97.31
90-48	8.24	12.63	0.20	0.09	54.67	0.08	20.24	0.26	96.58
90-49	9.83	12.98	0.45	0.21	50.77	0.11	23.60	0.08	98.10
90-50	9.06	16.18	0.55	0.20	43.65	0.08	27.21	0.08	97.14
90-51	9.97	30.84	0.07	0.11	34.42	0.15	22.06	0.18	97.89
90-52	8.54	13.34	0.47	0.12	48.55	0.12	25.02	0.13	96.38
90-53	9.00	11.54	0.21	0.05	54.92	0.12	22.85	0.10	98.89
90-54	8.48	14.65	1.36	0.30	37.05	0.17	35.75	0.11	98.07
90-55	10.51	14.29	0.50	0.21	48.07	0.11	23.06	0.00	96.87
90-56	8.94	16.88	0.52	0.23	43.80	0.13	25.41	0.08	96.11
90-57	3.06	14.76	0.45	0.24	46.18	0.33	31.51	0.38	96.93
90-58	9.41	13.28	0.46	0.15	49.51	0.11	24.30	0.05	97.42
90-61	5.86	17.10	0.89	0.25	41.19	0.17	31.47	0.13	97.18
90-62	10.32	15.18	0.44	0.19	48.11	0.11	22.84	0.05	97.47
90-63	9.36	18.42	0.58	0.23	41.99	0.12	26.15	0.09	97.04
90-64	3.99	11.94	0.49	0.17	44.21	0.54	34.80	0.44	96.59
90-67	8.54	11.10	0.46	0.13	52.85	0.08	23.65	0.12	97.03
90-71	9.39	15.03	0.50	0.22	47.87	0.14	24.34	0.03	97.68
90-73	5.80	17.57	0.17	0.22	44.53	0.22	28.69	0.31	97.59
90-74	9.79	12.07	0.38	0.17	52.28	0.09	22.17	0.09	97.14
90-75	9.96	15.46	0.54	0.24	46.57	0.11	23.55	0.02	96.60
90-76	11.12	22.86	0.14	0.16	44.08	0.18	20.59	0.16	99.34
90-77	10.54	13.13	0.30	0.09	53.90	0.10	19.71	0.06	97.92
90-79	9.57	14.40	0.46	0.16	48.46	0.09	23.55	0.09	96.94
90-80	11.61	11.10	0.85	0.24	39.80	0.14	34.33	0.06	98.26
90-45	2.41	8.75	2.47	0.61	34.78	0.65	46.85	0.80	97.46

+5.26 to +5.43 m

Sample weight 11.1 kg.

EC grains >63 µm

Grain	MgO	Al_2O_3	TiO_2	V_2O_3	Cr_2O_3	MnO	FeO	ZnO	Total
91-1	2.31	5.92	3.18	0.71	60.11	0.34	24.95	0.20	97.70
91-2	2.80	5.63	3.15	0.73	58.15	0.58	27.04	0.43	98.51
91-4	2.21	5.51	3.20	0.68	59.42	0.47	24.60	0.47	96.55
91-5	2.79	5.67	3.15	0.63	58.09	0.51	25.58	0.46	96.88
91-6	1.72	5.83	3.18	0.69	60.05	0.38	25.91	0.16	97.91
91-9	2.60	5.46	3.05	0.69	57.72	0.55	29.78	0.41	100.2
91-11	2.29	6.01	2.35	0.63	57.39	0.71	24.00	1.84	95.23
91-12	2.18	5.61	2.98	0.72	58.01	0.55	28.11	0.32	98.48
91-14	2.23	5.60	3.04	0.69	56.33	0.53	30.05	0.34	98.80
91-16	1.65	6.09	2.76	0.69	59.49	0.37	23.76	0.31	95.13
91-18	2.64	5.18	3.21	0.73	56.78	0.60	29.52	0.30	98.97
91-19	2.11	5.63	3.17	0.66	58.24	0.58	26.00	1.94	98.34
91-24	2.20	5.69	3.04	0.72	59.54	0.47	24.40	1.06	97.11

91-25	3.67	5.48	3.09	0.68	57.71	0.41	27.89	0.41	99.32
91-26	2.48	5.55	2.75	0.64	57.04	1.03	22.59	5.39	97.47
91-27	2.37	5.18	3.23	0.70	56.92	0.76	29.74	1.05	99.94
91-28	2.58	5.54	2.69	0.63	57.97	0.62	29.93	0.48	100.5
91-3	8.45	6.20	2.26	0.65	59.44	0.26	19.34	0.30	96.88

OtC grains >63 µm

Grain	MgO	Al_2O_3	TiO_2	V_2O_3	Cr_2O_3	MnO	FeO	ZnO	Total
91-7	8.14	12.23	0.16	0.14	54.15	0.19	21.88	0.19	97.09
91-8	10.64	14.12	0.08	0.22	52.75	0.18	19.40	0.24	97.63
91-10	7.10	9.94	0.03	0.28	60.32	0.96	17.30	1.28	97.21
91-13	8.73	25.90	0.20	0.19	36.27	0.16	25.50	0.25	97.18
91-15	3.95	8.68	0.13	0.26	54.31	0.33	28.67	1.03	97.37
91-17	6.80	14.04	0.67	0.20	38.98	0.18	35.97	0.13	96.98
91-20	5.66	10.94	0.21	0.14	51.73	0.22	27.24	0.29	96.44
91-21	8.64	34.46	0.12	0.32	24.12	0.18	29.75	0.49	98.09
91-22	4.82	9.73	0.08	0.23	60.53	0.53	18.75	0.48	95.14
91-23	9.47	11.33	0.05	0.17	58.89	1.09	16.73	1.38	99.11

+5.09 to +5.26 m

Sample weight 9.9 kg.

EC grains >63 µm

Grain	MgO	Al_2O_3	TiO_2	V_2O_3	Cr_2O_3	MnO	FeO	ZnO	Total
92-1	2.44	5.77	2.92	0.68	57.98	0.51	25.91	0.94	97.13
92-2	2.01	5.74	3.15	0.78	59.58	0.46	25.21	0.74	97.66
92-3	2.30	5.40	3.03	0.61	57.92	0.46	28.27	0.38	98.36
92-5	3.40	5.41	3.35	0.72	58.08	0.66	28.23	0.55	100.4
92-8	2.29	6.21	3.01	0.69	59.36	0.48	24.70	0.75	97.49
92-9	2.19	5.67	3.09	0.68	58.27	0.48	27.61	0.33	98.30
92-10	1.85	5.75	2.66	0.71	57.43	0.41	27.65	0.54	97.00
92-11	2.25	5.66	3.22	0.67	58.28	0.46	28.74	0.27	99.55
92-12	3.96	5.80	3.16	0.62	59.28	0.41	24.02	0.79	98.03
92-14	2.30	5.44	3.22	0.81	58.84	0.46	27.43	0.34	98.85
92-17	2.52	5.78	2.03	0.80	60.48	0.63	26.13	0.87	99.24
92-18	2.10	5.76	3.56	0.62	57.67	0.57	26.57	0.39	97.23
92-19	2.21	5.59	2.32	0.70	57.32	0.45	24.51	0.44	93.53

OtC-V grains >63 µm

Grain	MgO	Al_2O_3	TiO_2	V_2O_3	Cr_2O_3	MnO	FeO	ZnO	Total
92-6	5.27	13.12	0.61	0.55	55.07	0.18	23.71	0.07	98.57

OtC grains >63 µm

Grain	MgO	Al_2O_3	TiO_2	V_2O_3	Cr_2O_3	MnO	FeO	ZnO	Total
92-4	3.87	7.43	0.19	0.18	56.00	0.21	28.19	0.24	96.31
92-7	9.57	14.24	0.37	0.15	48.63	0.09	24.27	0.15	97.47

92-13	3.59	8.60	0.30	0.07	55.26	0.16	29.09	0.19	97.25
92-15	9.05	9.58	0.12	0.08	60.45	0.14	17.18	0.26	96.87
92-16	4.97	13.02	0.06	0.21	50.54	0.20	27.15	0.32	96.48

+4.90 to +5.09 m

Sample weight 11.1 kg.

EC grains >63 µm

Grain	MgO	Al_2O_3	TiO_2	V_2O_3	Cr_2O_3	MnO	FeO	ZnO	Total
93-2	2.81	5.37	3.13	0.61	56.91	0.69	29.00	0.60	99.13
93-3	2.10	5.59	3.25	0.72	59.20	0.41	24.89	0.30	96.47
93-4	4.90	5.35	2.70	0.69	56.80	0.42	26.34	0.50	97.69
93-5	4.33	5.30	2.74	0.78	57.62	0.57	27.08	0.80	99.21
93-6	2.23	5.59	3.22	0.66	59.52	0.49	24.57	0.51	96.80
93-7	2.01	5.46	3.13	0.82	58.82	0.56	24.64	0.36	95.79
93-8	2.29	5.40	3.36	0.62	59.79	0.54	25.59	0.61	98.19
93-9	2.97	7.87	2.91	0.58	57.99	0.38	23.69	0.52	96.90
93-10	2.04	5.50	3.04	0.60	58.64	0.48	27.58	0.44	98.31
93-11	2.68	5.60	3.18	0.70	58.45	0.56	27.21	0.70	99.08
93-12	1.82	5.93	3.32	0.74	60.22	0.42	23.44	0.50	96.38
93-13	2.02	5.59	2.93	0.63	58.21	0.47	27.36	0.61	97.82
93-14	1.89	5.61	3.03	0.71	59.81	0.66	25.05	0.86	97.62
93-15	2.01	5.61	3.07	0.67	59.02	0.78	24.52	1.20	96.88
93-16	2.50	5.45	3.20	0.68	58.56	0.48	28.91	0.51	100.3
93-17	1.82	5.03	2.46	0.65	58.06	0.44	26.17	0.63	95.26
93-18	2.79	5.63	2.98	0.72	59.24	0.26	24.12	0.62	96.36
93-21	2.55	5.56	3.30	0.66	58.95	0.51	25.37	0.64	97.53

OtC grains >63 µm

Grain	MgO	Al_2O_3	TiO_2	V_2O_3	Cr_2O_3	MnO	FeO	ZnO	Total
93-1	9.54	14.74	0.23	0.16	50.65	0.15	22.08	0.12	97.65
93-19	6.11	9.06	0.32	0.09	52.75	0.11	27.61	0.15	96.20
93-20	8.73	17.02	0.60	0.21	42.53	0.13	26.95	0.07	96.25

+4.68 to +4.90 m

Sample weight 13.1 kg.

EC grains >63 µm

Grain	MgO	Al_2O_3	TiO_2	V_2O_3	Cr_2O_3	MnO	FeO	ZnO	Total
94-7	3.07	5.43	3.12	0.71	58.38	0.65	29.13	0.51	101.0
94-9	1.96	4.38	2.37	0.68	61.37	0.71	27.43	0.86	99.75
94-11	2.69	5.46	3.15	0.74	58.54	0.63	29.33	0.38	100.9
94-12	2.82	5.26	3.24	0.60	58.53	0.64	28.92	0.42	100.4
94-13	3.04	5.38	3.10	0.78	58.10	0.57	28.94	0.52	100.4
94-18	3.47	5.56	3.23	0.65	59.64	0.45	25.07	0.31	98.37
94-19	2.59	5.48	3.23	0.74	58.70	0.37	27.50	0.34	98.94

94-22	3.03	5.50	3.19	0.70	59.84	0.61	24.31	0.91	98.09
94-23	2.33	5.47	3.17	0.74	58.71	0.55	27.58	0.40	98.95
94-24	2.89	5.67	2.91	0.74	58.05	0.61	29.24	0.57	100.7
94-25	2.49	5.66	3.12	0.68	57.86	0.54	28.74	0.29	99.38
94-26	2.29	6.61	2.70	0.78	58.70	0.48	26.71	0.45	98.72
94-27	2.03	5.62	3.10	0.69	59.14	0.41	27.40	0.30	98.68
94-29	2.20	5.34	2.79	0.65	57.40	0.49	31.28	0.21	100.4
94-30	2.65	5.47	3.16	0.72	57.21	0.61	29.59	0.39	99.79
94-32	3.72	5.39	3.20	0.76	59.28	0.60	28.13	0.23	101.3
94-33	1.62	5.92	3.07	0.66	60.23	0.62	26.17	1.69	99.97
94-35	2.07	5.88	3.14	0.73	60.09	0.47	25.64	0.30	98.32
94-36	2.65	5.56	2.92	0.79	60.42	0.59	25.92	0.43	99.27
94-37	2.39	5.52	3.09	0.73	58.43	0.61	29.94	0.47	101.2
94-39	2.06	5.37	2.94	0.69	57.70	0.44	29.10	0.73	99.02

OtC-V grains >63 µm

Grain	MgO	Al_2O_3	TiO_2	V_2O_3	Cr_2O_3	MnO	FeO	ZnO	Total
94-2	4.09	11.74	1.13	0.63	57.65	0.33	23.22	0.07	98.87

OtC grains >63 µm

Grain	MgO	Al_2O_3	TiO_2	V_2O_3	Cr_2O_3	MnO	FeO	ZnO	Total
94-3	4.18	14.32	0.46	0.25	45.25	0.28	32.39	0.25	97.39
94-4	4.54	9.50	0.84	0.17	53.82	0.23	28.27	0.28	97.64
94-5	6.47	6.69	0.33	0.05	61.30	0.15	22.59	0.12	97.68
94-6	5.65	10.55	0.18	0.07	56.04	0.09	24.34	0.16	97.08
94-8	4.42	7.55	0.80	0.31	34.24	0.23	50.57	0.07	98.19
94-10	7.21	12.08	0.21	0.05	53.15	0.14	25.21	0.08	98.14
94-14	6.74	5.87	0.08	0.10	60.19	0.19	25.41	0.45	99.03
94-15	12.24	12.40	0.21	0.07	55.57	0.05	18.00	0.10	98.63
94-16	8.90	10.51	0.13	0.08	56.09	0.13	22.62	0.23	98.68
94-17	5.84	8.67	0.24	0.07	57.58	0.09	22.31	0.36	95.15
94-20	7.97	8.96	0.11	0.14	57.41	0.60	21.31	1.11	97.62
94-21	9.92	19.40	1.01	0.17	43.45	0.12	22.00	0.10	96.17
94-28	10.47	20.66	0.30	0.13	44.97	0.10	20.46	0.10	97.17
94-31	12.71	11.47	0.13	0.07	54.25	0.34	17.90	0.18	97.04
94-34	6.69	11.84	0.19	0.15	54.24	0.22	25.51	0.23	99.07
94-38	9.72	16.28	0.04	0.28	54.74	0.16	15.65	0.56	97.42
94-40	8.76	11.01	0.22	0.20	52.51	0.12	24.48	0.09	97.39
94-1	7.07	13.67	3.27	0.49	17.24	0.27	55.02	0.07	97.09

+4.47 to +4.68 m

Sample weight 14.2 kg.

EC grains >63 µm

Grain	MgO	Al_2O_3	TiO_2	V_2O_3	Cr_2O_3	MnO	FeO	ZnO	Total
95-2	2.22	5.13	3.60	0.81	56.85	0.45	30.73	0.19	99.99
95-4	2.16	6.36	3.05	0.62	60.39	0.45	25.94	0.11	99.09
95-5	6.09	5.36	2.64	0.68	58.22	0.32	25.57	0.16	99.03
95-6	5.49	5.32	2.50	0.74	60.07	0.39	23.80	1.06	99.37

95-8	1.94	5.49	3.34	0.68	58.78	0.33	28.68	0.25	99.49
95-11	2.51	5.91	3.44	0.79	58.91	0.49	26.75	0.34	99.14
95-12	2.11	5.76	3.22	0.61	58.81	0.43	25.82	0.96	97.73
95-14	2.27	5.69	3.27	0.68	58.01	0.56	29.31	0.38	100.2
95-16	2.49	5.80	2.78	0.64	59.42	0.51	26.12	1.22	98.97
95-17	1.95	5.81	2.67	0.72	59.55	0.38	24.96	0.38	96.42
95-18	1.86	5.41	3.29	0.70	59.60	0.46	27.28	0.63	99.22
95-19	2.46	5.49	3.24	0.70	58.67	0.49	27.63	0.34	99.03
95-23	2.88	5.58	3.13	0.67	58.34	0.54	28.94	0.41	100.5
95-24	1.90	5.70	2.91	0.64	58.46	0.44	29.19	0.35	99.58
95-26	2.36	5.46	3.13	0.72	57.92	0.60	29.75	0.34	100.3
95-27	2.35	5.63	3.25	0.79	59.77	0.52	25.90	0.34	98.54
95-29	2.24	5.56	3.39	0.70	60.27	0.31	25.24	0.38	98.08
95-32	2.16	5.65	2.98	0.70	59.01	0.57	28.53	0.65	100.3
95-34	2.33	5.60	3.48	0.65	60.55	0.31	23.27	0.23	96.42
95-35	2.64	5.32	3.31	0.82	59.26	0.42	26.51	0.73	99.02
95-36	2.31	5.66	3.08	0.71	59.03	0.51	28.95	0.42	100.7
95-37	1.32	6.14	2.92	0.81	61.92	0.51	22.39	0.46	96.48
95-38	2.93	5.87	3.22	0.78	59.76	0.40	25.17	0.34	98.48
95-39	2.44	5.45	2.96	0.66	59.26	0.47	28.77	0.23	100.2
95-40	2.54	5.91	3.07	0.68	58.63	0.35	26.75	0.19	98.12
95-42	2.15	5.80	3.07	0.70	59.89	0.40	27.94	0.37	100.3
95-43	3.59	6.73	2.22	0.73	59.23	0.81	25.28	0.94	99.53
95-45	2.47	5.57	3.14	0.79	59.36	0.63	27.01	0.45	99.42
95-46	2.60	5.66	2.14	0.78	60.29	0.46	24.78	0.81	97.51
95-47	5.46	5.25	2.48	0.62	60.45	0.45	23.27	0.72	98.70
95-48	2.12	5.56	3.50	0.72	59.46	0.36	23.86	0.28	95.86
95-49	2.44	5.39	3.09	0.72	58.11	0.48	29.16	0.41	99.79
95-50	2.03	5.60	2.97	0.67	58.44	0.41	30.02	0.31	100.5
95-51	1.52	6.25	3.21	0.77	60.01	0.27	25.02	0.37	97.41
95-52	1.90	5.76	2.99	0.67	59.02	0.42	26.81	0.40	97.97
95-53	1.07	4.81	2.23	0.49	43.94	0.20	20.79	1.16	74.69
95-54	1.90	5.59	3.32	0.62	60.23	0.28	25.15	0.22	97.32
95-55	2.26	5.33	3.18	0.62	57.81	0.71	29.86	0.78	100.6
95-56	1.00	5.54	2.90	0.65	55.42	0.27	24.39	3.73	93.90
95-57	2.05	5.51	3.14	0.75	58.13	0.52	30.63	0.31	101.0
95-58	2.39	5.98	3.14	0.62	60.92	0.44	23.03	0.36	96.89
95-59	2.71	5.67	2.91	0.75	58.47	0.59	28.20	0.56	99.86
95-61	1.23	6.47	3.07	0.83	62.42	0.29	21.90	0.30	96.50
95-62	2.61	5.62	2.91	0.61	59.02	0.59	29.31	0.29	101.0
95-64	2.01	5.91	2.84	0.69	58.56	0.40	28.23	0.36	99.01
95-66	1.94	6.45	3.21	0.71	57.89	0.38	26.97	1.38	98.92

OtC grains >63 μm

Grain	MgO	Al_2O_3	TiO_2	V_2O_3	Cr_2O_3	MnO	FeO	ZnO	Total
95-1	5.84	6.99	0.13	0.20	59.75	0.24	27.26	0.16	100.6
95-3	4.49	16.34	0.16	0.25	43.75	0.19	30.30	0.36	95.85
95-7	8.27	22.98	1.69	0.19	27.31	0.20	37.03	0.13	97.82
95-9	11.36	13.06	0.13	0.12	56.87	0.22	17.03	0.53	99.32
95-10	8.00	13.91	2.30	0.30	33.01	0.22	40.55	0.11	98.39
95-13	11.24	20.86	0.04	0.22	46.32	0.09	17.94	0.14	96.85
95-15	12.03	23.86	1.11	0.24	32.57	0.15	28.05	0.10	98.11
95-20	12.28	26.36	1.05	0.22	30.49	0.15	27.69	0.09	98.34
95-21	8.84	21.11	0.08	0.21	45.12	0.16	22.26	0.27	98.04
95-22	8.43	13.29	0.25	0.13	55.01	0.05	18.66	0.18	95.99
95-25	4.58	19.05	0.36	0.24	41.65	0.35	30.03	0.32	96.57

95-28	11.75	23.48	1.23	0.28	31.80	0.19	29.54	0.06	98.33
95-30	7.17	32.85	0.90	0.13	34.23	0.24	22.08	0.23	97.83
95-31	11.69	23.48	1.22	0.26	31.68	0.18	29.38	0.12	98.00
95-33	6.37	19.04	0.13	0.27	42.08	0.18	27.39	0.24	95.69
95-41	1.94	2.11	0.90	0.21	53.37	0.72	38.80	0.36	98.41
95-44	7.61	13.16	2.51	0.33	32.63	0.22	41.47	0.15	98.07
95-60	6.91	9.38	0.21	0.08	56.76	0.27	26.15	0.17	99.93
95-63	5.72	4.96	0.10	0.15	58.39	0.30	29.88	0.15	99.64
95-65	7.03	13.79	2.55	0.25	29.76	0.24	43.29	0.12	97.03

+4.16 to +4.47 m

Sample weight 12.9 kg.

EC grains >63 µm

Grain	MgO	Al_2O_3	TiO_2	V_2O_3	Cr_2O_3	MnO	FeO	ZnO	Total
96-1	2.28	5.48	3.12	0.77	59.73	0.46	26.25	0.43	98.52
96-2	2.92	10.16	3.32	0.64	53.64	0.11	24.81	0.30	95.90
96-7	1.75	5.73	3.32	0.71	61.55	0.34	23.89	0.23	97.50
96-8	2.51	5.40	3.17	0.81	58.18	0.54	28.45	0.47	99.52
96-9	1.61	5.62	3.21	0.68	60.94	0.27	23.87	0.24	96.44
96-12	2.00	5.95	3.20	0.74	59.75	0.51	26.11	0.40	98.66
96-13	2.69	5.82	3.26	0.65	60.79	0.37	24.66	0.36	98.60
96-21	2.57	5.64	3.18	0.72	58.56	0.62	28.51	0.39	100.2
96-22	2.28	5.59	3.18	0.63	59.87	0.46	25.80	0.28	98.08
96-26	1.74	5.44	3.12	0.78	57.79	0.41	27.55	1.01	97.84
96-30	2.11	5.80	3.20	0.74	59.82	0.47	25.63	0.82	98.58
96-31	2.05	4.96	2.88	0.56	56.69	0.45	27.11	0.41	95.10
96-33	1.68	6.18	3.09	0.63	59.25	0.41	20.36	4.02	95.62
96-38	2.24	5.41	3.21	0.69	60.41	0.44	25.72	0.39	98.50
96-47	1.59	5.34	2.76	0.72	58.25	0.34	28.53	0.27	97.81
96-48	2.27	5.50	3.17	0.75	59.66	0.40	26.81	0.57	99.13
96-60	2.37	5.85	2.87	0.87	57.59	0.64	28.99	0.73	99.91
96-71	1.49	5.81	3.22	0.73	61.38	0.39	24.43	0.28	97.74
96-75	1.78	5.90	3.18	0.70	60.72	0.41	24.08	0.35	97.12
96-80	1.92	5.05	2.86	0.65	58.23	0.43	29.88	0.32	99.34
96-81	1.77	5.16	3.41	0.76	62.21	0.50	23.65	0.79	98.25
96-88	2.57	5.42	3.21	0.71	61.71	0.24	21.08	0.64	95.67
96-89	1.67	5.03	2.79	0.51	59.09	0.32	27.60	0.16	97.18
96-91	2.73	5.09	2.48	0.68	59.78	0.33	26.44	0.22	97.77
96-93	4.30	5.56	2.61	0.66	61.33	0.32	21.19	0.52	96.48
96-95	2.63	5.69	2.94	0.64	60.17	0.39	24.59	0.35	97.41
96-103	2.76	5.42	2.94	0.63	60.10	0.31	24.29	0.31	96.77
96-109	3.88	4.24	1.72	0.56	60.85	0.20	25.25	1.06	97.93
96-110	2.16	5.39	3.18	0.73	58.53	0.31	26.10	1.21	97.61
96-115	2.83	5.28	3.10	0.70	58.41	0.48	28.35	0.37	99.55
96-119	2.16	5.64	3.16	0.67	60.46	0.46	26.26	0.21	99.04
96-120	2.47	5.62	3.15	0.66	60.46	0.50	24.12	0.84	97.83
96-121	2.70	5.22	3.13	0.72	58.52	0.68	29.10	0.79	100.9
96-124	2.54	5.63	3.30	0.82	61.00	0.42	23.68	0.85	98.25
96-132	2.32	6.17	2.86	0.79	60.39	0.55	25.14	0.62	98.86
96-133	3.29	5.42	3.02	0.67	57.89	0.70	28.52	0.57	100.1

OtC grains >63 μm

Grain	MgO	Al_2O_3	TiO_2	V_2O_3	Cr_2O_3	MnO	FeO	ZnO	Total
96-3	4.59	2.75	0.23	0.16	49.87	0.62	39.87	0.33	98.41
96-4	5.43	3.69	1.32	0.12	39.55	0.60	47.44	0.26	98.40
96-5	4.24	2.60	0.75	0.20	40.31	0.72	50.00	0.19	99.01
96-6	4.30	1.55	2.87	0.26	29.39	0.48	58.75	0.08	97.68
96-10	5.27	3.89	1.93	0.17	32.50	0.32	53.73	0.09	97.90
96-11	4.13	1.82	1.40	0.19	33.67	0.68	56.31	0.16	98.36
96-14	4.33	4.93	1.57	0.14	44.25	0.54	42.41	0.27	98.45
96-15	3.76	1.71	3.24	0.15	27.03	0.46	61.04	0.16	97.56
96-16	5.89	4.73	1.14	0.17	42.15	0.34	44.59	0.20	99.21
96-17	1.86	3.42	0.70	0.18	33.74	0.83	57.66	0.19	98.58
96-18	5.73	3.60	1.65	0.17	41.37	0.79	44.36	0.29	97.95
96-19	1.62	1.92	0.71	0.13	36.50	0.77	57.05	0.17	98.86
96-20	3.83	4.81	0.18	0.31	48.61	0.61	38.03	0.32	96.71
96-23	1.26	1.81	0.70	0.15	32.34	0.70	60.47	0.22	97.65
96-24	1.67	2.10	0.66	0.11	35.80	0.81	57.51	0.17	98.82
96-25	5.03	2.28	1.71	0.15	37.16	0.58	51.82	0.19	98.92
96-27	5.60	2.78	2.05	0.30	39.34	0.43	47.87	0.22	98.57
96-28	1.49	1.68	0.84	0.19	25.92	0.67	66.88	0.11	97.78
96-29	3.13	2.28	0.43	0.19	27.99	0.53	62.43	0.10	97.09
96-32	5.32	2.43	1.29	0.29	38.49	0.49	50.72	0.21	99.24
96-34	2.26	1.51	0.98	0.23	20.23	0.73	70.62	0.10	96.64
96-35	4.38	2.15	1.54	0.14	30.78	0.60	57.90	0.14	97.64
96-36	3.77	5.71	1.14	0.11	40.09	0.70	47.61	0.24	99.38
96-37	5.72	4.31	2.31	0.11	32.76	0.38	52.17	0.16	97.92
96-39	1.42	2.25	0.51	0.19	45.72	0.69	47.25	0.30	98.33
96-40	3.72	2.04	0.97	0.11	26.48	0.53	63.61	0.14	97.59
96-41	3.67	2.72	0.51	0.19	39.76	0.64	50.59	0.17	98.25
96-42	1.36	1.64	1.15	0.22	22.22	0.66	70.63	0.10	97.97
96-43	2.94	2.38	0.32	0.16	37.02	0.65	55.23	0.34	99.04
96-44	5.15	2.60	1.92	0.22	33.85	0.45	53.88	0.28	98.33
96-45	5.67	2.13	0.43	0.15	41.62	0.73	47.55	0.22	98.49
96-46	1.61	2.04	0.48	0.06	37.27	0.50	56.85	0.44	99.26
96-49	3.64	1.74	2.14	0.18	28.59	0.57	60.96	0.15	97.96
96-50	1.59	2.25	0.64	0.15	34.84	0.79	58.26	0.25	98.77
96-51	4.06	3.29	1.94	0.14	31.44	0.48	56.29	0.13	97.77
96-52	3.76	2.10	0.79	0.16	33.96	0.67	57.04	0.17	98.65
96-53	5.59	2.94	1.58	0.17	40.44	0.44	47.15	0.20	98.52
96-54	4.58	2.90	0.54	0.14	34.47	0.56	54.50	0.14	97.83
96-55	3.84	1.75	0.72	0.10	37.02	0.67	54.19	0.18	98.48
96-56	4.38	6.85	0.05	0.22	55.60	0.20	30.33	0.28	97.90
96-57	5.11	2.40	2.07	0.23	33.24	0.45	55.07	0.18	98.77
96-58	1.50	4.95	0.44	0.09	49.27	0.62	39.36	0.37	96.61
96-59	4.53	5.02	1.09	0.15	29.09	0.46	56.44	0.35	97.13
96-61	4.72	2.45	1.95	0.23	33.79	0.49	54.62	0.12	98.47
96-62	3.87	1.73	0.39	0.17	32.76	0.58	58.35	0.18	98.14
96-63	3.06	1.40	1.76	0.21	26.89	0.53	63.95	0.10	98.02
96-64	4.32	3.05	1.51	0.15	36.94	0.66	52.18	0.17	99.10
96-65	3.54	2.25	1.16	0.19	36.49	0.66	54.64	0.12	99.21
96-66	4.09	2.68	0.52	0.13	45.59	0.50	44.52	0.41	98.52
96-67	5.08	2.79	1.47	0.19	38.49	0.45	49.08	0.19	97.86
96-68	2.16	6.06	0.80	0.16	33.91	1.04	54.15	0.28	98.85
96-69	9.24	28.85	0.94	0.26	25.02	0.15	30.90	0.10	95.50
96-70	5.24	3.12	1.41	0.25	40.65	0.66	46.81	0.21	98.48
96-72	4.21	3.12	2.53	0.08	27.52	0.42	59.71	0.13	97.83

96-73	3.45	1.78	1.12	0.15	25.32	0.60	63.98	0.13	96.65
96-74	3.86	2.69	0.98	0.21	37.82	1.17	51.36	0.19	98.36
96-76	5.72	3.73	1.27	0.17	40.98	0.44	45.87	0.16	98.44
96-77	2.96	1.58	0.64	0.08	29.89	0.65	61.67	0.21	97.84
96-79	5.19	4.18	1.74	0.16	41.98	0.58	44.79	0.23	98.95
96-82	4.59	3.37	1.53	0.16	45.70	0.27	41.60	0.41	97.74
96-83	3.85	1.45	2.20	0.30	22.76	0.48	66.11	0.11	97.43
96-84	1.78	7.17	1.24	0.18	47.65	0.65	37.83	0.41	96.93
96-85	4.50	3.03	1.40	0.22	37.24	0.62	51.72	0.17	99.01
96-86	2.20	0.90	0.78	0.16	13.98	0.33	74.75	0.08	93.18
96-87	5.64	3.05	1.30	0.24	40.16	0.43	47.76	0.30	99.01
96-90	5.26	2.79	2.75	0.24	35.50	0.55	51.34	0.21	98.80
96-92	5.24	3.09	1.72	0.23	37.12	0.39	50.97	0.13	99.00
96-94	3.33	1.21	1.41	0.24	18.68	0.51	71.62	0.08	97.21
96-96	5.40	2.87	1.18	0.18	42.58	0.60	46.27	0.18	99.39
96-97	5.16	2.43	3.11	0.22	31.77	0.43	55.11	0.15	98.53
96-98	4.22	2.51	2.08	0.20	31.30	0.55	56.89	0.18	98.10
96-99	4.37	2.11	0.77	0.09	36.53	0.50	53.67	0.17	98.40
96-100	5.31	6.12	0.82	0.10	40.14	0.45	45.18	0.22	98.44
96-101	3.87	2.14	0.89	0.15	33.59	0.62	57.08	0.20	98.68
96-102	4.91	2.10	2.29	0.30	35.50	0.56	52.68	0.25	98.72
96-104	4.47	2.51	0.86	0.16	34.05	0.55	55.51	0.17	98.43
96-105	4.51	2.79	0.58	0.16	39.76	0.67	49.78	0.18	98.53
96-106	3.51	2.24	1.48	0.30	40.50	0.68	49.21	0.20	98.20
96-107	4.30	2.27	1.58	0.20	31.91	0.55	57.00	0.11	98.05
96-108	4.98	2.39	1.28	0.12	38.69	0.63	50.11	0.24	98.55
96-111	5.79	4.15	0.94	0.13	45.17	0.33	41.15	0.16	97.91
96-112	3.47	1.80	1.11	0.20	29.94	0.47	60.63	0.16	97.93
96-113	4.57	2.54	0.71	0.05	39.92	0.64	49.89	0.16	98.57
96-114	5.51	2.76	1.15	0.18	38.87	0.38	49.81	0.13	98.92
96-116	4.91	4.85	1.30	0.13	42.90	0.70	43.63	0.23	98.77
96-117	6.00	3.10	2.20	0.26	39.11	0.46	47.60	0.17	99.03
96-118	5.07	2.70	1.03	0.13	39.47	0.58	49.67	0.20	98.96
96-122	1.39	1.96	0.65	0.15	36.42	0.67	57.00	0.24	98.50
96-123	5.08	2.98	1.37	0.21	37.88	0.45	50.13	0.16	98.42
96-125	3.64	1.91	1.27	0.14	33.15	0.65	57.14	0.14	98.16
96-126	3.15	5.40	1.47	0.12	45.16	0.28	41.57	0.37	97.59
96-127	4.40	3.54	1.55	0.09	45.24	0.55	42.58	0.39	98.46
96-129	5.52	3.83	1.41	0.15	40.19	0.67	46.91	0.27	99.05
96-131	2.99	6.50	0.86	0.13	44.27	0.30	42.25	0.13	97.46
96-134	5.05	2.18	2.51	0.27	34.18	0.48	54.21	0.21	99.24
96-135	3.65	2.06	3.34	0.21	21.84	0.37	65.23	0.15	97.00
96-136	4.53	2.07	1.13	0.10	36.93	0.63	52.55	0.18	98.25

+3.96 to +4.16 m

Sample weight 12.2 kg.

EC grains >63 µm

Grain	MgO	Al_2O_3	TiO_2	V_2O_3	Cr_2O_3	MnO	FeO	ZnO	Total
97-1	2.70	5.93	3.20	0.74	60.68	0.65	21.47	2.23	97.59
97-2	2.54	5.91	3.23	0.59	59.38	0.49	24.40	0.53	97.07
97-3	1.88	5.73	3.30	0.66	59.48	0.49	25.29	0.59	97.42
97-5	2.32	5.81	3.15	0.75	61.23	0.34	23.55	0.19	97.35

97-6	2.99	5.53	3.19	0.67	58.47	0.50	28.33	0.40	100.1
97-7	2.55	5.55	3.28	0.64	58.13	0.58	29.07	0.29	100.1
97-8	2.47	4.82	3.30	0.70	58.64	0.46	27.69	0.47	98.56
97-9	2.23	5.82	3.04	0.67	59.56	0.38	26.49	0.30	98.48
97-10	2.08	5.82	3.32	0.69	59.98	0.42	24.16	1.18	97.64
97-11	1.96	5.74	2.44	0.56	57.84	0.31	24.74	0.51	94.11
97-12	2.18	5.69	3.17	0.80	59.87	0.52	25.49	0.64	98.35
97-13	3.42	5.41	3.42	0.62	60.25	0.43	25.96	0.31	99.83
97-14	1.91	5.18	2.98	0.65	58.99	0.25	25.69	0.89	96.54
97-15	2.18	5.51	3.19	0.63	59.82	0.41	27.31	0.32	99.36
97-16	2.75	5.73	3.81	0.68	62.13	0.34	21.46	0.48	97.38
97-17	2.43	5.70	3.14	0.65	58.28	0.48	28.82	0.36	99.87
97-18	2.84	5.13	3.56	0.70	58.02	0.56	28.84	0.46	100.1
97-20	2.62	6.25	2.31	0.80	60.65	0.38	23.46	0.44	96.91
97-21	1.80	5.70	2.95	0.60	59.16	0.32	24.89	0.36	95.79
97-22	3.05	5.48	3.50	0.66	60.20	0.38	24.50	0.59	98.34
97-24	1.73	6.16	3.33	0.80	61.65	0.44	21.60	0.23	95.92

OtC-V grains >63 µm

Grain	MgO	Al_2O_3	TiO_2	V_2O_3	Cr_2O_3	MnO	FeO	ZnO	Total
97-4	5.54	9.68	0.88	0.49	59.13	0.44	21.37	0.97	98.50

OtC grains >63 µm

Grain	MgO	Al_2O_3	TiO_2	V_2O_3	Cr_2O_3	MnO	FeO	ZnO	Total
97-19	8.01	9.53	0.02	0.20	59.52	0.18	21.70	0.25	99.41
97-23	9.91	21.47	0.88	0.17	40.03	0.14	23.75	0.10	96.45

+3.76 to +3.96 m

Sample weight 12.2 kg.

EC grains >63 µm

Grain	MgO	Al_2O_3	TiO_2	V_2O_3	Cr_2O_3	MnO	FeO	ZnO	Total
98-1	2.33	5.43	3.05	0.67	58.79	0.62	26.28	0.58	97.74
98-2	2.32	5.29	2.99	0.74	58.35	0.62	27.24	1.04	98.59
98-3	2.26	5.64	3.34	0.63	59.51	0.46	24.65	0.17	96.66
98-4	2.25	5.36	3.29	0.58	57.40	0.65	30.05	0.50	100.1
98-5	2.17	5.51	2.82	0.68	57.25	0.53	28.93	0.37	98.25
98-6	3.13	4.96	2.67	0.56	58.64	0.61	27.72	0.50	98.79
98-7	2.90	5.20	3.09	0.74	57.49	0.63	28.85	0.54	99.42
98-8	2.28	5.36	3.05	0.70	58.76	0.60	28.82	0.42	100.0
98-9	1.52	5.56	1.96	0.50	55.47	0.39	27.87	0.94	94.22
98-10	1.95	5.52	3.15	0.64	58.68	0.43	28.25	0.26	98.86
98-11	2.72	4.98	3.01	0.69	58.48	0.57	28.75	0.74	99.96
98-13	3.53	4.63	2.79	0.63	60.49	1.01	25.40	2.06	100.5
98-14	1.72	5.05	2.96	0.61	58.82	0.42	26.92	1.15	97.66

OtC grains >63 µm

Grain	MgO	Al_2O_3	TiO_2	V_2O_3	Cr_2O_3	MnO	FeO	ZnO	Total
98-12	10.66	12.15	0.14	0.08	55.29	0.12	19.41	0.17	98.01

+3.52 to +3.76 m

Sample weight 15.9 kg.

EC grains >63 μm

Grain	MgO	Al_2O_3	TiO_2	V_2O_3	Cr_2O_3	MnO	FeO	ZnO	Total
99-2	1.91	6.51	2.59	0.68	57.71	0.52	23.81	1.28	95.01
99-3	2.73	5.67	2.89	0.68	57.91	0.44	26.18	0.56	97.06
99-4	2.27	5.79	2.98	0.71	58.04	0.66	29.89	0.71	101.1
99-5	2.92	5.76	2.96	0.73	58.58	0.46	27.93	0.41	99.74
99-6	2.21	6.48	1.91	0.76	61.15	0.64	23.87	0.80	97.81
99-7	2.77	5.59	3.36	0.55	57.08	0.63	29.42	0.67	100.1
99-8	2.61	5.42	3.17	0.69	59.59	0.54	26.26	0.75	99.04
99-9	2.23	5.67	3.11	0.81	58.00	0.58	29.00	0.32	99.71
99-11	6.02	4.98	2.14	0.53	60.18	0.25	21.94	0.32	96.36
99-12	2.61	5.26	1.89	0.46	55.87	0.30	25.19	0.83	92.41
99-13	1.98	5.81	2.65	0.55	59.14	0.44	26.26	0.26	97.10
99-14	2.98	5.44	2.73	0.80	57.63	0.56	29.52	0.51	100.2
99-15	1.94	5.54	3.16	0.69	57.35	1.25	28.81	0.55	99.29
99-16	2.08	5.81	3.15	0.71	61.66	0.38	21.43	0.50	95.71
99-17	3.03	5.87	2.88	0.80	58.87	0.80	26.20	0.64	99.08
99-18	2.17	5.52	3.02	0.73	58.29	0.46	29.05	0.35	99.58
99-19	2.58	5.47	3.19	0.67	58.54	0.51	29.32	0.33	100.6
99-21	2.23	4.86	2.72	0.66	59.46	0.86	28.39	1.05	100.2
99-22	1.99	5.68	3.09	0.76	59.43	0.55	23.95	1.99	97.44
99-23	2.40	5.56	3.12	0.64	59.16	0.43	26.92	0.44	98.68
99-26	2.77	5.71	3.04	0.87	59.07	0.65	26.06	1.60	99.76
99-27	1.58	5.93	2.90	0.81	61.27	0.37	25.53	0.39	98.78
99-28	2.09	4.54	3.65	0.78	58.70	0.38	28.73	0.44	99.32
99-29	2.03	5.94	3.20	0.76	60.17	0.49	24.82	0.44	97.85
99-30	1.86	5.58	2.30	0.57	60.53	0.36	24.66	0.49	96.34
99-31	2.08	5.55	3.32	0.67	60.81	0.50	24.59	0.52	98.03

OtC grains >63 μm

Grain	MgO	Al_2O_3	TiO_2	V_2O_3	Cr_2O_3	MnO	FeO	ZnO	Total
99-1	9.15	15.57	0.10	0.22	49.64	0.16	23.32	0.24	98.41
99-10	8.47	10.98	0.26	0.08	53.17	0.20	24.97	0.07	98.20
99-20	12.31	27.97	0.52	0.21	38.42	0.09	17.18	0.10	96.80
99-25	4.74	11.15	0.06	0.25	51.84	0.19	29.45	0.32	98.00
99-24	6.54	9.82	4.21	0.63	34.55	0.55	42.35	0.14	98.79

+3.27 to +3.52 m

Sample weight 13.6 kg.

EC grains >63 μm

Grain	MgO	Al_2O_3	TiO_2	V_2O_3	Cr_2O_3	MnO	FeO	ZnO	Total
100-1	2.00	5.94	2.51	0.74	56.65	0.40	27.44	0.83	96.50
100-2	2.57	5.30	2.78	0.73	57.25	0.45	28.98	0.37	98.44
100-3	3.33	5.55	2.98	0.73	57.77	0.60	26.84	0.41	98.20
100-5	2.94	6.50	1.94	0.62	59.69	1.36	24.32	1.25	98.62
100-8	2.67	5.84	2.62	0.75	58.45	0.88	28.55	0.64	100.4
100-9	2.39	5.27	2.99	0.71	57.73	0.58	30.58	0.24	100.5
100-13	2.19	5.39	3.21	0.64	58.51	1.48	27.10	0.88	99.39
100-14	2.57	5.58	2.99	0.66	57.87	0.54	29.24	0.45	99.91
100-16	4.74	6.14	2.40	0.67	58.49	0.61	25.93	0.28	99.26
100-17	1.94	5.78	3.04	0.71	58.65	0.38	26.85	0.47	97.83

OtC grains >63 µm

Grain	MgO	Al_2O_3	TiO_2	V_2O_3	Cr_2O_3	MnO	FeO	ZnO	Total
100-4	11.77	22.86	0.07	0.21	45.42	0.07	14.32	0.10	94.80
100-6	11.29	20.94	0.29	0.15	43.53	0.13	21.55	0.10	97.96
100-7	7.02	10.67	0.05	0.21	55.16	0.24	25.66	0.21	99.22
100-10	7.37	10.30	0.11	0.14	54.22	0.23	26.69	0.21	99.26
100-11	8.63	21.83	0.35	0.24	37.67	0.22	29.48	0.19	98.60
100-12	11.15	24.29	0.16	0.17	42.19	0.12	19.71	0.25	98.04
100-15	13.24	22.07	0.04	0.17	46.93	0.07	14.14	0.10	96.75
100-18	1.48	8.02	4.57	0.42	47.96	0.52	33.10	0.09	96.16
100-19	7.51	10.90	0.12	0.20	55.64	0.20	23.97	0.12	98.67

+2.73 to +2.83 m (Sextummen bed)

Sample weight 12.6 kg. From Schmitz and Häggström (2006).

EC grains >63 µm

Grain	MgO	Al_2O_3	TiO_2	V_2O_3	Cr_2O_3	MnO	FeO	ZnO	Total
1	2.01	5.82	3.31	0.81	57.66	0.99	28.88	0.20*	99.68
2	2.60	6.27	3.12	0.76	56.82	0.73	28.43	0.31	99.04
3	2.59	5.88	3.20	0.81	59.73	0.63	26.07	0.49	99.40
4	2.94	6.50	2.59	0.84	57.27	1.13	28.30	0.51	100.1
5	1.92	6.05	3.14	0.88	58.55	0.71	24.77	0.30	96.32
6	2.08	6.48	3.07	0.80	57.59	0.73	27.01	0.20*	97.96
7	2.01	5.92	3.17	0.70	60.08	0.66	23.00	0.43	95.97
8	2.29	4.69	3.29	0.65	56.57	0.84	28.20	0.27	96.80
9	1.86	6.62	3.23	0.77	58.20	0.41	26.81	0.23	98.13
10	2.03	5.38	3.30	0.69	57.52	0.83	28.62	0.30	98.67
11	8.61	5.85	2.96	0.82	58.60	0.63	20.42	0.48	98.37
12	1.28	5.90	3.39	0.80	59.14	0.78	24.25	0.25	95.79
13	2.42	4.61	3.33	0.73	56.33	0.62	27.98	0.33	96.35
14	2.26	8.10	2.95	0.79	59.63	0.50	21.91	0.80	96.94
15	2.45	5.54	3.27	0.74	56.80	0.75	27.53	0.26	97.34
16	1.19	5.33	2.54	0.58	53.77	0.60	31.56	0.03*	95.60
17	1.42	4.98	2.69	0.69	56.33	0.66	27.38	0.38	94.53
18	2.96	6.62	2.42	0.71	54.43	0.76	31.28	0.26	99.44
19	2.48	5.93	3.23	0.65	56.72	0.68	26.64	0.45	96.78
20	3.55	5.61	2.98	0.74	57.20	0.87	27.31	0.31*	98.57
21	2.46	5.52	3.11	0.71	57.30	0.94	23.62	2.13	95.79

*Values lower than the detection limit (<2 sigma).

OtC grains >63 µm

Grain	MgO	Al_2O_3	TiO_2	V_2O_3	Cr_2O_3	MnO	FeO	ZnO	Total
1	9.48	16.48	0.07*	0.26	51.89	0.41	23.42	0.29	102.3
2	7.11	15.12	0.07*	0.19	60.71	0.43	15.60	0.48	99.71
3	11.06	27.52	0.10*	0.11*	40.98	0.31*	17.77	0.19*	98.04
4	9.07	22.29	0.21	0.07*	44.01	0.22*	22.97	0.25*	99.09
5	8.92	12.07	0.01*	0.23	56.42	0.46	18.82	1.89	98.82

*Values lower than the detection limit (<2 sigma).

+2.58 to +2.73 m (Sextummen bed)

Weight 19.0 kg. From Schmitz and Häggström (2006).

EC grains >63 µm

Grain	MgO	Al_2O_3	TiO_2	V_2O_3	Cr_2O_3	MnO	FeO	ZnO	Total
1	1.47	4.72	2.68	0.66	56.74	0.77	29.45	0.70	97.19
2	2.04	7.08	2.65	0.67	53.50	0.69	35.32	0.30	102.3
3	1.47	5.39	2.77	0.59	55.15	0.64	32.02	0.14*	98.17
4	2.63	5.87	3.04	0.76	56.63	0.75	29.42	0.30	99.40
5	1.77	7.15	2.86	0.59	56.84	0.66	31.22	0.16*	101.3
6	1.64	8.15	2.78	0.60	55.26	0.63	29.18	0.15*	98.39
7	2.17	7.11	2.48	0.78	59.25	0.47	25.83	0.42	98.51
8	2.11	7.44	3.08	0.80	59.42	0.92	23.84	0.48	98.09
9	1.57	6.26	3.30	0.65	59.91	0.70	26.02	0.29	98.70
10	3.30	4.80	2.74	0.64	58.52	0.45	26.83	0.23	97.51
11	3.01	6.95	3.02	0.78	58.69	0.63	25.54	0.17*	98.79
12	1.76	5.85	3.30	0.76	58.96	0.51	26.47	1.02	98.63
13	2.92	7.21	3.49	0.82	61.15	0.57	19.08	0.66	95.90
14	1.07	5.28	2.86	0.60	58.11	0.64	29.58	0.13*	98.27
15	0.97	5.25	3.20	0.81	60.13	0.66	25.38	0.43	96.83
16	1.86	6.15	3.10	0.64	55.90	0.60	30.34	0.18*	98.77
17	1.50	7.22	3.43	0.64	60.86	0.56	21.15	0.27	95.63
18	2.72	4.14	2.80	0.68	56.42	0.61	29.97	0.24	97.58
19	2.78	6.42	3.10	0.85	60.47	0.68	21.46	0.16*	95.92
20	1.10	6.33	3.32	0.80	60.76	0.31	22.99	0.19*	95.80
21	4.42	8.49	2.85	0.72	57.50	0.58	25.15	0.27	99.98
22	2.87	7.30	2.98	0.79	57.61	0.57	29.66	0.29	102.1
23	1.02	4.41	2.38	0.73	56.16	0.52	30.34	0.41	95.97
24	2.28	6.69	3.15	0.72	58.60	0.59	23.59	0.24	95.86
25	1.37	6.55	3.16	0.72	60.35	0.55	21.60	0.47	94.77
26	2.11	7.20	3.30	0.77	59.75	0.58	22.25	0.70	96.66

*Values lower than the detection limit (<2 sigma).

OtC-V grains >63 µm

Grain	MgO	Al_2O_3	TiO_2	V_2O_3	Cr_2O_3	MnO	FeO	ZnO	Total
1	2.36	10.91	2.54	0.51	56.90	0.65	24.02	0.20	98.09

OtC grains >63 μm

Grain	MgO	Al_2O_3	TiO_2	V_2O_3	Cr_2O_3	MnO	FeO	ZnO	Total
1	6.65	11.47	0.28	0.09*	59.47	0.36	19.80	0.15*	98.27
2	6.07	9.22	0.14	0.11*	57.59	0.39	23.16	0.17*	96.85
3	10.63	13.31	0.31	0.11*	48.55	0.33	26.81	0.05*	100.1
4	12.95	25.56	0.56	0.16	39.18	0.31	20.13	0.15*	99.00
5	3.90	7.85	0.38	0.18	56.60	0.32	27.65	0.14*	97.02
6	5.06	10.21	0.21	0.21	54.35	0.32	26.28	0.05*	96.69
7	6.73	7.25	0.10*	0.17	62.18	0.21*	17.38	0.43	94.45
8	5.68	11.39	0.17	0.26	52.33	0.44	25.01	0.10*	95.38
9	6.96	7.69	0.12	0.13	58.34	0.43	23.13	0.18*	96.98
10	12.74	11.07	0.22	0.06*	58.08	0.22*	16.09	0.25	98.73
11	5.29	10.21	0.61	0.36	36.44	0.35	43.21	0.14*	96.61
12	5.57	13.83	0.33	0.23	48.15	0.43	25.42	0.13*	94.09
13	4.83	11.01	0.30	0.21	50.39	0.40	26.47	0.07*	93.68
14	7.57	9.22	0.17	0.08*	59.47	0.41	19.72	0.18*	96.82
15	7.93	12.03	0.21	0.11*	53.20	0.41	24.43	0.11*	98.43
16	3.47	9.79	0.12	0.16	55.63	0.26	24.51	0.13*	94.07
17	16.27	12.91	0.12	0.06*	56.44	0.39	16.01	0.23	102.4
18	6.78	8.71	0.36	0.10*	56.68	0.35	23.37	0.12*	96.47
19	9.03	12.62	0.18	0.10*	58.22	1.27	18.50	0.36	100.3
20	10.67	19.27	0.31	0.12*	46.28	0.37	21.85	0.10*	98.97
21	14.07	20.61	0.26	0.17	43.87	0.27	19.50	0.09*	98.84
2	5.31	9.79	1.64	0.98	31.34	0.36	47.23	0.02*	96.67
3	6.46	12.02	1.35	1.20	27.43	0.30	48.95	0.04*	97.75
4	8.83	17.88	1.02	0.60	19.17	0.24*	52.26	0.26*	100.3

*Values lower than the detection limit (<2 sigma).

+2.50 to +2.58 m (Sextummen bed)

Sample weight 10.7 kg. From Schmitz and Häggström (2006).

EC grains >63 μm

Grain	MgO	Al_2O_3	TiO_2	V_2O_3	Cr_2O_3	MnO	FeO	ZnO	Total
1	2.45	7.45	3.61	0.87	63.68	0.92	22.26	0.76	102.0
2	1.21	5.45	3.35	0.85	59.68	0.96	24.32	0.45	96.27
3	2.33	5.89	3.19	0.79	56.86	0.90	28.83	0.75	99.54
4	3.89	7.88	3.28	0.68	55.30	0.66	29.74	0.61	102.0
5	3.91	5.73	3.26	0.72	55.70	0.76	26.57	0.13*	96.78
6	2.09	7.36	3.28	0.56	57.41	0.69	27.30	0.19*	98.88
7	1.81	4.03	3.13	0.69	56.13	1.02	27.53	0.62	94.96
8	1.84	5.45	3.35	0.88	58.28	0.55	24.97	0.33*	95.65
9	1.98	5.94	3.31	0.77	58.01	0.84	24.45	0.53	95.83
10	2.41	6.52	3.50	0.79	55.22	0.82	25.99	0.44	95.69
11	1.38	4.82	2.59	0.59	55.04	0.68	30.53	0.42	96.05
12	1.94	6.05	2.68	0.78	55.75	1.15	28.06	0.81	97.22
13	4.34	6.14	2.85	0.71	56.24	0.89	25.33	0.66	97.16
14	3.42	5.19	3.17	0.72	54.49	0.71	26.21	0.40	94.31
15	1.57	5.11	3.19	0.76	55.60	0.62	26.18	0.47	93.50
16	2.65	5.81	3.08	0.79	56.98	0.92	28.21	0.94	99.38
17	3.83	6.26	2.29	0.74	56.31	0.61	25.00	0.32*	95.36
18	2.46	5.26	3.05	0.69	54.54	0.62	27.14	0.81	94.57

19	1.94	6.72	2.99	0.67	57.76	0.59	22.96	0.39	94.02
20	2.06	6.03	2.05	0.69	55.86	0.80	25.54	0.39	93.42

*Values lower than the detection limit (<2 sigma).

OtC grains >63 µm

Grain	MgO	Al_2O_3	TiO_2	V_2O_3	Cr_2O_3	MnO	FeO	ZnO	Total
1	12.15	18.58	0.69	0.25	38.25	0.47	25.93	0.05*	96.37
2	3.09	11.44	0.33	0.19*	48.84	0.95	29.45	0.56	94.85

*Values lower than the detection limit (<2 sigma).

+2.29 to +2.48 m (Rödkarten bed)

Sample weight 13.0 kg.

EC grains >63 µm

Grain	MgO	Al_2O_3	TiO_2	V_2O_3	Cr_2O_3	MnO	FeO	ZnO	Total
101-1	2.27	5.61	3.05	0.77	58.10	0.99	27.75	0.82	99.35
101-2	2.72	5.37	3.00	0.72	58.20	0.64	28.80	0.73	100.2
101-5	5.11	5.34	2.95	0.64	58.39	0.36	26.43	0.22	99.43
101-6	1.97	5.91	2.58	0.74	58.05	0.68	29.69	0.48	100.1
101-7	2.65	5.51	2.99	0.70	57.44	0.20	30.17	0.22	99.87
101-9	2.31	5.35	3.12	0.65	59.36	0.48	27.27	0.31	98.84
101-10	2.66	5.53	3.00	0.72	57.08	0.64	29.78	0.41	99.82
101-11	3.48	5.31	3.10	0.68	58.01	0.58	28.06	0.41	99.63
101-12	1.98	8.71	1.93	0.64	55.52	0.32	26.00	2.10	97.20
101-13	3.63	5.36	3.01	0.73	59.02	0.49	27.25	0.43	99.92
101-14	1.46	9.42	2.68	0.68	55.61	0.46	19.21	0.54	90.05
101-15	1.83	5.46	2.71	0.70	57.62	0.38	28.17	0.51	97.37
101-16	2.21	5.37	3.01	0.69	57.35	0.82	29.45	0.85	99.75
101-18	2.14	5.54	3.19	0.66	56.69	0.58	30.43	0.37	99.59
101-20	2.73	5.33	3.32	0.64	57.38	0.77	29.26	0.65	100.1
101-21	2.84	5.56	3.18	0.71	57.21	0.58	29.48	0.44	100.0
101-23	2.10	5.90	3.00	0.78	60.57	0.43	23.55	0.60	96.94
101-24	2.41	5.97	2.39	0.72	57.68	0.64	29.05	0.32	99.19
101-25	2.22	5.28	3.13	0.63	55.69	0.50	29.21	0.29	96.94
101-26	2.52	5.58	3.09	0.68	57.47	0.54	29.73	0.31	99.93
101-28	3.77	5.55	3.00	0.75	57.82	0.51	28.57	0.36	100.3
101-29	3.22	5.47	2.38	0.56	58.24	0.41	28.31	0.35	98.94
101-30	2.54	5.69	3.14	0.73	58.49	0.51	28.89	0.34	100.3

OtC grains >63 µm

Grain	MgO	Al_2O_3	TiO_2	V_2O_3	Cr_2O_3	MnO	FeO	ZnO	Total
101-3	6.43	10.09	0.21	0.17	47.02	0.31	33.68	0.18	98.10
101-4	11.67	9.09	0.08	0.06	60.41	0.10	16.47	0.14	98.01
101-8	4.13	12.96	0.29	0.24	46.08	0.22	32.31	0.26	96.48
101-17	8.92	15.42	0.19	0.09	45.78	0.12	25.79	0.18	96.50
101-19	7.97	15.59	0.18	0.13	46.71	0.17	25.70	0.11	96.55
101-22	8.24	9.40	0.10	0.12	55.21	0.22	25.50	0.24	99.02
101-27	7.95	15.83	0.17	0.15	47.08	0.17	25.67	0.17	97.19

+2.15 to +2.29 m (Rödkarten bed)

Sample weight 11.8 kg.

EC grains >63 μm

Grain	MgO	Al_2O_3	TiO_2	V_2O_3	Cr_2O_3	MnO	FeO	ZnO	Total
102-1	1.97	6.14	2.18	0.59	57.81	0.48	25.46	0.48	95.10
102-2	2.01	5.81	3.11	0.80	60.26	0.55	24.04	1.37	97.95
102-3	2.09	5.35	2.88	0.68	56.49	0.45	30.82	0.22	98.99
102-5	2.54	5.87	3.02	0.71	56.99	0.65	29.48	0.37	99.64
102-6	2.55	5.45	2.99	0.68	57.23	0.43	29.66	0.32	99.31
102-7	2.49	6.19	1.96	0.64	60.62	0.80	22.28	2.94	97.92
102-8	1.90	5.58	3.06	0.82	58.49	0.53	27.54	0.41	98.33
102-9	2.08	6.06	3.11	0.81	58.47	0.42	27.05	0.35	98.35
102-10	2.32	5.91	3.00	0.78	59.61	0.58	23.76	1.31	97.27
102-11	5.38	4.92	3.32	0.82	59.19	0.41	25.48	0.38	99.89
102-12	1.87	5.78	3.00	0.76	58.48	0.34	26.63	0.32	97.17
102-13	2.74	5.46	2.97	0.68	58.21	0.54	28.55	0.37	99.53
102-14	3.20	5.11	2.93	0.64	56.70	0.58	28.39	0.45	97.99
102-16	2.54	5.75	3.19	0.67	57.94	0.51	27.17	0.44	98.20
102-17	4.13	5.52	2.86	0.65	57.80	0.47	27.25	0.28	98.95
102-18	2.17	5.44	2.95	0.60	57.71	0.52	29.69	0.22	99.31
102-19	2.02	5.42	3.10	0.68	57.42	0.43	29.14	0.25	98.45
102-20	2.45	5.31	3.08	0.68	56.70	0.54	30.51	0.24	99.50
102-21	2.68	5.66	3.05	0.76	57.22	0.60	29.80	0.35	100.1
102-22	1.65	5.63	3.06	0.77	58.90	0.42	25.07	0.44	95.93
102-24	2.24	5.74	3.26	0.72	60.10	0.46	25.00	0.26	97.77
102-25	2.11	5.61	3.00	0.78	58.24	0.51	27.47	0.41	98.13
102-26	1.70	5.52	3.13	0.69	59.32	0.48	22.83	0.38	94.06
102-27	2.49	5.53	3.28	0.69	58.45	0.52	29.53	0.27	100.8

OtC grains >63 μm

Grain	MgO	Al_2O_3	TiO_2	V_2O_3	Cr_2O_3	MnO	FeO	ZnO	Total
102-15	10.17	13.74	0.21	0.09	53.45	0.15	20.35	0.16	98.31
102-4	1.38	10.17	1.25	2.94	28.71	1.03	46.64	2.78	94.90

+0.87 to +1.05 m (Botten bed)

Sample weight 24.6 kg. From Schmitz and Häggström (2006).

EC grains >63 μm

Grain	MgO	Al_2O_3	TiO_2	V_2O_3	Cr_2O_3	MnO	FeO	ZnO	Total
1	2.13	5.22	2.90	0.59	55.84	0.60	31.63	0.20	99.11
2	2.43	6.54	3.00	0.77	56.83	0.74	30.97	0.23	101.5
3	2.14	4.85	3.32	0.65	57.24	0.81	29.69	0.19*	98.89
4	3.09	6.50	3.09	0.79	55.22	0.69	29.39	0.27	99.04
5	2.33	6.78	3.26	0.64	57.71	0.69	30.23	0.19*	101.8
6	2.49	7.27	2.83	0.75	54.65	0.84	28.59	0.40	97.82

7	3.39	6.09	3.18	0.70	55.70	0.81	28.53	0.36	98.76
8	2.09	5.86	3.25	0.75	55.51	0.85	29.90	0.33	98.54
9	2.19	5.78	2.94	0.73	56.58	0.90	28.83	0.27	98.22
10	2.42	6.15	3.09	0.60	55.87	0.87	29.86	0.33	99.19
11	2.72	5.87	3.13	0.72	55.89	0.92	30.38	0.40	100.0
12	2.52	4.73	2.78	0.68	57.40	0.93	29.55	0.38	98.97
13	1.88	5.19	3.02	0.75	56.66	0.69	29.80	0.32	98.31
14	2.46	5.47	3.42	0.74	57.44	0.79	30.93	0.39	101.6
15	2.25	5.86	3.16	0.79	56.83	0.66	30.82	0.27	100.6
16	3.23	5.77	3.01	0.69	57.05	0.77	28.36	0.35	99.23
17	2.84	5.80	3.35	0.73	58.05	0.76	30.80	0.43	102.8
18	2.78	6.55	3.21	0.76	57.10	0.77	31.01	0.23	102.4
19	2.62	5.90	3.22	0.80	56.80	0.88	30.14	0.41	100.8
20	2.42	6.21	3.09	0.83	55.93	0.70	31.35	0.22	100.8
21	2.26	5.93	3.23	0.74	54.57	0.86	30.32	0.30	98.21
22	2.93	6.18	3.15	0.77	55.77	0.85	29.66	0.37	99.68
23	2.60	5.84	3.26	0.81	56.96	0.75	30.30	0.31	100.8

*Values lower than the detection limit (<2 sigma).

OtC-V grains >63 μm

Grain	MgO	Al_2O_3	TiO_2	V_2O_3	Cr_2O_3	MnO	FeO	ZnO	Total
1	7.28	6.85	0.02*	0.48	61.85	0.50	22.27	0.23	99.48
2	3.35	17.14	1.42	0.72	48.39	0.79	28.68	0.03*	100.5

*Values lower than the detection limit (<2 sigma).

OtC grains >63 μm

Grain	MgO	Al_2O_3	TiO_2	V_2O_3	Cr_2O_3	MnO	FeO	ZnO	Total
1	9.35	12.75	0.07*	0.40	55.00	0.43	21.25	0.15*	99.40
2	10.37	15.81	0.42	0.22	42.00	0.34	29.47	0.13*	98.76
3	10.51	13.80	0.00*	0.21	50.24	0.53	24.80	0.23	100.3
4	11.10	13.46	0.01*	0.26	57.05	0.37	17.80	0.19	100.2
5	5.22	5.34	0.09*	0.27	56.42	0.66	29.64	0.33	97.97
6	10.74	7.32	0.06*	0.30	60.99	0.44	18.05	0.08*	97.98
7	7.83	13.66	0.05*	0.25	48.27	0.58	27.15	0.19	97.98
8	12.09	21.47	0.32	0.17	41.91	0.36	23.14	0.20	99.66
9	6.00	11.26	1.36	0.26	28.31	0.56	47.84	0.15*	95.74
10	9.00	8.19	0.28	0.08*	50.87	0.56	27.90	0.18*	97.06
11	6.32	6.05	0.07*	0.17	58.07	0.41	28.75	0.27	100.1
12	9.27	10.02	0.00*	0.26	61.07	0.21*	19.36	0.25	100.4
13	8.60	13.21	1.86	0.29	21.62	0.27	52.24	0.26	98.35
14	10.48	19.75	0.25	0.07*	45.54	0.44	24.36	0.17*	101.1
15	10.67	14.56	0.10*	0.23	43.54	0.28	27.90	0.18*	97.46
16	6.25	7.03	0.16	0.18	58.79	0.51	25.18	0.16*	98.26
17	11.55	23.60	0.04*	0.25	43.21	0.25	21.04	0.24	100.2
18	7.94	12.62	0.68	0.17	37.90	0.33	38.65	0.04*	98.33
19	9.97	8.53	0.04*	0.24	61.90	0.45	20.25	0.16*	101.5
20	9.12	12.70	0.00*	0.36	55.27	0.46	21.49	0.16*	99.56
21	10.78	15.69	0.03*	0.20	55.77	0.26	19.09	0.14*	102.0
22	10.63	17.08	0.16	0.26	51.02	0.39	19.50	0.27	99.31
23	9.36	8.49	0.11	0.23	58.57	0.41	21.22	0.13*	98.52
24	10.85	18.06	0.48	0.26	40.74	0.43	27.95	0.20	98.97
25	9.29	9.84	0.01*	0.26	58.04	0.36	22.65	0.16*	100.6
26	12.13	25.54	0.29	0.21	41.84	0.45	20.74	0.31	101.5
27	2.95	3.81	0.94	0.44	39.59	0.80	49.69	0.04*	98.26
28	11.35	17.57	0.63	0.29	36.83	0.37	33.73	0.08*	100.9

29	11.95	16.18	0.19	0.05*	48.41	0.41	21.48	0.12*	98.79
30	11.39	27.14	0.25	0.18	36.61	0.49	23.57	0.20	99.83
31	11.01	13.70	0.10*	0.17	51.51	0.47	22.42	0.18*	99.56
32	9.68	15.38	0.32	0.10*	45.49	0.57	26.83	0.15*	98.52

*Values lower than the detection limit (<2 sigma).

+0.77 to +0.87 m (Golvsten bed)

Sample weight 13.3 kg. From Schmitz and Häggström (2006).

EC grains >63 μm

Grain	MgO	Al_2O_3	TiO_2	V_2O_3	Cr_2O_3	MnO	FeO	ZnO	Total
1	2.57	6.22	3.19	0.68	57.54	0.87	29.39	0.26	100.7
2	2.04	5.79	3.05	0.72	58.35	0.85	28.84	0.41	100.1
3	2.39	5.84	2.98	0.68	54.51	0.92	30.56	0.24	98.12
4	3.62	4.77	3.24	0.79	58.47	0.82	27.26	0.45	99.42
5	3.61	5.88	3.33	0.67	56.55	0.91	27.19	0.36	98.50
6	2.21	6.45	2.43	0.77	58.44	0.79	26.91	0.29	98.29
7	2.93	5.72	3.11	0.63	56.03	0.89	30.04	0.14	99.49
8	2.43	6.79	2.97	0.73	56.61	0.84	29.57	0.42	100.4
9	2.04	5.99	3.09	0.80	56.74	0.66	29.21	0.29	98.82
10	2.01	4.75	2.80	0.73	55.48	0.94	29.51	0.35	96.57
11	3.08	6.52	3.44	0.79	57.06	0.86	29.88	0.30	101.9
12	2.15	5.70	3.17	0.74	55.48	0.88	30.49	0.32	98.93
13	1.65	3.94	2.12	0.87	60.99	0.86	27.05	0.82	98.30
14	2.75	6.37	2.51	0.72	58.67	0.96	27.21	0.42	99.61
15	2.83	5.42	3.50	0.79	58.48	1.00	29.65	0.77	102.4
16	2.38	5.62	3.37	0.80	57.22	0.81	29.03	0.31	99.54
17	3.28	5.34	3.27	0.70	58.50	0.65	26.90	0.43	99.07
18	2.39	5.79	3.17	0.71	58.24	0.89	28.65	0.28	100.1
19	3.59	6.00	2.07	0.75	57.60	0.81	27.10	0.43	98.35
20	2.64	6.17	3.11	0.69	57.22	0.81	28.67	0.25	99.56
21	2.55	6.36	3.13	0.74	56.94	0.74	30.43	0.26	101.2
22	2.18	6.11	3.31	0.72	57.54	0.78	28.83	0.45	99.92
23	3.25	5.31	3.30	0.74	57.71	0.88	28.41	0.29	99.89
24	2.73	5.31	3.42	0.69	57.45	0.89	28.82	0.33	99.64
25	2.81	6.29	3.28	0.79	58.21	0.94	28.09	0.43	100.8
26	2.12	5.46	3.33	0.76	58.79	0.77	29.13	0.43	100.8
27	2.43	6.41	3.37	0.76	59.46	0.88	25.70	0.39	99.40
28	2.86	6.04	2.72	0.80	56.14	0.85	28.56	0.29	98.26

OtC-V grains >63 μm

Grain	MgO	Al_2O_3	TiO_2	V_2O_3	Cr_2O_3	MnO	FeO	ZnO	Total
2	6.41	4.24	0.71	0.46	60.36	0.32	25.29	0.22	98.01

OtC grains >63 μm

Grain	MgO	Al_2O_3	TiO_2	V_2O_3	Cr_2O_3	MnO	FeO	ZnO	Total
1	15.64	28.22	0.35	0.17	36.70	0.31	17.01	0.02*	98.42
2	12.30	22.62	0.96	0.21	42.40	0.43	21.52	0.03*	100.5
1	9.14	19.83	1.09	0.51	23.11	0.33	45.85	0.12*	99.98

*Values lower than the detection limit (<2 sigma).

+0.62 to +0.77 m (Golvsten bed)

Sample weight 17.7 kg. From Schmitz and Häggström (2006).

EC grains >63 µm

Grain	MgO	Al_2O_3	TiO_2	V_2O_3	Cr_2O_3	MnO	FeO	ZnO	Total
1	2.71	5.40	3.45	0.67	56.70	0.81	27.98	0.36	98.08
2	2.14	6.48	3.11	0.76	56.62	0.86	29.15	0.37	99.49
3	2.80	5.48	3.33	0.73	56.61	0.93	29.31	0.35	99.54
4	3.00	5.50	3.25	0.71	57.04	1.11	28.21	0.45	99.27
5	3.15	8.02	1.90	0.83	62.14	1.51	21.40	2.58	101.5
6	3.04	6.77	2.15	0.96	59.17	1.65	25.03	1.17	99.94
7	2.01	5.53	3.06	0.74	59.17	1.16	25.01	0.97	97.65
8	2.41	5.25	2.91	0.83	57.16	0.79	29.99	0.42	99.76
9	2.35	6.82	2.80	0.87	57.26	1.14	28.52	0.47	100.2
10	2.62	5.17	3.32	0.79	58.35	1.12	28.02	0.53	99.92
11	2.26	5.36	2.99	0.64	56.11	0.85	28.39	0.53	97.13
12	1.72	5.93	2.96	0.79	56.14	0.56	29.38	0.14*	97.62
13	3.10	5.76	3.21	0.83	56.84	0.66	28.78	0.21	99.39
14	1.88	6.80	2.71	0.77	59.29	0.87	25.20	1.97	99.49
15	3.04	6.10	3.23	0.74	57.11	0.99	29.30	0.50	101.0
16	2.28	5.01	3.05	0.78	57.16	0.69	29.06	0.26	98.29
17	1.53	5.63	3.41	0.74	60.08	0.46	25.33	0.95	98.13
18	3.41	5.32	3.21	0.71	57.42	0.91	27.78	0.25	99.01
19	3.69	6.27	3.36	0.76	58.55	0.61	27.38	0.84	101.5
20	2.85	5.47	3.33	0.68	56.05	0.79	29.82	0.28	99.27
21	2.54	6.63	2.00	0.89	60.83	0.64	25.26	0.68	99.47
22	2.02	6.66	2.96	0.87	59.57	0.82	22.71	2.22	97.83
23	2.86	5.70	2.57	0.98	57.84	1.33	25.13	1.35	97.76
24	2.30	5.78	3.29	0.74	58.04	0.81	28.35	0.41	99.72
25	2.81	6.05	2.28	0.89	59.17	1.85	22.86	1.41	97.32
26	2.79	5.88	3.28	0.70	57.32	0.90	28.07	0.22	99.16
27	3.01	7.99	2.64	0.74	57.79	0.95	23.79	1.11	98.02
28	3.66	6.77	3.02	0.62	56.52	0.70	28.13	0.32	99.74
29	2.49	5.53	2.96	0.83	57.15	1.53	28.16	0.60	99.25
30	1.78	6.04	3.22	0.85	59.53	0.49	24.94	0.38	97.23

*Values lower than the detection limit (<2 sigma).

OtC grains >63 µm

Grain	MgO	Al_2O_3	TiO_2	V_2O_3	Cr_2O_3	MnO	FeO	ZnO	Total
1	14.90	31.13	0.10*	0.21	37.65	0.31	15.24	0.26	99.80

*Values lower than the detection limit (<2 sigma).

+0.32 to +0.56 m (Arkeologen bed)

Sample weight 24.0 kg. From Schmitz and Häggström (2006).

EC grains >63 µm

Grain	MgO	Al_2O_3	TiO_2	V_2O_3	Cr_2O_3	MnO	FeO	ZnO	Total

1	1.99	6.93	3.40	0.67	58.13	0.42	28.74	0.17*	100.5
2	3.83	6.47	3.21	0.54	57.04	0.60	27.19	0.28	99.16
3	2.34	7.16	3.25	0.83	59.29	0.83	24.84	0.34	98.88
4	3.33	5.91	3.31	0.74	56.81	1.07	28.45	0.46	100.1
5	2.53	6.28	3.30	0.68	56.58	0.55	30.37	0.25	100.5
6	2.62	6.74	3.23	0.76	58.61	0.77	26.99	0.38	100.1
7	2.28	5.54	2.65	0.79	56.22	0.61	28.83	0.35	97.27
8	3.04	6.39	3.20	0.80	55.65	0.76	28.06	0.38	98.28
9	2.30	7.54	3.46	0.78	59.14	0.47	24.82	1.17	99.68
10	2.47	6.17	2.60	0.74	55.20	0.86	28.77	0.40	97.21
11	2.96	6.93	3.35	0.80	58.43	0.72	25.36	0.50	99.05
12	3.60	9.15	2.49	0.80	57.02	0.54	27.44	0.28	101.3
13	4.30	7.40	3.29	0.64	58.99	0.44	23.12	0.42	98.60
14	1.98	6.16	3.20	0.79	57.34	0.48	26.97	0.31	97.23
15	3.30	6.49	3.35	0.80	56.83	0.87	28.75	0.48	100.9
16	2.18	7.26	3.22	0.79	60.73	0.95	20.94	3.18	99.25
17	2.81	6.17	3.30	0.74	58.29	0.65	28.05	0.61	100.6
18	2.33	6.07	2.87	0.79	57.37	0.79	28.67	0.45	99.34
19	1.93	5.28	3.03	0.80	56.21	0.63	29.64	0.19*	97.71
20	3.44	6.03	3.24	0.67	57.09	0.89	28.71	0.59	100.7
21	1.25	4.44	2.49	0.93	58.50	1.03	20.15	2.02	90.81
22	2.44	5.72	2.93	0.69	55.42	0.74	28.70	0.31	96.95
23	2.15	5.91	3.66	0.77	61.83	0.27*	19.53	0.57	94.69
24	2.76	5.45	3.29	0.67	56.29	0.89	28.69	0.44	98.48
25	3.47	5.62	3.28	0.79	57.60	0.74	28.10	0.56	100.2
26	2.82	5.62	3.22	0.83	57.01	1.13	28.38	0.49	99.50
27	2.72	5.92	3.47	0.80	58.84	0.83	22.84	1.09	96.51
28	2.52	6.03	3.10	0.69	58.83	0.72	26.52	0.73	99.14
29	3.42	6.20	3.19	0.75	55.35	0.82	29.66	0.22*	99.61
30	3.20	5.94	3.06	0.73	56.81	1.01	28.36	0.44	99.55
31	4.15	6.91	2.97	0.83	58.08	0.89	26.10	0.47	100.4
32	3.60	6.08	3.24	0.69	56.72	0.96	28.57	0.48	100.3
33	3.04	5.95	3.30	0.68	57.22	0.78	28.62	0.29	99.88
34	2.59	6.86	3.29	0.76	56.59	0.97	29.88	0.72	101.7
35	2.04	5.86	3.12	0.79	56.78	0.74	28.50	0.52	98.35
36	2.94	6.06	2.99	0.81	56.46	1.19	28.00	0.63	99.08
37	2.86	6.48	3.08	0.80	56.87	0.71	27.89	0.34	99.03
38	1.90	5.80	2.89	0.82	61.17	0.63	20.15	0.58	93.94
39	2.17	5.42	2.41	0.77	57.79	0.99	27.70	0.43	97.68
40	3.22	6.55	3.03	0.73	56.12	0.82	29.15	0.33	99.95
41	2.64	5.92	2.89	0.78	55.79	0.76	27.24	0.42	96.44

*Values lower than the detection limit (<2 sigma).

OtC grains >63 µm

Grain	MgO	Al_2O_3	TiO_2	V_2O_3	Cr_2O_3	MnO	FeO	ZnO	Total
1	9.43	8.26	0.14	0.12*	56.51	0.39	24.51	0.11*	99.47
2	9.13	10.82	0.07*	0.07*	53.75	0.25*	24.95	0.11*	99.15
3	13.68	42.84	0.00*	0.38	26.57	0.07*	15.52	0.32*	99.38

*Values lower than the detection limit (<2 sigma).

+0.08 to +0.32 m (Arkeologen bed)

Sample weight 26.0 kg. From Schmitz and Häggström (2006).

EC grains >63 μm

Grain	MgO	Al_2O_3	TiO_2	V_2O_3	Cr_2O_3	MnO	FeO	ZnO	Total
1	4.14	6.03	3.14	0.66	56.26	0.70	26.35	0.21	97.49
2	1.55	5.48	3.62	0.79	58.27	0.51	24.97	0.40	95.59
3	3.49	6.49	2.22	0.74	57.16	0.91	26.47	0.43	97.91
4	5.84	6.57	2.40	0.73	56.75	0.54	23.12	0.74	96.69
5	2.83	6.26	3.26	0.73	56.44	0.71	29.48	0.46	100.2
6	2.89	5.50	3.21	0.74	57.77	0.76	28.19	0.82	99.88
7	2.49	5.53	3.18	0.69	57.18	0.88	28.09	0.16*	98.20
8	3.61	5.89	3.26	0.76	56.86	0.66	27.99	0.27	99.30
9	2.87	5.81	2.95	0.84	56.88	1.02	28.43	0.21*	99.01
10	2.08	6.53	2.51	0.83	60.12	1.13	19.10	4.07	96.37
11	2.34	5.98	3.36	0.75	57.68	0.56	28.35	0.39	99.41
12	2.37	5.28	2.79	0.62	56.71	0.97	28.17	0.55	97.46
13	2.55	6.59	2.87	0.84	59.58	0.78	26.67	0.38	100.3
14	1.97	5.68	3.25	0.79	59.07	0.61	26.24	0.34	97.95
15	3.04	5.98	3.17	0.79	57.97	0.69	28.39	0.44	100.5
16	2.22	4.62	3.07	0.79	57.43	0.92	27.99	0.49	97.53
17	2.67	5.83	3.50	0.67	57.78	0.74	29.82	0.42	101.4
18	2.90	6.27	3.43	0.69	58.25	1.05	27.42	0.40	100.4
19	1.84	5.30	3.08	0.86	58.85	0.92	29.06	0.31	100.2
20	2.82	5.44	3.22	0.68	58.05	0.59	27.49	0.48	98.77
21	3.01	4.94	2.88	0.93	57.96	0.99	26.63	0.68	98.02
22	3.19	5.55	3.56	0.71	57.12	0.77	28.40	0.46	99.76
23	1.66	6.66	3.06	0.74	59.75	0.59	22.90	0.34	95.70
24	1.94	6.72	3.42	0.72	58.79	0.72	25.31	0.29	97.91
25	2.28	7.44	2.14	0.85	58.19	1.13	22.89	4.06	98.98
26	3.98	6.17	3.56	0.71	57.32	0.75	28.40	0.46	101.4
27	2.39	6.78	3.14	0.83	59.35	0.83	25.40	0.47	99.19
28	2.58	5.50	3.04	0.64	56.82	0.62	29.38	0.30	98.88
29	2.05	5.78	3.13	0.78	56.81	0.86	28.80	0.34	98.55
30	2.43	5.90	3.22	0.64	58.31	0.68	28.64	0.30	100.1
31	2.07	5.54	3.52	0.69	59.08	0.76	24.99	0.53	97.18
32	3.68	5.99	3.32	0.81	58.72	0.85	26.33	0.40	100.1
33	2.65	6.18	3.00	0.71	56.97	0.61	28.58	0.58	99.28
34	4.18	6.59	3.26	0.71	57.43	0.85	27.05	0.59	100.7
35	2.53	6.02	3.24	0.75	56.73	0.70	27.73	0.29	97.99
36	3.61	5.72	3.21	0.77	56.61	0.84	27.25	0.34	98.35
37	2.81	5.59	3.47	0.65	56.86	0.81	29.08	0.30	99.57
38	2.74	4.97	3.75	0.79	58.14	1.02	28.34	1.17	100.9
39	2.48	6.48	3.14	0.79	57.76	0.75	28.13	0.52	100.1
40	3.55	6.57	3.07	0.66	60.01	0.60	23.45	0.67	98.58
41	3.09	6.85	3.08	0.64	57.63	1.08	23.71	2.77	98.85
42	2.65	5.26	3.38	0.72	58.42	0.65	28.45	0.52	100.1
43	1.28	6.76	2.45	0.63	53.17	0.47	34.17	0.54	99.47
44	2.12	6.45	3.20	0.71	59.60	0.54	25.07	0.40	98.09
45	3.10	6.04	3.27	0.70	56.56	0.98	28.63	0.53	99.81
46	2.59	5.48	3.36	0.69	57.36	0.67	28.53	0.33	99.01
47	3.36	6.40	2.94	0.64	56.60	0.87	28.81	0.47	100.1
48	2.11	7.35	3.26	0.79	60.74	0.67	21.95	0.92	97.79
49	3.24	5.66	3.31	0.72	56.61	1.11	27.82	0.51	98.98
50	2.17	6.26	3.32	0.80	58.15	0.62	27.07	0.46	98.85
51	2.27	6.29	3.24	0.78	58.68	0.80	27.35	0.37	99.78
52	3.27	5.18	3.41	0.69	57.44	0.97	27.23	0.95	99.14
53	2.32	5.27	3.17	0.81	58.18	0.93	29.16	0.38	100.2
54	3.11	6.07	3.32	0.81	57.73	0.79	29.29	0.41	101.5

55	2.17	5.28	3.45	0.75	57.85	0.79	27.53	0.65	98.47
56	2.08	5.77	3.20	0.81	58.88	0.84	27.84	0.60	100.0
57	2.89	6.36	3.18	0.69	56.80	0.66	29.09	0.72	100.4
58	2.05	6.48	2.99	0.72	57.41	0.67	28.31	0.32	98.95
59	2.62	6.53	2.57	0.92	57.41	1.13	28.40	0.61	100.2
60	1.52	7.20	3.15	0.77	57.27	0.51	25.87	0.89	97.18
61	2.63	6.11	3.27	0.72	57.60	0.79	28.49	0.49	100.1
62	1.78	6.83	3.04	0.96	60.07	0.77	25.63	0.35	99.43
63	2.81	6.91	3.13	0.79	60.51	0.68	23.87	1.31	100.0
64	2.86	6.26	3.24	0.72	57.96	0.80	28.09	1.11	101.0
65	1.40	6.49	3.75	0.74	59.69	0.44	25.19	0.42	98.12
66	2.68	5.73	3.38	0.74	57.79	0.87	29.11	0.68	101.0
67	2.49	7.49	3.22	0.70	58.28	0.61	26.56	0.56	99.91
68	3.18	5.99	2.96	0.74	57.58	0.84	28.24	0.46	99.99
69	2.10	6.00	3.27	0.81	57.04	0.93	30.56	0.36	101.1
70	4.31	6.00	3.05	0.71	58.29	0.65	27.23	0.56	100.8
71	2.68	6.26	3.05	0.77	57.11	0.80	28.38	1.10	100.2
72	2.17	5.85	3.86	0.86	60.27	0.77	26.42	0.21	100.4
73	2.84	5.76	3.26	0.74	57.59	0.77	28.44	0.71	100.1
74	2.80	5.93	3.18	0.82	57.35	0.90	28.30	0.51	99.79
75	1.79	6.36	3.66	0.81	60.15	0.63	24.70	0.50	98.60
76	2.64	6.14	3.20	0.73	58.78	0.67	27.35	0.26	99.77
77	2.81	6.93	3.25	0.83	59.98	0.92	21.82	1.21	97.75
78	2.54	6.28	3.17	0.72	58.46	0.59	25.25	0.56	97.57
79	3.16	7.01	3.50	0.74	60.32	0.57	24.31	0.29	99.90

*Values lower than the detection limit (<2 sigma).

OtC-V grains >63 µm

Grain	MgO	Al_2O_3	TiO_2	V_2O_3	Cr_2O_3	MnO	FeO	ZnO	Total
1	5.92	16.69	1.00	0.56	42.49	0.44	29.22	0.16*	96.48

*Values lower than the detection limit (<2 sigma).

OtC grains >63 µm

Grain	MgO	Al_2O_3	TiO_2	V_2O_3	Cr_2O_3	MnO	FeO	ZnO	Total
1	9.27	17.76	0.18	0.12	44.97	0.39	23.35	0.20	96.24
2	13.10	32.28	0.22*	0.09*	31.54	0.15*	20.33	0.31*	98.02
3	9.95	16.44	0.36	0.22	46.11	0.35	23.96	0.25	97.64
4	10.37	24.53	0.41	0.23	35.72	0.45	25.18	0.14*	97.03
5	12.96	20.70	0.30	0.20	48.13	0.23*	16.90	0.19*	99.61
6	8.68	16.47	0.90	0.13	43.69	0.23	28.14	0.14*	98.38
7	8.09	14.98	0.33	0.24	47.51	0.45	25.83	0.21	97.64
8	9.81	24.84	0.48	0.16	35.17	0.41	27.20	0.20	98.27
9	9.92	22.29	0.46	0.34	36.97	0.23	27.57	0.19*	97.97
10	12.13	22.86	1.10	0.30	34.37	0.33	29.41	0.14*	100.6
11	9.70	23.08	0.41	0.17	41.63	0.24	22.02	0.18*	97.43
12	14.70	35.32	0.23	0.21	24.49	0.18*	24.85	0.14*	100.1
13	11.56	15.55	1.40	0.28	48.19	0.32	21.42	0.16*	98.88
14	9.46	20.08	0.56	0.21	41.51	0.25	25.07	0.04*	97.18
15	11.85	16.31	0.09	0.09*	50.27	0.30*	22.72	0.33	102.0
16	9.38	14.73	0.40	0.21	45.15	0.26	27.08	0.22	97.43
17	10.90	17.66	0.38	0.16	45.23	0.41	25.08	0.18*	100.0
18	9.76	26.04	0.69	0.40	19.79	0.39	39.99	0.00*	97.06
19	9.38	21.39	0.30	0.14	38.08	0.31	23.82	0.15*	93.57
20	15.10	35.50	0.27	0.23	29.79	0.39	24.34	0.11*	105.7

21	15.21	20.89	0.44	0.14	47.13	0.15*	17.93	0.11*	102.0
22	9.02	19.03	0.53	0.20	43.53	0.42	27.77	0.23	100.7
23	11.07	21.51	0.48	0.13	37.30	0.32	28.45	0.26	99.52
24	10.39	20.79	1.61	0.21	38.96	0.23*	26.13	0.23	98.55
25	10.74	23.93	0.32	0.20	40.22	0.32	26.50	0.16*	102.4
26	12.13	24.24	1.01	0.31	38.36	0.32	23.49	0.31	100.2
27	11.06	21.57	0.36	0.31	36.62	0.33	29.35	0.25	99.85
28	11.43	24.24	0.61	0.19	47.68	0.25	21.45	0.21	106.1
29	6.98	16.18	0.28	0.25	32.04	0.57	43.57	0.23	100.1
30	9.79	20.27	0.62	0.26	39.65	0.41	27.60	0.10*	98.70
31	12.11	28.79	0.49	0.21	38.00	0.33	22.24	0.11*	102.3
32	11.36	16.66	0.40	0.17	48.24	0.44	23.11	0.15*	100.5
33	11.68	23.55	0.13	0.12	41.91	0.33	21.62	0.27	99.61
34	8.84	18.63	0.60	0.23	41.23	0.31	26.65	0.29	96.78
35	9.78	15.95	0.21	0.21	51.60	0.44	22.16	0.30	100.7
36	10.44	13.28	0.39	0.17	48.24	0.36	25.94	0.16*	98.98
37	7.52	15.47	0.47	0.11	43.50	0.52	28.76	0.25	96.60
38	9.27	17.11	0.55	0.18	50.09	0.23	19.90	0.03*	97.36
39	10.42	11.92	0.35	0.07*	50.65	0.37	23.39	0.16*	97.33
40	9.40	18.08	0.35	0.20	42.59	0.39	27.90	0.10*	99.01
41	14.83	38.38	0.29	0.21	21.23	0.27	25.88	0.26	101.4
42	6.99	14.13	0.43	0.18	43.84	0.31	31.11	0.23	97.22
43	10.32	14.82	0.46	0.12	49.64	0.39	23.10	0.05*	98.90
44	9.96	15.66	0.48	0.17	48.24	0.52	25.95	0.12*	101.1
45	10.49	18.61	0.63	0.24	43.78	0.26	24.22	0.17*	98.40
46	10.24	22.08	0.86	0.40	29.31	0.40	37.99	0.15*	101.4
47	11.56	16.66	0.20	0.10*	47.86	0.24	25.68	0.34	102.6
48	10.84	14.00	0.29	0.08*	47.94	0.24	27.03	0.08*	100.5
2	9.01	17.40	2.07	0.54	32.50	0.48	35.79	0.17*	97.96
3	8.12	17.83	2.17	0.67	22.66	0.37	49.20	0.18*	101.2

*Values lower than the detection limit (<2 sigma).

-0.54 to -0.82 m

Sample weight 4.3 kg.

EC grains >63 μm

Grain	MgO	Al_2O_3	TiO_2	V_2O_3	Cr_2O_3	MnO	FeO	ZnO	Total
1	2.84	5.90	3.20	0.73	58.80	0.74	27.81	0.45	100.5
2	2.31	5.69	3.31	0.66	59.13	0.81	28.08	0.49	100.5
3	2.58	6.12	1.92	0.78	59.96	0.89	26.46	1.50	100.2

OtC grains >63 μm

Grain	MgO	Al_2O_3	TiO_2	V_2O_3	Cr_2O_3	MnO	FeO	ZnO	Total
1	14.68	30.55	0.52	n.d.	32.66	n.d.	21.96	n.d.	100.4
2	7.36	10.44	n.d.	n.d.	53.44	0.60	28.80	n.d.	100.6

EC grains 32-63 μm

Grain	MgO	Al_2O_3	TiO_2	V_2O_3	Cr_2O_3	MnO	FeO	ZnO	Total
1	2.62	6.27	3.06	0.73	58.87	0.79	27.44	0.64	100.4

2	2.42	6.37	2.93	0.80	57.84	2.51	26.65	0.94	100.5
3	2.78	6.11	3.09	0.65	58.52	1.01	27.79	0.74	100.7
4	2.49	6.30	3.21	0.69	57.73	0.95	27.84	0.37	99.58
5	3.03	6.14	3.18	0.70	58.49	1.05	25.88	2.15	100.6
6	2.51	6.71	1.76	0.76	60.41	1.14	25.84	0.80	99.92
7	2.36	6.02	3.20	0.79	58.57	0.88	27.76	0.74	100.3
8	2.43	5.89	3.07	0.72	58.34	0.88	28.46	0.56	100.4
9	2.57	6.13	2.96	0.71	58.22	0.77	28.68	0.53	100.6
10	3.24	5.85	3.30	0.68	58.39	0.81	26.84	0.76	99.89
11	2.82	7.05	2.57	0.81	57.25	1.12	26.63	0.60	98.85
12	2.53	6.40	3.04	0.74	58.88	0.93	27.09	0.52	100.1
13	2.58	6.04	3.03	0.82	59.94	1.00	27.50	0.37	101.3
14	2.63	5.97	3.20	0.71	59.70	0.88	27.52	0.70	101.3
15	3.00	6.96	2.50	0.83	59.32	1.30	25.95	1.60	101.5
16	2.48	6.01	3.08	0.73	58.97	0.74	27.21	0.31	99.53
17	2.91	6.08	2.89	0.83	59.18	0.94	27.64	0.70	101.2
18	2.67	5.95	2.90	0.64	59.26	1.00	27.37	0.48	100.3
19	2.94	5.89	3.06	0.59	59.14	0.48	27.42	0.37	99.89
20	2.79	6.16	2.80	0.73	57.25	0.77	26.98	0.98	98.46
21	2.30	6.05	1.90	0.79	61.81	1.15	23.46	2.03	99.48
22	2.44	5.76	2.23	0.73	61.66	0.88	18.35	6.96	99.02
23	2.48	6.83	3.14	0.82	61.81	0.84	22.63	1.13	99.69
24	1.81	6.08	3.26	0.76	60.42	0.91	26.04	0.49	99.78
25	2.23	6.41	2.80	0.78	58.12	0.88	29.24	0.32	100.8
26	2.77	6.48	3.15	0.62	58.43	0.87	28.46	0.45	101.2
27	2.59	5.98	3.05	0.73	58.59	0.73	27.73	0.99	100.4
28	2.49	5.92	3.34	0.68	58.44	0.72	28.56	0.84	101.0
29	2.72	6.06	3.11	0.69	58.41	0.86	27.69	0.96	100.5
30	2.21	5.79	2.84	0.80	59.11	1.01	28.18	0.67	100.6
31	2.36	6.43	2.69	0.77	57.06	1.04	26.39	2.35	99.09
32	2.56	6.12	2.94	0.68	59.10	0.80	28.33	0.94	101.5
33	2.38	6.71	2.45	0.81	58.97	0.93	28.15	0.84	101.3
34	2.84	6.26	3.20	0.72	59.43	0.87	27.31	0.69	101.3
35	2.93	6.22	3.06	0.75	58.93	0.94	27.58	0.79	101.2
36	2.65	6.30	2.41	0.79	59.59	1.34	26.89	1.22	101.2
37	2.50	6.00	3.25	0.78	57.87	1.28	27.87	1.30	100.9
38	2.32	6.65	2.54	0.84	58.38	0.93	28.25	0.82	100.7
39	2.68	5.69	2.95	0.76	58.35	1.04	27.12	1.06	99.66
40	3.61	6.05	3.22	0.78	58.99	0.81	27.26	0.39	101.1

OtC-V grains 32-63 μm

Grain	MgO	Al_2O_3	TiO_2	V_2O_3	Cr_2O_3	MnO	FeO	ZnO	Total
1	7.37	15.34	0.56	1.02	49.51	0.56	25.71	n.d.	100.1
2	11.74	21.80	1.42	0.64	43.64	n.d.	21.28	n.d.	100.5
3	12.78	22.40	1.29	0.56	42.86	0.44	20.60	n.d.	100.9
4	10.07	16.29	0.80	1.05	50.71	n.d.	21.14	n.d.	100.1
5	6.14	15.10	0.84	0.96	48.97	0.60	27.44	n.d.	100.1
6	7.74	19.57	1.41	0.65	44.42	n.d.	26.86	n.d.	100.7
7	9.33	15.86	1.38	0.53	50.25	n.d.	21.67	n.d.	99.02

OtC grains 32-63 μm

Grain	MgO	Al_2O_3	TiO_2	V_2O_3	Cr_2O_3	MnO	FeO	ZnO	Total
1	22.47	11.58	n.d.	n.d.	46.13	0.99	17.84	n.d.	99.00
2	10.56	20.81	1.02	n.d.	44.56	n.d.	22.32	n.d.	99.28

3	11.68	30.02	0.66	n.d.	30.88	n.d.	26.46	n.d.	99.68
4	10.84	24.02	0.40	n.d.	40.14	n.d.	23.37	n.d.	98.77
5	4.09	9.37	1.85	n.d.	16.11	n.d.	63.36	n.d.	94.78
6	9.24	10.41	n.d.	n.d.	56.28	n.d.	23.11	n.d.	99.03
7	11.95	21.73	0.60	n.d.	41.71	0.40	23.83	n.d.	100.2
8	7.34	17.13	3.11	0.33	39.21	n.d.	31.28	n.d.	98.40
9	12.48	29.75	0.67	n.d.	32.61	0.36	24.07	n.d.	99.94
10	15.21	29.61	0.53	n.d.	32.94	n.d.	21.95	n.d.	100.2
11	15.01	30.35	0.57	n.d.	30.93	n.d.	22.97	n.d.	99.83
12	14.72	28.71	0.64	n.d.	32.26	n.d.	23.86	n.d.	100.2
13	13.73	28.79	0.53	n.d.	35.29	n.d.	21.82	n.d.	100.2
14	6.92	20.52	0.47	n.d.	39.85	0.43	30.15	n.d.	98.34
15	12.21	23.78	0.87	0.27	34.18	n.d.	29.39	n.d.	100.7
16	11.06	28.32	0.71	n.d.	31.56	n.d.	28.42	n.d.	100.1
17	14.50	29.13	0.65	n.d.	32.38	n.d.	22.90	n.d.	99.56
18	4.53	8.15	1.33	n.d.	23.25	n.d.	59.20	n.d.	96.46
19	14.63	26.68	0.51	n.d.	36.44	n.d.	22.03	n.d.	100.3
20	4.70	11.88	0.69	n.d.	49.25	n.d.	30.93	n.d.	97.46
21	10.43	22.76	2.34	0.44	39.42	n.d.	25.03	n.d.	100.4
22	6.60	10.39	1.46	0.33	50.49	0.47	29.52	0.30	99.56
23	15.70	31.99	0.48	n.d.	31.95	n.d.	20.38	n.d.	100.5
24	12.22	29.05	0.60	0.26	31.69	n.d.	26.22	n.d.	100.1
25	14.22	28.63	0.66	n.d.	33.25	n.d.	23.21	n.d.	99.97
26	14.85	29.77	0.62	n.d.	32.40	n.d.	22.37	n.d.	100.0
27	4.66	13.89	0.75	n.d.	47.68	n.d.	32.98	0.56	100.5
28	14.39	27.48	0.57	0.26	33.41	n.d.	23.89	n.d.	100.0
29	6.62	12.09	0.54	0.43	46.14	0.91	34.57	0.49	101.8
30	10.75	22.02	0.96	0.41	46.26	n.d.	19.42	n.d.	99.81
31	7.03	17.73	1.57	0.35	27.27	0.26	44.79	n.d.	98.99
32	14.10	29.17	0.60	0.22	32.32	n.d.	23.53	n.d.	99.96
33	15.85	33.65	0.44	n.d.	29.15	n.d.	21.02	n.d.	100.1
34	10.95	23.15	0.72	0.40	39.40	n.d.	25.20	n.d.	99.83
35	12.88	26.22	0.83	0.31	32.05	n.d.	26.98	n.d.	99.28
36	3.84	5.77	n.d.	n.d.	36.56	n.d.	52.80	n.d.	98.98
37	13.81	27.23	0.65	n.d.	32.80	n.d.	24.13	n.d.	98.63
38	14.28	29.66	0.63	n.d.	33.18	n.d.	22.66	n.d.	100.4
39	12.08	27.40	0.69	n.d.	32.64	n.d.	27.23	n.d.	100.0
40	7.87	21.70	1.99	0.77	23.37	n.d.	43.65	n.d.	99.36
41	9.37	16.20	3.07	0.66	26.86	0.36	42.33	n.d.	98.85
42	7.19	12.73	4.90	0.64	27.38	0.32	45.83	n.d.	98.98

-0.77 to -0.82 m

Weight 22.8 kg. From Häggström and Schmitz (2007). Only searched for grains in >63 µm fraction.

EC grains >63 µm

Grain	MgO	Al_2O_3	TiO_2	V_2O_3	Cr_2O_3	MnO	FeO	ZnO	Total
1	1.77	6.60	2.40	0.82	59.71	1.35	20.57	5.50	98.72
2	2.00	7.08	3.28	0.85	65.39	n.d.	20.88	n.d.	99.49
3	2.46	6.21	3.24	0.78	57.34	0.92	27.00	0.80	98.75
4	2.22	6.45	3.15	0.73	59.54	n.d.	27.53	n.d.	99.62
5	2.03	6.47	2.91	0.72	59.85	n.d.	27.00	n.d.	98.98
6	2.24	6.12	2.20	0.78	55.98	0.96	24.99	1.03	94.30

OtC grain >63 μm

Grain	MgO	Al_2O_3	TiO_2	V_2O_3	Cr_2O_3	MnO	FeO	ZnO	Total
1	9.53	15.41	1.16	n.d.	36.10	n.d.	38.10	n.d.	100.3

-0.82 to -0.97 m

Weight 25.4 kg. From Schmitz and Häggström (2006). Only searched for grains in >63 μm fraction.

OtC grains >63 μm

Grain	MgO	Al_2O_3	TiO_2	V_2O_3	Cr_2O_3	MnO	FeO	ZnO	Total
1	10.46	11.98	0.18	n.d.	56.38	0.54	20.91	n.d.	100.6

-0.82 to -1.05 m

Sample weight 62 kg.

It was very difficult to recover all chrome-spinel grains from the residue in the 32-63 μm fraction of this sample, hence only a minimum estimate of the number of grains could be acquired. We estimate that >80% of the chrome-spinel grains in the residue have been recovered.

EC grains >63 μm

Grain	MgO	Al_2O_3	TiO_2	V_2O_3	Cr_2O_3	MnO	FeO	ZnO	Total
1	2.91	5.67	3.28	0.80	60.30	0.60	26.81	n.d.	100.4
2	2.32	5.62	3.47	0.70	58.28	1.07	27.51	0.69	99.67
3	3.03	5.98	3.34	0.83	60.52	0.79	25.50	0.48	100.5
4	3.02	5.50	3.14	0.68	57.91	1.08	27.65	0.58	99.56
5	2.42	5.88	2.35	0.69	59.78	0.71	27.51	0.57	99.92
7	2.54	5.59	3.11	0.70	58.62	0.94	27.77	n.d.	99.27
9	4.13	6.23	1.36	0.70	61.35	1.30	23.18	1.44	99.68
10	2.88	5.50	3.25	0.67	59.22	0.81	27.73	0.42	100.5
12	2.65	6.12	3.33	0.80	61.31	0.70	24.50	1.36	100.8
14	2.84	6.13	1.97	0.72	61.72	1.00	24.65	1.20	100.2
16	3.01	5.43	3.37	0.68	58.47	0.86	27.86	0.35	100.0
18	2.70	5.51	3.14	0.74	58.90	0.99	27.56	0.67	100.2
19	2.55	5.96	2.40	0.80	61.46	0.98	24.71	0.93	99.79
22	2.01	6.06	2.48	0.83	63.09	0.89	24.12	1.58	101.1
23	2.23	6.05	2.56	0.78	62.79	0.87	24.41	1.06	100.8
24	2.17	5.92	2.20	0.88	61.56	0.75	25.42	0.66	99.46

OtC-V grains >63 μm

Grain	MgO	Al_2O_3	TiO_2	V_2O_3	Cr_2O_3	MnO	FeO	ZnO	Total
17	3.68	5.80	0.86	0.51	63.35	0.99	24.80	0.54	100.5

OtC grains >63 μm

Grain	MgO	Al_2O_3	TiO_2	V_2O_3	Cr_2O_3	MnO	FeO	ZnO	Total
6	7.93	16.58	0.68	0.21	41.28	0.41	33.19	n.d.	100.3
8	3.03	11.60	0.94	0.27	50.93	1.26	30.91	0.60	99.55
11	13.60	27.54	0.32	n.d.	46.67	n.d.	11.98	n.d.	100.1
13	10.71	12.68	n.d.	n.d.	54.27	n.d.	21.81	n.d.	99.46
15	10.96	12.45	0.20	n.d.	54.60	n.d.	21.62	n.d.	99.83
20	3.34	6.42	0.21	n.d.	61.63	0.62	28.86	n.d.	101.1
21	11.05	14.67	0.37	n.d.	54.34	n.d.	19.99	n.d.	100.4

EC grains 32-63 μm (plus 49 EC grains only semi-quantitatively analysed in unpolished state, results not included here)

Grain	MgO	Al_2O_3	TiO_2	V_2O_3	Cr_2O_3	MnO	FeO	ZnO	Total
7	1.66	6.03	1.60	0.91	67.97	0.92	20.45	1.10	100.6
8	2.75	5.77	3.22	0.66	60.07	0.86	27.06	0.46	100.9
10	1.82	5.47	3.43	0.77	59.10	0.75	27.56	1.40	100.3
11	3.48	5.58	3.19	0.75	58.68	0.71	27.43	0.60	100.4
15	1.96	6.24	3.59	0.75	64.59	0.82	21.96	0.78	100.7
19	2.17	6.33	3.18	0.81	60.12	1.05	25.96	0.79	100.4
21	2.50	5.67	3.08	0.68	58.78	0.96	27.80	0.93	100.4
22	2.07	6.23	2.97	0.82	62.24	0.66	24.26	0.91	100.2
23	2.52	5.98	3.34	0.75	61.10	0.84	25.05	1.13	100.7
25	2.86	5.05	3.32	0.72	58.75	1.11	25.86	2.56	100.2
26	2.25	5.15	3.15	0.76	59.92	0.85	27.57	0.54	100.2
27	2.75	7.55	1.90	0.82	62.51	1.05	22.72	0.98	100.3
29	2.17	6.12	2.45	0.79	62.91	0.79	24.75	0.92	100.9
30	2.87	5.61	3.18	0.67	59.04	1.05	27.52	0.95	100.9
31	2.32	5.99	2.84	0.70	58.52	1.09	27.99	1.16	100.6
34	2.59	5.75	3.05	0.69	58.93	0.82	28.40	0.89	101.1
36	2.47	5.68	3.20	0.70	58.95	0.96	27.68	1.35	101.0
37	2.61	5.66	2.96	0.68	58.79	0.97	27.59	1.18	100.4
38	2.55	6.32	1.61	0.79	63.64	1.07	19.46	4.80	100.3
39	8.78	4.83	1.35	0.66	64.00	0.68	20.33	0.54	101.2
41	2.02	6.29	3.04	0.85	62.89	0.73	24.11	0.97	100.9
44	1.94	6.63	3.81	0.76	65.98	0.86	20.32	0.63	100.9
47	2.85	6.41	2.85	0.85	63.04	1.78	21.23	1.99	101.0
48	2.16	6.85	2.70	0.86	64.13	1.23	21.31	1.12	100.4
50	2.52	6.46	2.30	0.88	67.58	0.78	19.40	0.76	100.7
51	2.37	5.82	1.94	0.80	61.13	1.13	25.63	1.59	100.4
52	2.51	5.85	3.23	0.74	60.29	0.82	26.76	0.61	100.8
5	2.70	6.16	2.01	0.86	63.18	1.03	22.76	1.64	100.3
6	2.83	5.73	3.16	0.69	58.17	1.24	27.01	1.16	99.98
8	2.24	5.74	2.14	0.84	58.94	1.13	26.52	1.65	99.21
9	1.75	5.99	3.32	0.80	61.48	0.76	25.09	0.46	99.65
15	2.28	5.84	3.34	0.68	60.43	0.94	25.73	0.53	99.77
19	1.86	6.04	2.33	0.77	62.30	1.08	25.04	0.82	100.2
24a	2.46	5.51	3.14	0.70	58.85	0.93	28.40	0.53	100.5
25	2.42	5.77	3.17	0.71	59.93	0.76	27.55	0.57	100.9
28	2.21	6.18	3.54	0.70	64.72	0.88	20.86	1.52	100.6
30	2.81	6.06	3.36	0.81	60.63	0.9	25.21	0.89	100.7
32	3.54	5.72	3.37	0.72	58.97	0.71	27.82	0.41	101.3
34	2.51	6.00	3.01	0.70	59.45	0.93	27.56	0.60	100.8
37	2.05	5.80	3.12	0.74	59.23	0.84	28.62	0.44	100.9
38	4.12	4.61	2.74	0.72	61.64	1.71	22.56	2.85	101.0
41	2.16	5.32	2.57	0.80	60.82	2.52	22.60	2.93	99.73

42	2.41	5.92	3.28	0.73	61.38	0.89	24.32	1.14	100.1
43	1.98	6.41	2.56	0.86	60.86	0.95	24.32	1.90	99.85
45	2.37	5.64	3.21	0.74	58.92	1.23	24.87	3.99	101.0
48	1.92	6.13	3.01	0.77	61.85	0.77	25.07	0.93	100.4
49a	2.45	5.37	3.32	0.66	59.22	0.83	28.42	0.49	100.8
50	2.58	5.77	3.67	0.72	62.52	0.92	22.93	1.47	100.6
51	2.57	5.56	2.99	0.73	59.16	0.95	27.92	0.59	100.5
52	3.15	6.10	3.57	0.71	60.88	0.66	26.07	0.52	101.7
54	2.37	6.60	3.89	0.72	65.81	0.96	20.14	0.76	101.3
55	1.78	6.82	3.44	0.82	66.73	0.85	19.98	0.55	101.0
56	2.79	6.40	4.34	0.72	60.77	0.63	24.48	n.d.	100.1
1a	2.42	5.82	3.28	0.64	58.25	1.08	28.28	0.69	100.5
3	2.12	6.16	2.97	0.86	59.69	1.00	27.37	0.83	101.0
7	1.70	6.59	3.49	0.84	66.09	1.03	20.53	0.69	101.0
11	2.71	7.13	2.55	0.79	65.91	1.23	19.29	0.89	100.5
12	1.99	6.23	3.44	0.78	65.40	1.02	20.72	1.04	100.6
15a	2.41	5.62	2.79	0.64	58.70	0.95	27.18	1.45	99.73
16	2.22	6.16	2.50	0.81	61.28	1.01	26.55	0.68	101.2
21	1.99	5.75	3.07	0.77	58.54	0.95	29.59	0.47	101.1
23a	2.30	6.01	2.86	0.75	58.91	1.07	27.87	1.15	100.9
24	2.10	5.90	3.04	0.72	58.93	0.85	27.96	n.d.	99.49
30	3.21	5.27	3.21	0.83	58.69	1.91	25.26	1.67	100.0
32	2.35	5.95	3.35	0.69	63.24	0.85	24.50	n.d.	100.9
35	2.43	5.83	3.20	0.78	58.32	1.02	28.54	0.76	100.9
38	2.33	6.06	3.09	0.72	59.14	1.64	25.84	1.92	100.8
39	2.45	5.93	3.15	0.79	60.52	0.97	26.57	0.61	101.0
40	5.44	6.95	1.18	0.71	62.13	3.14	17.16	3.91	100.6
44	2.68	5.79	3.44	0.65	58.33	0.96	28.22	n.d.	100.1

OtC-V grains 32-63 μm

Grain	MgO	Al_2O_3	TiO_2	V_2O_3	Cr_2O_3	MnO	FeO	ZnO	Total
3	11.65	17.57	0.87	0.54	43.84	n.d.	26.41	n.d.	100.9
45	4.51	14.53	1.06	0.45	60.59	0.79	17.11	1.54	100.6
1	7.28	15.34	0.71	0.81	52.05	0.63	23.41	n.d.	100.2
20	8.54	15.19	0.57	0.82	51.93	0.62	23.16	n.d.	100.8
8a	4.48	18.35	1.38	0.69	46.06	n.d.	29.39	n.d.	100.4
19	7.22	15.81	0.71	0.73	51.95	0.57	23.62	n.d.	100.6
31	7.43	14.67	0.57	0.87	52.81	0.53	23.96	0.35	101.2
37b	4.11	17.41	0.72	1.22	53.60	n.d.	23.89	n.d.	101.0
47	10.99	23.71	1.87	0.79	40.99	n.d.	22.17	n.d.	100.5
49	8.22	17.31	0.63	0.77	52.22	0.60	21.01	n.d.	100.8

OtC grains 32-63 μm (plus 54 OtC grains only semi-quantitatively analysed in unpolished state, results not included here)

Grain	MgO	Al_2O_3	TiO_2	V_2O_3	Cr_2O_3	MnO	FeO	ZnO	Total
1	10.65	11.64	0.31	n.d.	56.48	n.d.	21.07	n.d.	100.2
2	5.93	11.79	1.33	0.37	37.22	n.d.	43.72	0.36	100.7
4	11.78	22.82	0.36	0.26	41.72	n.d.	23.58	n.d.	100.5
5	4.33	10.26	0.25	n.d.	52.92	0.55	31.42	0.39	100.1
6	5.86	8.35	0.28	n.d.	62.99	0.44	22.85	n.d.	100.8
9	8.82	20.31	0.43	n.d.	43.18	n.d.	26.44	n.d.	99.18
12	13.37	20.13	0.92	n.d.	48.72	0.37	17.23	n.d.	100.7
13	20.73	6.33	0.18	n.d.	62.26	0.75	10.23	n.d.	100.5

16	5.65	12.82	0.53	n.d.	55.89	n.d.	24.65	0.57	100.1
17	11.53	25.02	0.45	n.d.	37.22	0.40	26.79	n.d.	101.4
18	10.94	26.34	0.53	0.21	33.06	0.45	28.69	n.d.	100.2
20	6.87	5.81	n.d.	n.d.	66.34	n.d.	20.87	0.44	100.3
24	8.41	5.83	n.d.	n.d.	64.87	2.20	14.71	4.92	100.9
28	5.49	14.85	1.71	0.34	39.62	n.d.	37.61	0.30	99.91
32	5.68	8.95	0.55	0.23	45.45	0.67	39.41	n.d.	100.9
33	3.84	9.49	1.15	0.38	41.37	0.53	43.88	n.d.	100.6
35	5.09	7.07	58.97	n.d.	n.d.	0.49	28.25	0.40	100.3
40	6.97	9.01	0.43	n.d.	56.18	0.64	27.3	n.d.	100.5
42	4.83	9.97	0.46	n.d.	50.53	0.54	34.4	0.35	101.1
43	7.95	14.18	1.29	0.42	44.32	0.41	31.91	n.d.	100.5
46	8.59	16.68	0.35	n.d.	51.18	n.d.	22.84	n.d.	99.64
49	20.33	9.32	n.d.	n.d.	55.54	1.11	13.40	n.d.	99.70
53	4.68	4.58	n.d.	n.d.	61.54	0.65	27.39	2.23	101.1
54	9.54	37.12	n.d.	n.d.	22.41	0.29	30.51	0.42	100.3
55	2.88	9.06	0.45	n.d.	54.41	0.74	32.79	n.d.	100.3
56	6.51	16.58	0.38	n.d.	56.69	3.48	14.21	2.51	100.4
2	4.98	12.07	0.86	n.d.	41.60	0.51	39.77	0.39	100.2
3	3.78	10.83	0.93	0.32	44.60	0.51	38.84	n.d.	99.81
4	6.30	13.49	0.59	n.d.	46.09	n.d.	33.72	0.30	100.5
10	10.41	19.83	0.42	n.d.	40.66	0.52	27.8	n.d.	99.63
12	13.12	17.36	0.60	n.d.	50.32	0.53	18.63	n.d.	100.6
13	5.65	12.77	0.97	n.d.	38.88	0.50	41.55	n.d.	100.3
16	4.67	10.71	1.40	0.37	45.13	0.45	37.84	0.34	100.9
17	5.33	13.05	0.80	0.38	48.36	0.53	32.69	n.d.	101.2
21	4.99	7.64	0.36	n.d.	61.53	0.61	24.88	0.43	100.5
23	8.32	22.29	0.34	n.d.	41.43	n.d.	27.76	n.d.	100.2
26	10.84	17.75	0.24	n.d.	55.23	n.d.	15.58	0.35	99.97
27	5.36	12.27	1.21	0.35	33.20	0.48	47.78	n.d.	100.7
29	3.50	14.58	3.05	0.31	55.76	0.56	22.68	0.30	100.7
31	5.31	11.94	n.d.	n.d.	59.86	0.40	22.87	n.d.	100.4
33	13.68	34.83	0.29	n.d.	33.82	n.d.	17.30	n.d.	99.92
35	2.74	6.63	0.36	n.d.	22.81	n.d.	67.46	n.d.	100.0
36	2.84	13.19	n.d.	n.d.	50.18	0.45	33.51	0.58	100.8
39	5.47	17.04	0.36	n.d.	48.24	n.d.	29.24	0.25	100.6
40	7.02	11.18	n.d.	n.d.	59.40	n.d.	21.37	n.d.	98.97
44	0.24	7.96	0.76	n.d.	44.22	3.83	39.70	2.85	99.57
46	5.09	12.04	0.26	0.20	56.44	0.56	25.57	n.d.	100.2
47	9.69	21.17	n.d.	0.28	47.53	0.44	20.88	0.45	100.4
2	6.23	11.1	1.24	0.22	45.62	0.44	34.77	n.d.	99.61
4	5.91	13.65	1.22	0.24	43.93	n.d.	35.33	0.35	100.6
5	5.74	12.18	0.53	0.28	49.42	0.44	32.17	n.d.	100.8
6	10.19	15.41	0.21	n.d.	50.55	0.46	23.75	n.d.	100.6
9	4.97	10.38	0.28	n.d.	49.46	0.48	35.57	n.d.	101.1
10	4.82	14.28	1.21	n.d.	49.49	0.75	29.78	0.54	100.8
13	10.53	26.23	n.d.	0.21	40.60	0.43	22.66	n.d.	100.7
14	4.55	10.80	0.53	0.19	54.90	n.d.	29.10	n.d.	100.1
17	5.63	9.96	0.77	0.27	51.97	0.50	31.91	n.d.	101.0
18	5.42	13.77	0.38	0.27	50.07	0.54	30.29	0.34	101.1
20	3.71	14.02	0.38	n.d.	49.94	n.d.	33.40	n.d.	101.5
22	10.27	19.90	0.29	n.d.	45.67	n.d.	23.30	n.d.	99.42
25	6.86	15.63	0.69	0.20	44.44	0.48	32.29	n.d.	100.6
26	4.00	8.90	0.30	0.31	46.67	n.d.	40.60	0.43	101.2
27	14.24	34.78	n.d.	n.d.	27.90	n.d.	22.75	n.d.	99.67
28	6.71	13.80	0.59	0.41	56.50	0.65	21.27	0.39	100.3
29	8.05	12.42	n.d.	n.d.	53.11	n.d.	25.84	n.d.	99.42
33	3.54	5.11	n.d.	n.d.	42.95	0.65	48.30	n.d.	100.6

34	3.83	6.46	2.16	0.38	35.64	0.53	51.55	n.d.	100.6
36	3.70	8.22	0.53	0.19	59.15	0.51	27.35	n.d.	99.64
41	4.76	14.69	0.53	0.27	54.91	0.46	24.75	0.41	100.8
42	5.74	9.08	1.58	0.23	36.90	0.45	46.21	n.d.	100.2
43	5.52	24.83	n.d.	n.d.	44.09	n.d.	25.73	0.59	100.8
45	9.64	21.60	0.32	n.d.	40.1	0.48	28.19	n.d.	100.3
48	10.13	7.31	0.40	n.d.	59.86	0.74	22.50	n.d.	101.0
50	11.22	8.62	n.d.	n.d.	62.34	n.d.	17.37	1.10	100.6
53	7.77	18.95	2.82	0.36	39.26	0.41	31.29	0.35	101.2
54	7.54	13.10	0.88	0.23	53.49	0.59	24.50	n.d.	100.3
14	5.16	8.75	2.23	0.66	35.26	0.53	46.99	n.d.	99.58
7	6.00	16.37	0.74	0.51	28.36	0.53	47.29	n.d.	99.80
11	2.79	8.42	7.74	1.33	24.54	0.83	55.07	n.d.	100.7
14	1.77	4.27	4.31	0.65	22.51	0.65	65.43	n.d.	99.59
18	5.06	9.03	2.32	0.48	29.13	0.38	53.21	n.d.	99.61
22	8.45	17.71	2.36	0.74	28.19	0.40	42.70	n.d.	100.5
53	6.98	14.49	2.99	0.67	26.19	0.35	48.17	n.d.	99.84
46	3.63	10.84	2.94	0.86	43.71	0.61	38.40	0.49	101.5
51	7.76	22.28	0.69	0.60	15.13	0.27	53.67	n.d.	100.4
52	7.63	18.18	2.83	0.62	17.90	0.47	52.11	n.d.	99.73

-0.82 to -1.10 m

Weight 5.5 kg.

EC grains 32-63 μm

Grain	MgO	Al_2O_3	TiO_2	V_2O_3	Cr_2O_3	MnO	FeO	ZnO	Total
1	2.15	6.27	3.69	0.74	62.41	0.78	23.84	0.34	100.2
2	2.65	5.92	3.06	0.81	59.09	1.04	27.82	0.79	101.2
3	1.51	5.88	3.19	0.74	62.08	n.d.	26.17	n.d.	99.57
4	2.26	5.90	3.34	0.72	63.38	0.63	22.40	0.52	99.15
5	2.01	6.10	3.98	0.70	57.93	0.67	30.68	n.d.	102.1
6	2.37	6.24	2.64	0.88	59.34	1.16	28.72	n.d.	101.3
7	2.68	5.44	3.10	0.70	60.34	0.71	27.51	1.00	101.5
8	2.44	5.40	3.54	0.79	59.56	0.94	27.65	0.37	100.7
9	2.42	5.94	3.23	0.83	60.88	1.13	28.99	0.46	103.9
10	1.69	5.73	3.50	0.80	63.48	0.48	22.11	0.81	98.61
11	2.15	5.59	3.24	0.67	60.91	0.76	30.42	0.80	104.5
12	2.69	5.71	3.02	0.75	59.90	n.d.	28.48	n.d.	100.6

OtC-V grains 32-63 μm

Grain	MgO	Al_2O_3	TiO_2	V_2O_3	Cr_2O_3	MnO	FeO	ZnO	Total
1	3.80	11.43	0.57	0.55	55.41	n.d.	27.01	n.d.	98.76

OtC grains 32-63 μm

Grain	MgO	Al_2O_3	TiO_2	V_2O_3	Cr_2O_3	MnO	FeO	ZnO	Total
1	6.58	11.56	3.37	0.36	24.23	n.d.	53.02	n.d.	99.12
2	2.63	9.42	n.d.	n.d.	50.69	0.64	35.09	1.10	99.57
3	7.47	21.37	0.60	0.35	40.62	0.53	28.15	n.d.	99.09
4	6.35	17.05	1.19	0.42	42.15	n.d.	32.11	n.d.	99.26
5	22.18	9.52	n.d.	n.d.	40.29	0.84	26.85	n.d.	99.68

6	7.92	14.58	0.46	0.33	49.14	n.d.	27.66	n.d.	100.1
7	5.20	11.52	0.51	n.d.	54.55	n.d.	27.87	n.d.	99.66
8	13.51	34.93	2.02	n.d.	20.71	n.d.	29.28	n.d.	100.5
9	6.05	12.60	1.21	0.41	41.25	n.d.	38.71	n.d.	100.2
10	7.12	29.63	0.34	n.d.	35.20	n.d.	28.18	n.d.	100.5
11	6.37	11.07	0.94	0.42	37.55	0.51	43.08	n.d.	99.93
12	10.57	16.70	0.26	n.d.	51.10	0.14	21.88	n.d.	100.7
13	9.71	18.24	1.63	0.35	30.08	n.d.	39.29	n.d.	99.30
14	n.d.	6.86	n.d.	0.29	44.68	2.88	38.73	4.03	97.47
15	6.69	10.33	0.44	n.d.	58.27	n.d.	23.21	n.d.	98.93
16	3.42	10.53	1.29	0.37	30.19	n.d.	55.17	n.d.	101.0
17	9.14	17.66	0.35	n.d.	48.26	0.67	24.52	n.d.	100.6
18	9.96	16.55	2.02	0.37	37.39	n.d.	32.48	n.d.	98.77
19	6.30	16.44	2.72	0.56	40.13	n.d.	34.56	n.d.	100.7
20	4.78	13.96	1.52	0.46	32.92	0.40	45.51	n.d.	99.55
21	5.58	12.29	3.17	0.64	23.74	n.d.	53.88	n.d.	99.30
22	5.27	14.46	2.56	0.53	35.94	n.d.	41.65	n.d.	100.4
23	8.17	14.48	5.30	0.50	28.11	n.d.	43.31	n.d.	100.4
24	4.29	9.37	1.01	0.61	33.94	0.53	51.08	n.d.	100.8
25	5.73	11.68	4.26	0.59	32.57	0.46	44.28	n.d.	99.57
26	7.21	13.48	1.18	0.53	37.53	n.d.	39.53	n.d.	99.47
27	3.07	7.53	0.79	0.51	14.30	n.d.	72.58	n.d.	98.77
28	4.82	8.40	4.62	0.55	23.73	0.63	58.80	n.d.	101.5

-1.05 to - 1.35 m

Weight 101 kg.

EC grains >63 μm

Grain	MgO	Al_2O_3	TiO_2	V_2O_3	Cr_2O_3	MnO	FeO	ZnO	Total
2	3.69	6.52	2.29	0.73	58.93	1.06	25.39	1.30	99.90
4	3.62	6.39	2.44	0.62	58.24	1.09	25.74	0.81	98.95

OtC-V grains >63 μm

Grain	MgO	Al_2O_3	TiO_2	V_2O_3	Cr_2O_3	MnO	FeO	ZnO	Total
5	7.96	9.53	0.76	0.94	59.74	1.09	18.15	1.84	100.0
7	8.03	9.59	0.75	0.94	59.74	1.12	17.76	1.66	99.59
8	7.92	9.54	0.87	1.00	59.82	1.02	18.21	1.80	100.2
9	7.81	9.51	0.82	0.98	60.58	0.90	17.67	2.46	100.7
10	4.77	10.24	0.76	0.77	55.77	0.79	27.35	n.d.	100.5

OtC grains >63 μm

Grain	MgO	Al_2O_3	TiO_2	V_2O_3	Cr_2O_3	MnO	FeO	ZnO	Total
1	1.01	5.08	9.16	0.72	41.00	0.79	41.72	n.d.	99.48
3	16.01	42.77	n.d.	n.d.	25.88	n.d.	16.74	n.d.	101.4
6	9.87	16.93	n.d.	n.d.	52.20	0.54	20.40	n.d.	99.93

EC grains 32-63 μm

Grain	MgO	Al_2O_3	TiO_2	V_2O_3	Cr_2O_3	MnO	FeO	ZnO	Total
5	2.79	5.91	3.66	0.77	57.67	0.79	28.73	0.47	100.8
6	3.38	7.04	2.33	0.70	62.16	1.13	22.05	2.30	101.1
7	2.08	5.89	3.94	0.88	57.25	0.89	29.32	0.70	101.0
12	5.50	6.81	2.18	0.67	65.24	1.08	16.79	1.34	99.63
32	7.03	6.00	2.74	0.73	59.25	0.75	24.53	0.33	101.4
41	2.65	7.33	1.66	0.84	59.66	1.70	23.11	2.06	99.00
44	2.05	5.41	2.10	0.67	59.38	3.84	24.12	2.45	100.0
52	2.05	6.12	2.81	0.85	59.66	0.68	28.02	0.63	100.8
59	2.12	5.68	3.46	0.73	58.91	1.32	27.99	0.68	100.9
17	2.20	6.40	2.20	0.80	58.70	0.61	28.96	0.51	100.4
37	2.49	5.72	3.12	0.78	59.20	0.84	27.70	0.52	100.4
43	5.89	6.48	2.14	0.63	61.22	1.15	22.47	0.89	100.9
44	3.03	6.04	2.94	0.73	60.37	0.75	25.97	0.83	100.7
46	3.44	6.94	1.89	0.69	60.47	1.61	25.06	0.77	100.9
47	2.81	5.59	3.55	0.73	58.17	0.81	26.55	1.96	100.2
52	3.18	6.79	1.67	0.81	62.71	1.62	20.37	2.79	99.94
1	2.18	5.52	2.70	1.00	58.39	3.65	23.42	3.18	100.0
6	3.12	6.47	2.38	0.67	60.32	0.93	26.18	0.55	100.6
8	4.33	6.68	2.57	0.62	60.34	0.95	25.21	n.d.	100.5
9	2.21	6.40	2.96	0.77	58.92	0.90	27.65	0.44	100.3
14	3.34	6.43	2.34	0.74	61.38	0.96	25.30	0.86	101.4

OtC-V grains 32-63 µm

Grain	MgO	Al_2O_3	TiO_2	V_2O_3	Cr_2O_3	MnO	FeO	ZnO	Total
1	6.58	10.25	0.83	0.58	59.79	0.93	16.80	3.59	99.34
49	5.72	12.76	1.64	0.53	55.36	0.74	24.15	0.40	101.3
56	6.89	13.11	0.74	0.92	55.03	0.84	22.00	n.d.	99.54
36	3.46	10.34	0.47	0.91	58.36	1.14	25.08	0.98	100.7
38	5.05	8.53	0.98	0.72	61.05	0.82	23.16	n.d.	100.3
64	1.91	1.89	0.73	0.62	64.95	2.69	20.91	5.09	98.79
72	1.11	9.71	3.76	0.90	58.21	0.77	25.96	n.d.	100.4
10	4.44	14.70	2.39	0.66	50.30	0.65	26.98	n.d.	100.1
21	5.50	12.59	1.68	0.48	55.88	0.61	23.89	n.d.	100.6

OtC grains 32-63 µm

Grain	MgO	Al_2O_3	TiO_2	V_2O_3	Cr_2O_3	MnO	FeO	ZnO	Total
2	10.87	11.12	0.32	n.d.	54.89	0.56	22.21	n.d.	99.97
3	5.50	13.22	0.51	0.24	49.23	0.66	31.31	n.d.	100.7
4	8.80	26.37	1.32	0.40	24.52	n.d.	37.83	n.d.	99.23
8	10.69	19.84	0.80	0.34	39.44	0.47	27.74	n.d.	99.32
9	11.97	21.60	1.36	0.26	41.75	n.d.	22.27	n.d.	99.22
10	0.36	0.47	0.37	n.d.	42.37	1.94	51.77	1.35	98.65
11	10.30	22.08	0.62	n.d.	42.22	n.d.	25.30	n.d.	100.5
13	14.57	13.43	0.28	n.d.	55.77	0.43	16.17	n.d.	100.7
14	9.25	19.35	n.d.	n.d.	48.13	n.d.	21.86	n.d.	98.58
15	16.06	33.61	n.d.	n.d.	35.80	n.d.	15.15	n.d.	100.6
16	9.48	15.39	0.30	n.d.	50.95	0.47	23.15	n.d.	99.74
17	11.45	21.86	0.69	0.38	40.43	n.d.	24.52	n.d.	99.32
18	17.26	38.90	n.d.	n.d.	28.00	n.d.	15.59	n.d.	99.74
19	17.22	34.57	0.33	n.d.	32.26	n.d.	15.14	n.d.	99.53
20	8.53	16.67	0.32	n.d.	50.77	0.47	23.80	n.d.	100.6
21	10.46	29.34	0.58	n.d.	34.50	n.d.	25.15	n.d.	100.0
22	14.96	32.02	0.31	n.d.	35.15	n.d.	16.31	n.d.	98.75

24	10.05	18.75	0.35	n.d.	47.22	n.d.	23.25	n.d.	99.61
25	10.93	18.11	0.47	0.29	47.08	0.43	23.30	n.d.	100.6
26	7.75	13.43	0.94	n.d.	43.95	0.58	32.74	n.d.	99.39
27	5.63	13.81	1.13	0.31	57.63	0.78	21.83	0.37	101.5
28	11.34	21.47	0.46	0.19	42.75	n.d.	23.48	n.d.	99.70
29	9.28	17.95	0.49	0.27	46.02	n.d.	25.86	n.d.	99.87
30	9.69	19.16	0.69	0.32	42.38	n.d.	26.64	n.d.	98.88
31	2.23	10.44	1.36	n.d.	47.54	0.68	36.67	0.70	99.63
33	n.d.	1.16	0.70	n.d.	49.72	1.85	38.30	8.16	99.89
34	13.02	10.42	0.35	n.d.	58.03	n.d.	17.92	n.d.	99.74
35	10.34	21.34	0.62	0.28	37.85	n.d.	29.72	n.d.	100.2
37	7.65	16.00	0.79	0.43	52.70	0.70	21.38	n.d.	99.65
38	12.60	19.83	0.32	n.d.	46.91	0.44	21.56	n.d.	101.7
39	10.78	27.36	0.69	0.33	34.48	n.d.	26.41	n.d.	100.1
43	11.36	23.95	0.54	0.32	34.71	n.d.	27.83	n.d.	98.71
45	13.67	27.10	n.d.	n.d.	42.12	0.27	15.77	n.d.	98.93
46	9.86	22.11	0.33	n.d.	41.41	n.d.	26.36	n.d.	100.1
47	2.54	6.31	n.d.	n.d.	58.31	0.77	31.56	0.73	100.2
48	9.61	20.45	0.50	n.d.	43.32	n.d.	24.95	n.d.	98.83
50	7.07	13.87	2.72	0.43	44.16	0.58	30.76	n.d.	99.59
51	7.20	11.94	5.45	0.70	36.26	0.48	38.53	n.d.	100.5
53	8.38	25.24	1.23	n.d.	34.57	0.46	29.27	n.d.	99.16
54	16.15	28.41	0.28	n.d.	40.02	0.37	14.93	n.d.	100.2
55	7.97	11.01	0.22	n.d.	57.18	0.88	22.51	0.81	100.6
57	10.92	19.99	0.34	n.d.	44.11	n.d.	24.29	n.d.	99.65
58	16.18	36.35	0.52	n.d.	31.15	0.28	16.26	n.d.	100.8
60	9.17	15.43	0.75	n.d.	50.21	n.d.	24.27	n.d.	99.83
61	9.67	21.20	0.68	0.24	41.20	n.d.	26.74	n.d.	99.74
62	4.16	3.68	n.d.	n.d.	66.38	1.65	21.08	2.74	99.70
64	10.80	24.18	0.60	0.34	38.78	n.d.	25.16	n.d.	99.86
1	10.89	22.84	0.44	n.d.	42.46	n.d.	22.84	n.d.	99.48
2	4.60	17.75	1.11	0.35	38.03	0.50	38.08	0.33	100.8
3	7.04	18.98	0.37	0.28	43.80	n.d.	28.76	0.27	99.50
4	8.95	22.28	0.87	0.28	32.64	0.85	33.12	0.48	99.47
5	15.34	28.99	0.44	n.d.	38.34	0.39	17.24	n.d.	100.8
6	5.36	9.95	n.d.	n.d.	63.69	0.54	20.10	n.d.	99.63
7	5.71	10.24	n.d.	n.d.	63.87	n.d.	20.26	n.d.	100.1
8	11.13	21.74	0.55	0.23	39.60	n.d.	27.52	n.d.	100.8
9	4.18	19.38	0.67	n.d.	42.56	0.52	33.17	0.49	101.0
10	9.76	19.47	n.d.	0.25	54.85	0.41	15.82	n.d.	100.6
11	9.94	21.93	0.53	0.28	42.20	n.d.	25.28	n.d.	100.2
13	13.62	34.87	0.50	0.36	22.26	0.25	28.45	n.d.	100.3
15	4.33	12.89	0.66	n.d.	46.91	0.51	35.35	n.d.	100.7
16	4.27	21.53	0.68	n.d.	33.71	n.d.	39.85	0.49	100.5
18	5.65	14.02	0.46	0.31	48.11	0.51	30.66	0.50	100.2
19	10.08	33.32	n.d.	n.d.	34.11	0.45	20.88	0.53	99.36
20	9.09	4.13	n.d.	n.d.	63.90	1.02	20.04	1.41	99.58
21	8.38	21.43	1.64	0.30	31.95	0.46	35.49	n.d.	99.64
22	13.82	21.28	0.41	n.d.	45.34	n.d.	19.47	n.d.	100.2
23	5.43	14.47	0.46	n.d.	51.09	0.51	27.82	n.d.	99.77
24	6.89	16.99	0.17	0.29	48.14	0.43	27.18	0.32	100.4
25	9.29	19.98	0.66	0.31	40.81	n.d.	28.98	n.d.	100.0
27	15.62	36.95	0.52	n.d.	25.72	n.d.	21.40	n.d.	100.2
28	10.45	18.17	2.10	0.30	27.09	n.d.	41.91	n.d.	100.0
30	11.96	23.70	0.61	n.d.	41.05	0.40	21.99	n.d.	99.70
29	9.43	21.02	0.86	0.36	39.72	0.44	27.42	n.d.	99.24
31	8.89	14.34	0.99	n.d.	52.45	n.d.	23.14	n.d.	99.82
32	14.40	24.54	0.26	0.22	43.00	n.d.	18.97	n.d.	101.4

33	8.79	15.08	0.27	0.33	51.53	0.55	23.80	n.d.	100.3
34	10.65	20.77	0.47	0.36	41.81	n.d.	25.86	n.d.	99.92
35	10.56	23.67	0.71	0.33	39.69	0.41	25.04	n.d.	100.4
39	13.06	27.46	0.54	0.32	37.13	n.d.	22.23	n.d.	100.8
40	9.65	13.54	0.36	n.d.	54.12	0.46	21.48	n.d.	99.62
42	12.83	10.53	n.d.	n.d.	59.88	n.d.	16.69	n.d.	99.93
41	9.27	22.46	0.67	0.33	42.71	n.d.	25.21	n.d.	100.7
45	10.76	27.65	0.33	n.d.	33.65	n.d.	26.65	n.d.	99.03
48	10.78	21.28	0.76	0.36	35.65	0.49	31.13	n.d.	100.5
49	n.d.	4.70	0.63	n.d.	58.38	1.80	30.87	3.66	100.0
50	8.34	17.32	2.07	n.d.	44.22	n.d.	28.16	n.d.	100.1
51	13.79	35.95	1.13	n.d.	31.05	n.d.	18.93	n.d.	100.9
53	6.81	11.38	2.46	0.37	37.90	0.49	41.82	n.d.	101.2
54	6.26	13.57	0.62	0.39	48.64	n.d.	31.75	n.d.	101.2
55	1.30	11.55	1.05	0.38	47.72	0.60	37.91	0.55	101.1
56	6.68	13.24	n.d.	n.d.	57.74	0.45	21.96	0.43	100.5
57	9.83	10.88	0.25	n.d.	58.86	n.d.	20.38	n.d.	100.2
58	9.35	17.99	0.63	n.d.	46.01	n.d.	25.95	n.d.	99.92
59	11.49	21.35	0.39	n.d.	46.12	n.d.	21.30	n.d.	100.7
60	2.51	6.42	0.23	n.d.	60.75	0.53	29.76	0.40	100.6
61	7.71	12.17	0.62	n.d.	51.72	0.47	28.09	n.d.	100.8
62	10.53	21.62	0.39	0.21	45.63	0.50	22.43	n.d.	101.3
65	2.22	11.30	1.76	0.30	36.88	0.71	46.18	0.83	100.2
66	9.72	20.07	0.47	n.d.	48.12	0.46	22.53	n.d.	101.4
67	5.06	14.27	0.49	0.40	57.77	n.d.	21.86	n.d.	99.86
68	7.70	19.25	0.56	0.29	44.35	0.46	27.93	n.d.	100.5
69	8.98	12.88	0.41	n.d.	54.47	n.d.	22.86	n.d.	99.60
70	10.07	21.86	1.24	0.32	37.71	n.d.	28.69	n.d.	99.88
71	7.90	23.96	0.68	0.30	40.74	1.31	24.36	1.67	100.9
2	17.38	34.30	0.24	n.d.	33.31	n.d.	14.35	n.d.	99.58
3	15.58	22.28	0.15	0.15	47.30	0.34	14.29	n.d.	100.1
4	14.35	10.81	n.d.	n.d.	60.52	0.59	14.05	n.d.	100.3
5	18.61	41.48	n.d.	n.d.	24.86	n.d.	15.11	n.d.	100.1
7	8.79	17.48	0.58	n.d.	51.40	n.d.	21.75	n.d.	100.0
11	0.52	7.31	0.23	n.d.	45.27	7.33	31.95	7.18	99.77
12	6.89	20.65	1.08	0.32	35.20	0.51	35.25	n.d.	99.91
13	13.34	31.20	0.28	n.d.	35.94	0.36	19.14	n.d.	100.3
15	11.30	11.28	0.39	n.d.	54.94	0.60	21.76	n.d.	100.3
16	11.49	33.30	0.29	n.d.	35.81	n.d.	18.94	0.25	100.1
17	10.22	17.11	0.47	0.32	47.50	n.d.	25.28	n.d.	100.9
18	8.55	21.62	0.62	0.28	42.29	n.d.	26.47	n.d.	99.84
19	10.27	20.67	0.73	0.41	40.97	0.46	26.19	n.d.	99.70
20	7.67	8.81	0.27	n.d.	58.39	0.52	25.42	n.d.	101.1
23	7.55	31.36	n.d.	0.78	30.25	n.d.	29.48	n.d.	99.43
36	0.55	12.99	4.08	0.47	42.63	1.66	35.49	1.06	98.94
40	10.52	32.98	1.13	1.54	28.71	n.d.	24.82	n.d.	99.70
42	6.09	8.18	1.23	0.49	38.66	n.d.	44.71	n.d.	99.35
63	7.55	17.34	1.09	0.51	25.86	n.d.	46.83	n.d.	99.19
12	6.54	14.60	2.14	0.45	33.60	0.46	41.81	n.d.	99.59
14	3.73	7.70	2.06	0.50	44.61	n.d.	40.24	0.47	99.30
26	2.89	3.97	6.88	1.07	27.25	0.54	57.56	n.d.	100.2
63	5.04	30.82	1.45	0.49	36.06	0.49	24.73	0.81	99.91

-1.22 to - 1.42 m

Sample weight 103 kg.

EC grains >63 μm

Grain	MgO	Al_2O_3	TiO_2	V_2O_3	Cr_2O_3	MnO	FeO	ZnO	Total
1	6.20	6.03	3.33	0.75	61.20	0.78	22.32	0.46	101.7
2	4.92	6.85	2.33	0.77	59.13	0.95	23.94	0.94	99.82

OtC-V grains >63 μm

Grain	MgO	Al_2O_3	TiO_2	V_2O_3	Cr_2O_3	MnO	FeO	ZnO	Total
1	1.00	12.09	2.14	0.62	56.04	0.61	27.90	n.d.	100.4

OtC grains >63 μm

Grain	MgO	Al_2O_3	TiO_2	V_2O_3	Cr_2O_3	MnO	FeO	ZnO	Total
1	14.28	28.82	n.d.	n.d.	38.95	0.44	16.84	0.44	99.78
2	10.33	19.92	n.d.	n.d.	47.24	n.d.	21.99	n.d.	99.47

EC grains 32-63 μm

Grain	MgO	Al_2O_3	TiO_2	V_2O_3	Cr_2O_3	MnO	FeO	ZnO	Total
1	5.24	5.66	3.32	0.74	58.72	0.74	24.79	0.41	99.63
2	4.03	7.04	2.31	0.68	58.71	1.03	25.27	1.57	100.6
3	4.59	7.41	2.27	0.77	65.48	0.66	19.58	0.45	101.2
4	8.20	6.33	2.17	0.76	62.10	0.59	20.37	0.28	100.8
5	3.41	6.65	2.15	0.77	59.72	1.54	24.99	1.40	100.6
6	1.76	6.25	2.66	0.82	57.54	0.87	28.85	0.62	99.36
7	4.78	6.42	2.87	0.82	57.38	0.76	25.10	1.08	99.21
8	1.88	4.58	2.88	0.74	60.22	0.85	28.47	1.33	101.0
9	4.57	6.71	2.17	0.73	59.99	0.92	23.57	1.03	99.68
10	3.84	6.85	2.39	0.67	60.26	1.34	23.92	2.07	101.3
11	4.06	5.91	3.16	0.77	59.34	0.67	25.88	0.47	100.3
12	2.36	4.94	3.34	0.70	58.91	0.83	28.82	0.54	100.4
13	1.72	4.54	2.22	0.82	61.98	0.91	26.27	0.71	99.17
14	1.61	7.49	2.23	0.87	63.77	1.01	20.12	1.44	98.54
15	2.66	5.51	2.79	0.90	59.63	0.69	26.56	0.62	99.36
16	2.57	5.71	3.48	0.73	60.04	0.90	27.08	n.d.	100.5
17	4.76	4.72	2.14	0.83	61.78	1.12	24.41	1.13	100.9
18	1.77	6.49	3.70	0.78	56.98	0.65	26.50	3.09	99.97
19	4.20	6.52	1.84	0.83	60.32	n.d.	19.22	1.55	94.48
20	4.21	9.53	1.39	0.93	58.04	0.79	22.30	1.45	98.65

OtC-V grains 32-63 μm

Grain	MgO	Al_2O_3	TiO_2	V_2O_3	Cr_2O_3	MnO	FeO	ZnO	Total
1	4.78	13.12	0.49	0.52	55.28	n.d.	25.58	n.d.	99.78
2	8.59	15.15	1.01	0.51	54.17	0.72	20.15	0.29	100.6
3	2.79	12.11	1.48	0.53	57.25	0.70	23.66	n.d.	98.54
4	4.35	11.36	0.78	0.76	56.99	0.64	25.23	0.58	100.7
5	3.86	18.00	1.37	0.58	49.88	0.67	25.82	n.d.	100.2
6	1.44	9.55	3.85	0.53	51.25	0.73	31.28	n.d.	98.63
7	3.09	16.65	0.81	0.47	53.72	0.59	24.53	n.d.	99.85

OtC grains 32-63 μm

Grain	MgO	Al_2O_3	TiO_2	V_2O_3	Cr_2O_3	MnO	FeO	ZnO	Total
1	5.72	22.46	0.78	0.30	37.28	0.56	32.53	n.d.	99.62
2	7.23	18.33	0.65	0.37	43.15	0.42	29.92	n.d.	100.1
3	5.69	15.14	0.36	0.22	51.81	0.53	25.94	n.d.	99.69
4	12.32	26.02	3.03	n.d.	34.49	n.d.	23.67	n.d.	99.52
5	8.04	19.30	0.61	0.25	47.13	0.49	24.94	n.d.	100.8
6	7.73	10.11	n.d.	n.d.	63.95	n.d.	18.73	n.d.	100.5
7	10.86	20.49	0.68	0.28	39.83	n.d.	27.88	n.d.	100.0
8	7.59	14.06	0.27	n.d.	50.96	1.57	25.05	n.d.	99.50
9	4.62	12.08	0.41	n.d.	53.23	0.47	30.09	n.d.	100.9
10	8.65	9.13	0.26	n.d.	58.97	0.48	22.84	n.d.	100.3
11	9.19	21.97	0.59	0.30	39.63	0.50	27.83	n.d.	100.0
12	10.53	22.27	0.78	0.35	33.87	0.40	30.59	n.d.	98.80
13	3.66	14.12	0.46	0.27	47.73	n.d.	33.77	n.d.	100.0
14	9.07	15.17	0.41	n.d.	50.70	1.34	23.64	n.d.	100.3
15	10.70	24.11	0.78	0.30	36.23	n.d.	27.11	n.d.	99.23
16	4.50	13.14	0.66	0.39	48.69	0.51	32.19	n.d.	100.1
17	9.90	14.99	n.d.	n.d.	51.13	n.d.	23.64	n.d.	99.66
18	2.15	3.60	0.27	n.d.	18.46	n.d.	73.80	n.d.	98.28
19	11.57	20.13	3.60	0.30	31.90	0.44	31.26	n.d.	99.20
20	11.26	31.72	1.16	0.22	29.46	n.d.	26.50	n.d.	100.3
21	15.52	36.73	0.48	n.d.	31.44	n.d.	15.25	n.d.	99.43
22	2.58	10.67	0.66	n.d.	51.24	4.56	17.47	12.49	99.68
23	6.50	21.47	0.58	0.30	39.27	0.44	30.70	n.d.	99.26
24	9.19	22.65	0.81	0.40	35.58	0.37	31.78	n.d.	100.8
25	8.63	17.96	0.45	n.d.	45.87	n.d.	26.06	n.d.	98.98
26	10.70	22.68	0.62	0.25	39.76	n.d.	26.38	n.d.	100.4
27	10.28	19.50	1.34	0.26	42.35	n.d.	25.98	n.d.	99.71
28	8.22	18.88	n.d.	n.d.	44.53	0.63	26.87	n.d.	99.13
29	12.32	29.06	0.28	n.d.	40.00	n.d.	17.07	n.d.	98.73
30	11.11	15.09	0.49	n.d.	49.03	0.46	24.38	n.d.	100.6
31	16.59	33.83	0.21	n.d.	34.20	n.d.	14.47	n.d.	99.30
32	2.55	3.73	0.46	0.31	28.87	0.42	63.98	n.d.	100.3
33§	0.35	0.77	n.d.	n.d.	43.96	0.52	21.69	13.53	87.13
34	11.61	22.95	0.33	n.d.	41.82	0.40	22.77	n.d.	99.88
35	12.70	26.26	0.59	0.23	37.02	n.d.	24.12	n.d.	100.9
36	12.07	7.08	n.d.	n.d.	62.93	1.52	15.14	1.00	99.75
37	9.91	9.09	n.d.	n.d.	58.41	n.d.	23.01	n.d.	100.4
38	14.9	27.87	0.40	n.d.	40.49	n.d.	15.06	n.d.	98.73
39	5.35	13.62	1.01	n.d.	46.79	n.d.	33.05	n.d.	99.82
40	6.86	5.41	n.d.	n.d.	62.97	0.70	24.64	0.94	101.5
41	14.16	27.61	0.46	n.d.	38.51	n.d.	18.18	n.d.	98.92
42§	0.70	0.81	4.21	0.54	31.79	n.d.	11.89	23.83	84.93
43	10.72	21.52	n.d.	n.d.	52.09	0.57	14.82	0.63	100.3
44	10.10	19.76	0.55	n.d.	43.67	n.d.	24.78	n.d.	98.86
45§	n.d.	2.58	n.d.	n.d.	43.94	1.40	30.60	7.65	90.93
46	9.93	10.62	n.d.	0.21	58.24	n.d.	21.20	0.35	100.6
47	4.70	13.17	0.67	0.34	47.02	n.d.	32.69	0.38	98.97
48	13.59	14.71	0.27	n.d.	53.83	n.d.	17.97	n.d.	100.4
49	11.63	20.12	0.45	n.d.	47.61	n.d.	19.33	n.d.	99.13
50	0.51	13.32	0.52	n.d.	51.23	0.88	28.82	3.53	98.81
51	14.77	40.89	1.38	n.d.	16.75	n.d.	26.26	n.d.	100.1
52	12.41	23.03	0.47	0.29	41.64	n.d.	22.27	n.d.	100.1
53	9.89	9.68	n.d	n.d.	58.36	n.d.	21.21	0.29	99.43
54	17.35	37.48	0.30	n.d.	30.24	n.d.	15.89	n.d	101.3

55	11.19	10.25	0.24	n.d.	58.32	n.d.	20.23	0.36	100.6
56	10.66	15.95	0.42	n.d.	50.05	n.d.	21.67	n.d.	98.76
57	5.29	11.89	0.40	n.d.	60.58	n.d.	21.41	n.d.	99.56
58	10.82	19.80	0.31	n.d.	44.87	0.36	23.74	n.d.	99.91
59	15.84	31.41	0.38	n.d.	34.82	n.d.	17.24	n.d.	99.68
60	9.35	7.22	n.d.	n.d.	60.53	0.53	22.52	0.7	100.9
61	10.03	19.98	0.73	n.d.	41.50	n.d.	27.02	n.d.	99.27
62	10.88	22.96	0.46	n.d.	41.90	n.d.	22.56	n.d.	98.77
63	12.63	22.10	0.43	n.d.	44.22	n.d.	20.83	n.d.	100.2
64	10.09	10.78	0.17	n.d.	57.23	n.d.	22.08	n.d.	100.4
65	9.95	17.96	0.88	0.37	40.40	n.d.	31.17	n.d.	100.7
66	7.01	16.27	3.15	n.d.	39.27	0.55	32.77	n.d.	99.02
67§	0.38	1.08	0.25	0.32	45.55	0.94	19.36	13.97	84.82
68	13.14	34.14	n.d.	0.24	34.00	n.d.	19.16	n.d.	100.7
69	6.63	21.06	0.58	0.36	40.67	n.d.	30.02	n.d.	99.31
70	8.74	14.75	0.35	n.d.	51.05	0.71	24.09	n.d.	99.69
71	12.00	11.11	n.d.	n.d.	59.13	0.39	16.36	n.d.	99.00
72	8.45	15.02	0.37	0.26	50.37	n.d.	24.89	n.d.	99.36
73	7.04	10.35	n.d.	0.31	58.69	5.36	18.02	0.64	100.4
74	1.29	22.42	n.d.	n.d.	38.28	1.41	33.72	1.53	98.65
75	14.31	39.40	0.58	n.d.	27.93	n.d.	18.07	n.d.	100.3
76	8.19	25.07	1.04	n.d.	32.41	0.55	32.03	n.d.	99.29
77§	0.49	6.77	5.28	0.33	37.17	0.54	15.67	26.17	95.61
78	16.6	34.56	n.d.	n.d.	35.03	n.d.	14.04	n.d.	100.2
79	8.30	10.24	n.d.	n.d.	59.24	n.d.	22.63	0.40	100.8
80	8.24	22.59	0.78	n.d.	42.37	0.41	25.44	n.d.	99.83
81	9.37	20.16	0.72	0.35	40.77	n.d.	27.12	n.d.	98.49
82	15.46	36.09	n.d.	n.d.	36.39	n.d.	12.26	n.d.	100.2
83	7.01	28.18	0.44	n.d.	31.95	n.d.	30.86	0.54	98.97
84	1.50	16.93	1.95	0.47	29.83	0.63	46.81	1.24	99.34
85	5.68	18.12	1.68	0.47	33.75	1.24	37.04	1.12	99.10
86	8.00	13.19	2.70	0.54	38.77	0.32	35.72	n.d.	99.24
87	3.46	8.66	2.23	0.64	15.86	n.d.	69.23	n.d.	100.1
88'	3.23	5.50	5.34	0.65	25.43	0.53	56.85	0.95	98.98
89"	7.94	8.99	6.96	0.71	32.19	0.35	43.12	n.d.	100.7
90	5.13	9.19	1.29	0.46	34.41	0.53	49.43	0.47	100.9
91	5.85	6.38	2.80	0.55	35.81	0.52	48.20	n.d.	100.1
92	5.87	7.91	3.67	0.56	36.97	0.42	44.60	n.d.	99.99
93'''	6.65	13.81	2.64	0.50	42.82	0.45	33.88	n.d.	101.1

§Also SiO_2 and CaO, ' NiO 0.50 wt%, " NiO 0.48 wt%, ''' NiO 0.38 wt%.

-1.47 to -1.69 m

Sample weight 28.0 kg. Results for >63 µm fraction from Schmitz and Häggström (2006), but 32-63 µm fraction studied here.

EC grains >63 µm

Grain	MgO	Al_2O_3	TiO_2	V_2O_3	Cr_2O_3	MnO	FeO	ZnO	Total
1	4.06	5.48	3.33	0.72	58.83	1.04	25.27	1.14	99.87

OtC grains >63 µm

Grain	MgO	Al_2O_3	TiO_2	V_2O_3	Cr_2O_3	MnO	FeO	ZnO	Total

1	7.24	14.23	0.23	0.20	48.94	0.55	26.95	n.d.	98.15
2	8.25	16.87	0.51	0.24	47.54	0.53	24.18	n.d.	98.13
3	10.46	7.37	n.d.	n.d.	59.93	0.54	19.80	n.d.	98.18
4	5.11	14.29	0.14	0.14	53.28	0.58	23.98	0.43	97.84
5	4.72	13.69	0.46	0.28	43.01	0.52	35.39	n.d.	98.09
6	10.51	32.12	0.57	0.45	14.48	0.33	39.31	n.d.	97.78

EC grains 32-63 μm

Grain	MgO	Al_2O_3	TiO_2	V_2O_3	Cr_2O_3	MnO	FeO	ZnO	Total
1	0.80	6.35	2.20	0.79	57.83	n.d.	15.31	17.54	100.8
2	2.55	5.66	3.31	0.68	58.56	0.72	28.57	0.54	100.6
3	3.63	6.48	2.19	0.77	62.23	1.22	21.82	1.94	100.3
4	4.38	6.41	3.29	0.78	59.94	0.66	25.19	0.40	101.1
5	2.12	6.37	2.95	0.72	59.50	0.70	27.71	0.32	100.4
6	1.99	6.04	2.88	0.67	60.45	1.05	25.43	1.63	100.2
7	3.43	5.92	2.17	0.63	60.28	1.03	24.83	1.36	99.65
8	2.72	5.82	1.60	0.90	61.65	1.25	26.86	0.61	101.4
9	3.77	6.07	2.47	0.66	60.66	1.03	24.45	2.34	101.5
10	7.82	6.41	2.96	0.77	61.09	0.45	21.97	n.d.	101.5
11	6.23	4.69	3.40	0.75	61.51	n.d.	23.63	0.65	100.9

OtC-V grain 32-63 μm

Grain	MgO	Al_2O_3	TiO_2	V_2O_3	Cr_2O_3	MnO	FeO	ZnO	Total
1	5.14	6.20	1.05	0.51	59.98	n.d.	26.18	0.66	99.72

OtC grains 32-63 μm

Grain	MgO	Al_2O_3	TiO_2	V_2O_3	Cr_2O_3	MnO	FeO	ZnO	Total
1	10.73	16.24	0.31	n.d.	49.19	n.d.	23.51	n.d.	99.98
2	3.40	18.86	0.27	n.d.	50.36	n.d.	25.81	1.13	99.83
3	6.34	23.74	1.25	n.d.	33.50	n.d.	35.12	n.d.	99.93
4	10.14	15.94	2.11	0.43	31.06	0.56	39.77	n.d.	100.0
5	12.75	30.70	0.72	n.d.	35.68	n.d.	20.98	n.d.	100.8
6	9.25	14.78	n.d.	n.d.	53.55	n.d.	22.43	n.d.	100.0
7	0.29	9.92	n.d.	0.35	47.91	1.35	38.76	1.42	99.99
8	9.07	8.19	n.d.	n.d.	59.39	n.d.	23.23	n.d.	99.89
9	2.93	12.59	1.73	0.31	36.83	0.55	32.53	4.00	91.47
10	11.44	19.30	n.d.	n.d.	46.32	n.d.	24.60	n.d.	101.7
11	10.49	19.84	0.29	n.d.	47.79	n.d.	22.09	n.d.	100.5
12	7.54	11.22	0.34	n.d.	57.92	n.d.	21.68	n.d.	98.71
13	n.d.	13.70	0.43	n.d.	51.27	1.47	30.03	2.10	99.00
14	10.65	20.90	0.66	0.41	35.84	n.d.	31.08	n.d.	99.53
15	1.77	9.47	n.d.	n.d.	54.05	2.59	29.10	2.92	99.90
16	0.85	10.64	0.35	0.25	52.92	1.25	33.21	1.26	100.7
17	9.77	13.00	n.d.	n.d.	52.70	n.d.	24.50	n.d.	99.96
18	10.00	21.98	0.59	0.29	43.27	n.d.	24.45	n.d.	100.6
19	12.91	15.86	n.d.	n.d.	52.29	n.d.	19.80	n.d.	100.9
20	9.87	13.65	n.d.	0.28	52.35	n.d.	23.78	n.d.	99.92
21	4.25	11.02	0.49	n.d.	53.73	n.d.	30.90	n.d.	100.4
22	11.78	22.37	0.32	0.28	40.52	n.d.	24.92	n.d.	100.2
23	6.48	17.99	1.63	0.85	22.65	n.d.	49.61	n.d.	99.21
24	9.45	23.99	0.94	0.49	19.69	n.d.	46.39	n.d.	100.9
25	3.49	6.58	3.93	0.58	39.47	n.d.	45.23	0.75	100.1
26	7.84	15.15	2.10	0.60	31.79	n.d.	43.03	n.d.	100.5

-1.27 to -1.77 m

Sample weight 103.0 kg.

EC grains >63 µm

Grain	MgO	Al_2O_3	TiO_2	V_2O_3	Cr_2O_3	MnO	FeO	ZnO	Total
2	3.61	7.75	2.50	0.81	60.78	0.82	22.31	2.01	100.6

OtC-V grains >63 µm

Grain	MgO	Al_2O_3	TiO_2	V_2O_3	Cr_2O_3	MnO	FeO	ZnO	Total
1	6.48	14.15	0.90	0.48	54.24	0.80	21.84	0.69	99.59
8	5.72	23.79	0.72	0.67	42.33	0.42	26.16	n.d.	99.81

OtC grains >63 µm

Grain	MgO	Al_2O_3	TiO_2	V_2O_3	Cr_2O_3	MnO	FeO	ZnO	Total
3	9.94	8.82	n.d.	n.d.	58.58	0.43	21.45	n.d.	99.22
4	8.89	12.57	n.d.	n.d.	52.37	n.d.	25.35	n.d.	99.17
5	8.53	12.91	n.d.	n.d.	56.21	n.d.	21.91	n.d.	99.56
6	8.62	7.75	n.d.	n.d.	59.88	n.d.	24.57	n.d.	100.8
7	8.79	8.55	n.d.	n.d.	61.63	n.d.	21.71	0.40	101.1

EC grains 32-63 µm

Grain	MgO	Al_2O_3	TiO_2	V_2O_3	Cr_2O_3	MnO	FeO	ZnO	Total
10	3.44	6.58	2.42	0.74	59.78	1.44	26.01	1.01	101.4
13	3.56	6.44	2.41	0.69	59.43	1.15	26.45	0.77	100.9
16	4.17	5.81	3.64	0.80	58.78	0.71	25.97	0.57	100.5
18	1.96	5.79	4.26	0.78	57.12	n.d.	31.06	0.47	101.5
25	2.68	5.63	3.11	0.73	60.15	1.43	21.45	5.29	100.5
27	2.46	6.66	2.84	0.75	56.87	0.81	28.60	n.d.	98.99
31	2.52	5.71	4.00	0.67	58.47	0.84	28.33	0.70	101.3
33	2.41	5.85	3.69	0.62	56.69	0.97	27.87	1.08	99.18
38	3.57	7.10	1.92	0.68	60.05	1.01	26.08	1.17	101.6
7	2.37	6.23	3.04	0.81	60.96	0.82	25.35	n.d.	99.58
10	2.71	5.61	3.59	0.73	58.24	0.69	27.05	1.08	99.71
21	3.02	5.21	3.06	0.83	58.55	0.95	26.63	2.25	100.5
23	4.48	6.51	2.22	0.67	59.39	1.19	23.35	2.18	99.98
24	2.51	5.61	2.02	0.83	60.14	0.87	27.02	1.68	100.7
27	2.93	6.17	3.17	0.89	58.51	0.71	27.62	n.d.	100.0
45	3.87	6.24	2.41	0.86	60.10	0.68	26.09	0.60	100.9
4	1.90	5.78	3.06	0.68	58.61	0.98	29.49	0.88	101.4
5	3.50	6.36	2.41	0.68	60.33	1.12	26.24	0.76	101.4
8	2.42	5.78	3.93	0.75	59.15	0.76	26.38	0.66	99.83
12	2.26	6.12	3.78	0.68	59.63	0.74	25.22	0.57	99.00
3	6.26	5.90	3.40	0.76	59.68	0.63	23.68	0.45	100.8
8	2.21	5.81	3.72	0.81	59.31	0.72	26.15	1.09	99.82
12	3.13	5.32	3.79	0.68	58.67	0.85	27.20	0.91	100.6
13	2.91	6.96	2.52	0.77	59.68	1.19	25.40	1.14	100.6
18	1.89	5.93	3.99	0.78	60.33	0.77	26.23	0.62	100.5

21	2.41	5.52	3.59	0.68	61.96	0.62	24.76	0.44	99.97
22	2.81	5.43	4.22	0.70	60.43	0.82	25.78	0.73	100.9
27	6.84	5.43	3.46	0.72	59.95	0.79	23.39	n.d.	100.6
32	2.23	4.92	3.35	0.76	59.07	1.24	27.61	2.02	101.2
36	1.93	5.98	3.48	0.82	58.74	0.71	27.89	0.88	100.4
43	1.91	5.53	3.43	0.76	57.76	0.91	28.97	0.83	100.1
49	5.30	6.40	2.47	0.73	60.14	0.93	22.92	1.35	100.2

OtC-V grains 32-63 μm

Grain	MgO	Al_2O_3	TiO_2	V_2O_3	Cr_2O_3	MnO	FeO	ZnO	Total
23	3.49	5.59	0.62	0.51	61.46	1.95	24.67	2.73	101.0
29	5.79	8.85	0.71	0.57	60.52	1.76	20.25	1.88	100.3
42	5.13	21.48	0.65	0.54	45.94	0.45	25.39	n.d.	99.58
16	4.64	7.66	0.51	0.45	59.80	n.d.	26.85	0.58	100.5
18	3.61	15.04	0.97	0.52	52.73	0.70	26.94	n.d.	100.5
32	2.96	9.97	0.86	0.48	59.71	0.83	25.73	0.35	100.9
2	5.71	12.96	0.80	0.66	53.71	0.56	25.70	0.93	101.1
4	2.84	9.74	0.81	0.52	60.69	0.78	23.86	0.71	99.95
11	3.83	12.08	0.71	0.69	59.48	0.49	22.23	n.d.	99.51
42	3.73	11.77	1.23	0.52	57.56	0.66	24.85	0.97	101.3

OtC grains 32-63 μm

Grain	MgO	Al_2O_3	TiO_2	V_2O_3	Cr_2O_3	MnO	FeO	ZnO	Total
1	1.05	27.19	0.68	0.32	32.01	0.45	33.93	5.12	100.8
2	16.28	38.63	0.75	n.d.	22.72	n.d.	21.98	n.d.	100.4
5	8.59	14.54	0.79	0.30	41.51	n.d.	34.44	n.d.	100.2
6	n.d.	1.12	n.d.	0.30	43.55	0.93	30.30	5.48	81.67
7	12.24	15.24	0.32	n.d.	51.87	0.5	20.87	n.d.	101.1
9	13.23	27.95	1.80	n.d.	37.38	n.d.	20.15	n.d.	100.5
12	9.15	13.38	0.54	n.d.	49.59	n.d.	26.83	n.d.	99.49
14	13.33	25.99	0.56	n.d.	38.03	n.d.	22.20	n.d.	100.1
15	9.70	11.26	n.d.	0.18	54.67	n.d.	24.02	n.d.	99.83
19	7.94	9.38	n.d.	n.d.	58.30	n.d.	23.24	n.d.	98.87
20	9.43	13.73	n.d.	n.d.	52.99	n.d.	24.50	n.d.	100.7
21	11.19	27.80	2.48	0.35	22.95	0.28	33.69	n.d.	98.74
22	7.50	10.67	0.51	n.d.	53.16	n.d.	28.27	n.d.	100.1
24	6.14	30.05	0.47	n.d	23.41	0.44	39.10	0.53	100.1
26	11.59	18.79	0.34	n.d.	45.67	0.49	24.18	n.d.	101.1
28	2.79	12.19	n.d.	n.d.	50.65	1.12	30.64	2.65	100.0
32	4.62	14.81	0.94	0.38	36.50	0.63	41.81	n.d.	99.68
34	6.28	7.68	0.33	n.d.	58.01	0.56	27.23	n.d.	100.1
35	8.52	8.34	n.d.	n.d.	62.79	n.d.	20.77	n.d.	100.4
36	12.00	13.16	0.26	n.d.	54.59	0.36	20.34	n.d.	100.7
37	10.13	24.01	0.74	n.d.	30.53	n.d.	33.10	n.d.	98.51
39	11.12	11.60	n.d.	n.d.	56.36	0.63	20.16	n.d.	99.87
40	7.96	12.02	0.25	n.d.	52.56	0.68	26.18	n.d.	99.64
41	12.56	22.75	0.57	0.26	44.73	n.d.	18.76	n.d.	99.62
43	9.88	8.03	0.33	n.d.	59.39	0.52	21.53	n.d.	99.68
44	11.97	9.81	n.d.	n.d.	58.66	0.58	19.33	n.d.	100.4
1	6.54	24.07	n.d.	n.d.	39.09	0.50	29.09	n.d.	99.27
2	8.74	15.68	0.89	0.27	36.93	n.d.	37.43	n.d.	99.94
3	12.51	17.57	0.25	n.d.	47.41	n.d.	23.63	n.d.	101.4
4	4.61	15.45	0.34	n.d.	46.49	n.d.	32.47	0.55	99.91
5	11.58	10.13	0.28	n.d.	59.98	n.d.	18.63	n.d.	100.6

6	5.72	11.49	0.28	n.d.	61.94	3.45	16.44	0.91	100.2
8	12.01	15.14	n.d.	n.d.	51.26	0.45	20.29	n.d.	99.14
9	15.46	31.54	0.43	n.d.	32.30	n.d.	19.27	n.d.	99.01
11	10.92	19.14	0.35	n.d.	46.43	n.d.	23.68	n.d.	100.5
12	11.90	28.51	1.53	n.d.	32.93	n.d.	25.91	n.d.	100.8
13	11.13	12.43	0.48	n.d.	49.32	0.41	26.71	n.d.	100.5
14	8.02	16.31	0.4	n.d.	52.26	n.d.	22.59	n.d.	99.58
15	0.31	13.99	3.8	0.33	28.86	2.06	45.92	3.47	98.75
17	11.03	12.43	n.d.	n.d.	55.19	0.71	19.71	0.49	99.56
19	11.54	24.96	0.67	0.31	30.57	n.d.	30.55	n.d.	98.59
20	9.33	14.77	n.d.	n.d.	50.59	n.d.	25.64	n.d.	100.3
22	10.45	9.50	n.d.	n.d.	57.73	n.d.	21.60	n.d.	99.28
25	10.05	18.98	n.d.	n.d.	46.94	n.d.	23.25	n.d.	99.22
26	1.33	6.58	n.d.	n.d.	57.75	1.45	31.29	2.38	100.8
28	1.43	10.10	n.d.	n.d.	52.55	1.03	32.17	2.06	99.34
29	11.78	30.22	0.34	0.37	24.91	n.d.	31.82	n.d.	99.43
30	7.92	8.73	n.d.	n.d.	59.68	0.51	22.48	n.d.	99.32
31	3.75	7.58	2.56	0.27	41.19	0.59	45.00	n.d.	100.9
33	5.62	9.78	n.d.	n.d.	54.21	1.58	28.18	0.55	99.92
34§	0.61	3.73	2.14	0.31	40.27	0.80	32.34	8.21	89.60
35	0.53	0.41	4.27	n.d.	34.99	n.d.	12.90	21.8	74.91
37	9.16	14.85	n.d.	0.28	48.55	n.d.	26.75	n.d.	99.60
38	11.72	21.90	0.32	n.d.	44.09	n.d.	22.54	n.d.	100.6
39	6.16	36.43	n.d.	n.d.	22.19	0.32	32.05	2.45	99.60
40	10.12	20.07	0.33	n.d.	44.28	n.d.	24.37	n.d.	99.17
41	10.93	14.49	n.d.	n.d.	49.55	n.d.	25.57	n.d.	100.5
42	12.71	25.74	0.53	0.25	34.18	0.37	25.82	n.d.	99.59
43	7.41	7.38	n.d.	n.d.	58.33	n.d.	27.01	n.d.	100.1
44	7.74	11.43	n.d.	n.d.	54.59	n.d.	26.06	n.d.	99.82
46	7.04	11.81	0.61	n.d.	33.93	0.41	44.69	n.d.	98.50
47	12.58	15.87	n.d.	n.d.	51.63	0.37	20.21	0.18	100.9
2	n.d.	n.d.	n.d.	n.d.	84.98	n.d.	15.03	n.d.	100.0
3	7.51	11.66	n.d.	n.d.	55.44	0.69	25.81	n.d.	101.1
6'	21.96	16.93	0.74	n.d.	20.37	0.61	38.72	n.d.	99.90
7	5.16	18.54	n.d.	n.d.	48.70	0.50	26.52	n.d.	99.43
9	5.60	12.70	1.60	n.d.	45.31	n.d.	33.90	n.d.	99.10
10	11.74	30.08	0.88	n.d.	34.58	n.d.	22.55	n.d.	99.83
11	11.74	30.08	0.88	n.d.	34.58	n.d.	22.55	n.d.	99.83
13	7.17	18.04	0.65	0.31	37.07	n.d.	36.73	n.d.	99.97
1	11.88	33.18	1.07	0.35	16.87	n.d.	35.22	n.d.	98.57
5"	8.29	10.27	n.d.	n.d.	56.50	0.78	25.24	n.d.	101.5
6	7.71	14.34	n.d.	n.d.	54.64	n.d.	22.48	n.d.	99.17
7	10.19	12.42	n.d.	0.26	53.44	n.d.	24.51	n.d.	100.8
9	6.62	13.52	0.32	n.d.	59.51	0.76	19.21	0.88	100.8
14	10.21	21.83	0.88	0.33	30.49	n.d.	35.61	n.d.	99.34
15	5.09	25.45	0.45	n.d.	35.54	0.52	30.86	0.85	98.76
16	8.29	10.85	3.01	0.34	40.98	0.50	36.58	n.d.	100.6
17	9.27	18.17	n.d.	n.d.	48.91	n.d.	23.70	n.d.	100.0
19	2.45	0.56	n.d.	n.d.	43.63	1.72	50.17	0.85	99.38
20	9.83	25.68	1.06	0.34	23.58	0.41	39.32	n.d.	100.2
23	0.89	17.36	0.32	n.d.	46.66	1.36	30.35	2.65	99.59
24	10.88	22.25	0.85	0.29	32.57	n.d.	33.81	n.d.	100.6
25	2.76	14.34	n.d.	0.27	40.74	2.30	34.31	5.71	100.4
26	9.05	9.63	n.d.	n.d.	59.88	n.d.	21.32	99.87	99.87
28	14.2	11.72	n.d.	n.d.	59.26	0.49	13.27	n.d.	98.95
29	4.47	18.55	0.55	n.d.	42.78	n.d.	31.06	0.76	99.16
30	7.38	9.58	0.65	n.d.	57.80	0.65	22.80	0.55	99.41
31	16.17	40.11	0.35	0.21	24.56	n.d.	18.65	n.d.	100.1

33	7.77	16.53	5.16	0.30	28.50	0.43	40.44	n.d.	99.13
34	12.78	27.37	n.d.	n.d.	38.48	n.d.	21.86	n.d.	100.5
35	10.04	27.32	0.29	0.22	39.66	n.d.	21.47	n.d.	99.00
37	11.74	22.43	n.d.	0.23	45.30	0.50	19.84	n.d.	100.0
38	7.82	23.09	2.42	0.28	33.58	n.d.	32.08	n.d.	99.29
39	3.93	7.00	0.37	0.40	58.79	0.64	29.21	n.d.	100.3
40	10.20	9.62	n.d.	n.d.	58.67	0.62	21.50	0.52	101.1
41	2.24	12.11	n.d.	n.d.	49.94	1.82	31.76	2.37	100.2
44	10.57	9.34	n.d.	n.d.	57.04	0.45	21.08	n.d.	98.48
45	8.37	21.49	n.d.	n.d.	46.03	n.d.	22.93	0.38	99.20
46	11.43	9.86	n.d.	n.d.	58.38	0.65	20.05	n.d.	100.4
47	9.18	9.10	0.25	n.d.	58.78	0.68	21.32	1.41	100.7
48	2.62	19.04	0.58	n.d.	41.40	1.51	31.10	4.36	100.6
50	12.95	33.78	0.66	0.33	29.73	n.d.	21.63	n.d.	99.09
51	9.27	14.64	0.25	n.d.	51.26	0.31	23.84	n.d.	99.57
52	6.50	13.17	0.25	n.d.	53.82	0.46	25.75	n.d.	99.96
3	9.52	23.56	0.69	0.50	23.91	n.d.	40.62	n.d.	98.80
4	9.79	23.33	1.55	0.52	23.07	n.d.	42.66	n.d.	100.9
8	7.22	20.46	2.10	0.59	32.94	0.49	36.70	n.d.	100.5
11	n.d.	5.73	2.85	3.94	32.95	n.d.	51.63	1.24	98.34
17	9.75	24.31	0.59	0.47	23.78	0.39	39.63	n.d.	98.92
30	8.56	20.75	1.19	0.60	20.59	0.26	47.67	n.d.	99.63
36	10.04	24.84	0.56	0.47	24.36	n.d.	39.27	n.d.	99.54
1	6.05	11.05	2.58	0.58	33.48	n.d.	46.02	n.d.	99.76
10	7.96	20.37	0.87	0.54	27.05	n.d.	42.35	n.d.	99.13

[§]Also SiO_2 1.18 wt%, ' NiO 0.58 wt%, " CuO 0.41 wt%.

-1.97 to -2.12 m

Sample weight 26.0 kg.

EC grains 32-63 µm

Grain	MgO	Al_2O_3	TiO_2	V_2O_3	Cr_2O_3	MnO	FeO	ZnO	Total
1	4.67	5.69	3.79	0.67	60.40	0.31	22.91	0.30	98.74
6	3.59	5.72	2.53	0.63	59.52	0.96	23.70	2.86	99.50
9	4.00	5.60	3.47	0.74	58.21	0.46	26.97	0.30	99.74
15	2.14	5.69	3.45	0.62	57.82	0.35	26.95	1.62	98.65
17	4.54	6.31	2.27	0.71	59.68	0.80	24.51	0.78	99.61
21	3.92	6.51	2.36	0.65	58.64	0.89	25.05	1.24	99.24
29	4.95	5.49	3.41	0.62	58.78	0.38	26.42	0.24	100.3
43	5.31	5.56	3.85	0.70	58.66	0.38	25.59	0.25	100.3
50	3.26	6.51	2.34	0.66	58.11	0.62	27.15	0.61	99.27
53	4.62	6.07	3.70	0.68	62.56	0.18	16.67	0.14	94.61
61	2.59	5.22	3.04	0.61	61.23	0.71	24.67	0.55	98.61
90	1.95	6.00	4.11	0.58	58.93	0.31	23.07	0.67	95.62
94	2.43	6.67	2.35	0.66	62.64	0.54	20.50	0.39	96.18
140	3.26	5.72	3.18	0.63	62.16	0.42	22.74	0.36	98.48

OtC-V grains 32-63 µm

Grain	MgO	Al_2O_3	TiO_2	V_2O_3	Cr_2O_3	MnO	FeO	ZnO	Total
103	3.75	10.61	1.08	0.52	59.93	0.23	21.50	0.14	97.75

OtC grains 32-63 μm

Grain	MgO	Al_2O_3	TiO_2	V_2O_3	Cr_2O_3	MnO	FeO	ZnO	Total
4	9.27	18.37	2.24	0.40	27.75	0.23	38.78	0.10	97.14
7	9.02	15.64	0.27	0.09	49.20	0.19	23.88	0.18	98.46
10	8.25	17.90	1.18	0.34	25.28	0.25	42.75	0.11	96.06
11	8.69	16.72	1.06	0.20	39.17	0.13	30.25	0.20	96.42
13	9.04	14.25	0.88	0.30	35.75	0.21	36.26	0.07	96.75
16	8.23	16.17	0.18	0.53	52.05	0.12	20.26	0.17	97.71
19	7.05	22.44	2.82	0.37	20.77	0.22	43.25	0.13	97.04
23	11.23	31.83	0.78	0.20	30.83	0.11	22.02	0.11	97.13
24	13.39	17.57	0.06	0.23	55.12	0.27	12.02	0.35	99.00
25	9.66	13.15	0.33	0.12	51.15	0.17	22.83	0.09	97.50
26	7.26	10.04	0.17	0.08	53.40	0.21	27.25	0.07	98.47
27	5.30	11.07	0.65	0.42	57.77	0.54	23.26	0.80	99.82
28	9.17	20.11	1.07	0.35	26.50	0.22	39.48	0.05	96.93
32	8.90	21.92	2.19	0.27	37.49	0.12	25.03	0.56	96.47
34	9.71	22.03	1.65	0.35	33.38	0.16	30.56	0.14	97.97
35	9.40	26.16	0.21	0.17	38.13	0.16	22.56	0.18	96.97
37	12.34	24.86	0.44	0.21	41.95	0.06	18.09	0.04	97.97
39	10.36	16.17	1.66	0.28	32.23	0.34	36.11	0.17	97.32
40	9.82	22.08	0.57	0.15	46.58	0.02	15.89	0.17	95.27
41	11.37	38.69	1.26	0.16	19.46	0.11	21.91	0.04	93.00
44	11.84	21.08	0.30	0.07	46.54	0.27	15.73	0.10	95.92
45	0.41	0.72	1.84	0.13	31.78	0.73	57.09	0.51	93.23
47	8.40	24.07	1.29	0.26	30.23	0.54	28.67	0.44	93.91
48	7.68	14.48	0.37	0.13	47.22	0.15	26.00	0.14	96.16
54	9.69	27.46	0.67	0.16	35.75	0.11	21.82	0.14	95.80
55	9.88	16.43	0.54	0.21	46.83	0.12	23.39	0.07	97.47
56	8.47	11.19	0.20	0.14	52.96	0.22	25.20	0.10	98.47
57	14.43	26.78	0.44	0.20	38.87	0.11	16.09	0.06	97.00
59	6.79	16.31	0.35	0.11	52.20	0.36	19.45	0.16	95.73
60	6.92	7.14	0.14	0.10	58.89	0.26	25.48	0.31	99.24
62	13.35	23.65	3.92	0.18	24.45	0.16	31.55	0.04	97.30
63	10.43	22.03	0.54	0.24	42.19	0.11	21.20	0.11	96.85
65	9.37	20.52	0.83	0.30	27.54	0.17	38.28	0.15	97.17
66	9.25	33.19	0.43	0.14	18.85	0.24	34.50	0.34	96.93
67	5.37	3.89	0.04	0.14	65.91	1.03	21.57	2.79	100.8
68	4.66	21.76	0.38	0.42	41.05	0.19	28.68	0.13	97.28
70	11.60	24.31	0.53	0.16	42.16	0.08	18.11	0.08	97.05
72	0.17	1.92	5.33	0.42	35.22	0.04	19.00	22.95	85.04
74	8.34	7.28	0.19	0.12	59.24	0.22	23.08	0.17	98.65
76	9.74	10.19	0.19	0.15	54.30	0.16	23.13	0.07	97.93
81	6.28	10.30	0.22	0.12	55.93	0.10	22.12	0.10	95.17
82	0.39	13.16	1.19	0.68	41.77	0.17	37.43	1.58	96.36
83	8.47	7.10	0.18	0.13	56.60	0.16	26.20	0.20	99.03
84	1.84	0.88	0.53	0.18	45.97	0.30	45.17	0.31	95.18
86	8.48	13.84	2.76	0.34	34.42	0.15	36.40	0.18	96.55
87	5.45	13.45	0.61	0.42	57.54	0.16	17.98	0.01	95.63
88	13.73	23.09	0.66	0.22	41.80	0.11	18.38	0.05	98.03
93	9.28	20.03	1.08	0.33	26.23	0.21	39.65	0.10	96.90
96	6.71	11.65	0.19	0.16	50.99	0.34	28.25	0.22	98.50
98	7.43	25.53	0.76	0.15	31.04	0.15	30.27	0.17	95.49
99	7.04	14.11	2.33	0.33	33.28	0.25	40.11	0.20	97.65
107	0.39	0.18	0.02	0.15	26.17	0.06	32.47	0.19	59.64

-2.12 to -2.30 m

Sample weight 32.0 kg**.**

EC grains >63 µm

Grain	MgO	Al_2O_3	TiO_2	V_2O_3	Cr_2O_3	MnO	FeO	ZnO	Total
2	2.36	5.74	4.09	0.71	57.24	0.73	28.30	0.53	99.70

OtC grains >63 µm

Grain	MgO	Al_2O_3	TiO_2	V_2O_3	Cr_2O_3	MnO	FeO	ZnO	Total
1	12.48	9.14	n.d.	n.d.	60.35	n.d.	17.55	n.d.	99.52

EC grains 32-63 µm

Grain	MgO	Al_2O_3	TiO_2	V_2O_3	Cr_2O_3	MnO	FeO	ZnO	Total
1	2.45	5.99	2.07	0.75	60.32	1.12	26.59	0.52	99.81
3	1.93	5.69	3.43	0.81	57.80	0.74	27.44	1.68	99.53
4	3.99	6.75	2.46	0.76	59.13	0.73	23.35	2.02	99.20
5	3.73	6.66	2.48	0.69	58.54	1.09	25.13	0.78	99.10
7	1.52	6.30	3.41	0.62	62.60	0.64	24.24	0.39	99.72
8	1.99	5.61	3.31	0.79	58.26	0.78	28.78	0.71	100.2
9	3.79	6.80	1.89	0.69	50.09	0.99	25.74	0.51	99.50
10	3.83	5.94	3.58	0.66	57.28	0.57	26.62	0.64	99.12
11	3.62	5.46	2.61	0.78	64.56	0.63	21.71	0.99	100.4
12	4.44	5.80	1.31	0.80	62.20	1.31	21.89	2.72	100.5
13	3.86	6.14	1.67	0.86	60.77	1.11	24.91	1.28	100.6
16	3.67	6.86	2.46	0.73	61.69	0.90	21.84	0.64	98.79
17	2.72	5.99	2.60	0.66	58.23	0.75	27.67	0.83	99.45
18	2.17	6.28	3.16	0.65	62.71	0.61	23.63	0.91	100.1
19	3.64	5.87	2.59	0.81	59.06	0.83	25.36	0.67	98.83
20	2.41	5.39	2.53	0.69	59.93	1.15	24.14	4.12	100.4
21	3.85	6.59	2.39	0.72	60.02	1.26	25.20	0.74	100.8
22	3.46	5.41	3.68	0.70	57.89	0.83	26.99	0.53	99.49
23	3.47	6.25	3.25	0.74	59.39	0.67	25.72	n.d.	99.49
24	3.21	8.10	3.72	0.55	54.97	0.65	28.08	n.d.	99.27
25	7.11	8.77	1.87	0.66	60.16	0.78	19.60	2.08	101.0
27	2.55	5.55	2.76	0.73	59.43	0.81	28.66	0.51	101.0
29	2.71	6.23	2.92	0.75	59.66	0.85	26.48	1.05	100.7
30	3.32	6.39	3.26	0.79	59.99	0.89	24.49	n.d.	99.13
2	2.45	6.03	2.91	0.81	58.86	0.80	27.99	1.01	100.6
28	2.85	6.87	2.36	0.76	60.25	0.87	26.56	0.51	101.3

OtC-V grains 32-63 µm

Grain	MgO	Al_2O_3	TiO_2	V_2O_3	Cr_2O_3	MnO	FeO	ZnO	Total
15	6.94	5.92	0.47	0.69	62.22	0.89	16.15	7.77	101.7
26	7.84	17.55	1.55	0.45	42.65	n.d.	29.08	0.32	99.44
28	5.58	9.41	0.83	0.62	58.41	1.05	22.83	1.61	100.4
21	n.d	10.23	n.d.	0.63	55.96	2.59	24.66	7.04	101.1
34	4.50	21.07	0.67	0.55	44.98	n.d.	27.03	n.d.	98.81

OtC grains 32-63 μm

Grain	MgO	Al_2O_3	TiO_2	V_2O_3	Cr_2O_3	MnO	FeO	ZnO	Total
1	12.42	27.70	0.40	n.d.	42.29	n.d.	17.11	n.d.	99.91
3	7.28	23.05	0.54	n.d.	43.73	0.65	22.10	0.84	98.18
4	12.48	28.19	1.76	n.d.	26.39	n.d.	29.78	n.d.	98.59
5	9.32	21.93	0.91	n.d.	42.15	n.d.	26.76	n.d.	101.1
6	10.37	20.33	0.41	n.d.	46.20	n.d.	23.56	n.d.	100.9
7	9.22	6.12	n.d.	n.d.	64.39	n.d.	22.24	n.d.	102.0
8	11.02	32.01	1.16	0.30	30.43	n.d.	23.39	n.d.	98.30
10	9.04	23.80	1.48	n.d.	43.66	n.d.	20.58	n.d.	98.57
11[§]	0.64	2.82	2.65	n.d.	41.79	n.d.	28.19	13.42	96.01
12	5.86	12.23	n.d.	n.d.	51.84	n.d.	31.04	n.d.	101.0
13[§]	n.d.	0.65	n.d.	n.d.	43.29	1.44	30.46	5.48	86.02
14	19.33	18.61	n.d.	n.d.	20.04	1.09	39.67	n.d.	98.74
15	12.57	24.78	n.d.	n.d.	43.16	n.d.	17.52	n.d.	98.03
16	8.48	28.43	0.52	n.d.	35.59	n.d.	26.12	n.d.	99.13
17	11.81	24.72	0.62	n.d.	43.55	n.d.	17.95	n.d.	98.65
18	9.70	24.73	n.d.	n.d.	31.13	n.d.	32.62	n.d.	98.17
20	10.10	37.91	0.83	n.d.	28.32	n.d.	24.12	n.d.	101.3
22	8.52	12.32	n.d.	n.d.	53.57	n.d.	24.38	n.d.	98.79
23	4.90	14.68	n.d.	n.d.	49.47	n.d.	29.86	n.d.	98.92
24[§]	n.d.	1.67	0.83	n.d.	36.18	n.d.	16.05	16.09	77.81
25	14.00	37.66	n.d.	n.d.	32.29	n.d.	15.66	n.d.	99.61
26[§]	5.41	14.62	3.81	n.d.	27.70	n.d.	34.43	1.58	91.56
27	7.60	13.55	n.d.	n.d	55.77	n.d.	24.54	n.d.	101.5
29	1.74	10.81	0.54	n.d.	59.35	n.d.	19.42	6.91	98.76
30	8.71	0.95	n.d.	n.d.	52.55	2.00	31.08	3.61	98.99
31	13.57	36.24	n.d.	n.d.	33.49	n.d.	18.12	n.d.	101.4
32	13.07	31.01	n.d.	n.d.	37.81	n.d.	17.79	n.d.	99.68
33	1.96	1.28	n.d.	n.d.	60.26	n.d.	35.24	n.d.	98.75
35[§]	0.35	2.73	4.36	0.38	37.05	n.d.	15.76	25.11	89.66
36	11.19	31.48	1.91	n.d.	32.43	n.d.	22.72	n.d.	99.73
37	13.71	3.53	n.d.	n.d.	32.32	n.d.	49.32	n.d.	98.89
38	n.d.	6.97	n.d.	n.d.	52.28	2.61	36.43	2.72	101.0
39	10.59	10.23	n.d.	n.d.	56.67	n.d.	23.84	n.d.	101.3
40	12.96	20.17	n.d.	n.d.	46.59	n.d.	19.09	n.d.	98.82
41[§]	0.55	1.91	0.80	n.d.	40.76	0.86	26.17	9.35	85.69
42	7.84	5.49	n.d.	n.d.	63.46	n.d.	18.74	2.69	98.22
43	9.91	32.20	n.d.	n.d.	38.02	n.d.	20.86	n.d.	101.0
44[§]	1.36	10.00	0.83	n.d.	38.04	n.d.	42.95	2.52	97.12
45	3.01	35.75	n.d.	n.d.	20.44	n.d.	34.62	5.92	99.75
46[§]	n.d.	0.52	n.d.	n.d.	44.90	1.87	32.41	7.58	91.95
47	14.34	30.54	n.d.	n.d.	38.72	n.d.	16.45	n.d.	100.1
48	3.06	12.57	n.d.	n.d.	54.90	n.d.	25.80	1.77	98.10
49	1.74	19.47	0.67	n.d.	42.32	2.12	29.97	2.56	98.85
50	10.87	25.83	0.51	n.d.	43.10	n.d.	18.97	n.d.	99.28
51	10.54	25.12	1.17	n.d.	43.55	n.d.	20.52	n.d.	100.9
52	5.74	8.59	n.d.	n.d.	52.71	1.42	31.26	n.d.	99.71
2	3.82	18.77	0.56	0.45	41.87	0.44	32.41	0.58	98.92
6	2.43	5.85	4.49	0.68	32.22	0.46	52.98	n.d.	99.11
14	n.d.	4.42	0.27	0.36	44.18	2.53	44.53	3.15	99.43
9	7.29	10.83	3.47	0.48	21.50	0.47	55.04	n.d.	99.09
19	2.06	6.69	5.84	0.68	33.57	n.d.	50.39	0.78	100.0

[§]Grains with unusual composition including also SiO_2 in the range 1.42-6.11 wt%, and in some cases <1 wt% Ca, P or K.

-2.35 to -2.60 m

Weight 31.7 kg. From Schmitz and Häggström (2006). Only searched for grains in >63 µm fraction, no chrome spinel grains found.

-2.60 to -2.85 m

Weight 30.2 kg. From Schmitz and Häggström (2006). Only searched for grains in >63 µm fraction, no chrome spinel grains found.

-2.60 to 3.10 m

Weight 104 kg.

In addition to the grains reported below, this sample contained 289 Fe-Cr grains with the approximate composition: 39 wt% FeO, 18 wt% Cr_2O_3, 28 wt% Al_2O_3, 12 wt% MgO, and 1.5 wt% TiO_2. These grains were only analysed semi-quantitatively in an unpolished state, and the results are not included here.

EC grains >63 µm

Grain	MgO	Al_2O_3	TiO_2	V_2O_3	Cr_2O_3	MnO	FeO	ZnO	Total
2	2.35	6.37	3.74	0.67	63.34	0.73	21.77	0.39	99.35
4	3.88	5.73	4.39	0.71	56.46	0.73	25.33	1.85	99.10
5	2.75	6.40	3.83	0.82	59.98	0.63	23.86	0.52	98.78
8	2.16	6.61	4.46	0.75	57.66	0.47	25.55	1.70	99.36

OtC-V grains >63 µm

Grain	MgO	Al_2O_3	TiO_2	V_2O_3	Cr_2O_3	MnO	FeO	ZnO	Total
1	6.13	8.80	0.77	0.80	58.59	0.94	18.23	4.23	98.49
3	4.34	15.30	1.17	0.53	52.33	0.57	26.46	0.28	101.0
6	8.06	10.82	1.50	0.68	58.70	0.97	17.22	1.01	98.96
7	4.12	14.55	4.30	0.76	52.32	0.53	22.63	0.37	99.58

EC grains 32-63 µm

Grain	MgO	Al_2O_3	TiO_2	V_2O_3	Cr_2O_3	MnO	FeO	ZnO	Total
1	2.20	6.12	4.30	0.82	58.72	0.72	26.09	n.d.	98.97
2	2.45	6.37	3.15	0.75	59.26	0.86	27.52	n.d.	100.4
3	3.69	5.79	3.64	0.72	58.57	0.64	27.97	n.d.	101.0
4	2.51	6.05	3.62	0.85	57.22	0.83	27.90	1.64	100.6
5	3.24	5.92	3.23	0.91	58.52	0.73	26.50	0.56	99.62
6	2.61	5.60	3.31	0.79	62.29	0.78	23.89	0.45	99.71
7	4.21	7.15	2.71	0.69	59.37	1.05	25.54	0.66	101.4
8	1.64	6.38	2.99	0.75	60.82	1.22	26.28	0.72	100.8
9	3.30	7.54	1.73	0.83	62.61	0.74	20.35	1.76	98.87

10	3.07	5.18	3.20	0.62	60.87	0.74	25.34	n.d.	99.03
11	3.15	5.45	3.03	0.72	59.65	0.81	25.69	1.54	100.0
12	7.99	6.37	3.27	0.65	58.25	0.52	23.34	n.d.	100.4
13	3.74	6.93	2.00	0.69	59.44	0.96	25.78	0.44	99.97
14	3.32	5.92	2.69	0.71	58.36	1.01	26.32	0.79	99.12
15	1.72	7.32	2.96	0.91	62.38	0.68	22.65	1.09	99.71
16	3.22	4.26	1.23	0.73	62.86	1.64	22.06	3.44	99.45
17	3.27	6.55	2.40	0.84	58.09	1.23	25.37	1.06	98.82
18	3.99	6.89	2.61	0.70	59.83	1.08	24.76	0.68	100.6
19	2.90	5.64	4.00	0.80	62.33	0.90	21.12	1.74	99.43
20	1.94	5.99	3.51	0.75	59.32	0.72	26.46	0.69	99.39
21	2.24	6.11	3.44	0.78	57.72	0.70	29.29	0.43	100.7
22	2.85	6.25	3.20	0.75	58.87	0.72	26.90	n.d.	99.54
23	1.76	6.25	2.60	0.78	57.30	0.78	28.78	0.89	99.13
24	3.87	5.92	2.98	0.84	58.58	0.70	26.97	n.d.	99.86
25	2.62	5.94	3.21	0.81	58.03	0.99	27.39	1.60	100.6
26	3.00	7.21	2.35	0.84	61.09	1.26	24.51	n.d.	100.3
27	3.92	6.56	2.52	0.65	58.69	1.16	24.88	0.67	99.05
28	3.30	6.10	2.96	0.62	59.61	0.93	26.33	0.72	100.6
29	4.53	8.48	1.17	0.70	57.07	0.88	26.36	n.d.	99.20
30	3.01	7.10	1.94	0.77	59.78	1.12	24.45	1.40	99.57
31	3.99	6.93	1.84	0.77	60.14	1.08	24.45	1.67	100.9
32	3.73	6.36	2.95	0.79	59.27	0.79	26.08	0.51	100.5
33	1.52	5.85	3.63	0.77	58.24	0.59	28.36	0.87	99.82
34	1.83	5.28	3.80	0.83	58.05	0.89	27.83	1.11	99.63
36	4.03	6.06	4.38	0.77	62.72	0.57	20.91	0.73	100.2
37	5.20	6.39	1.69	0.76	61.27	1.22	22.91	1.15	100.6
38	4.66	6.47	1.94	0.80	59.57	1.09	23.70	1.80	100.0
39	3.08	6.17	3.16	0.80	58.04	0.84	26.56	0.44	99.09
40	1.35	6.08	3.78	0.81	59.38	0.62	26.50	0.73	99.26
41	2.95	8.98	1.46	0.72	58.82	1.46	21.97	3.60	99.97
18	1.97	5.23	3.92	0.69	56.53	0.78	29.96	0.58	99.65
19	8.82	6.44	3.57	0.80	59.63	0.51	18.88	n.d.	98.64
20	3.98	3.14	1.39	0.70	62.71	0.89	26.11	0.88	99.80
22	2.88	6.43	3.24	0.81	59.36	0.74	25.55	1.09	100.1
26	3.39	5.61	3.78	0.73	57.95	0.64	27.61	n.d.	99.71
29	7.62	6.07	2.48	0.69	59.62	0.59	22.16	0.51	99.74
32	7.73	5.41	3.87	0.78	59.45	0.53	20.72	0.66	99.16
45	2.56	5.73	2.22	0.79	60.13	1.23	24.76	1.55	98.97
53	2.01	5.08	2.33	0.78	60.40	0.63	27.90	0.92	100.0
57	3.70	6.70	1.88	0.75	60.57	1.37	23.45	1.09	99.50
60	2.86	5.34	2.54	0.68	61.83	0.93	23.18	3.39	100.8
64	2.88	5.96	3.22	0.74	60.02	0.86	24.30	2.56	100.6
5	3.78	6.74	2.36	0.74	60.05	1.00	24.81	0.50	99.98
17	4.73	5.10	1.91	0.75	61.61	1.50	22.49	1.51	99.61
63	1.95	5.64	5.08	0.70	56.06	0.41	30.00	n.d.	99.84

OtC-V grains 32-63 μm

Grain	MgO	Al_2O_3	TiO_2	V_2O_3	Cr_2O_3	MnO	FeO	ZnO	Total
31	6.12	11.91	0.76	0.73	56.18	0.89	22.06	1.42	100.1
61	3.09	13.72	0.65	0.71	52.43	n.d.	28.28	n.d.	98.88
62	3.86	8.88	0.76	0.52	56.79	0.68	29.43	n.d.	100.9
65	7.71	19.64	0.63	0.45	49.09	0.51	21.34	n.d.	99.37

OtC grains 32-63 μm

Grain	MgO	Al_2O_3	TiO_2	V_2O_3	Cr_2O_3	MnO	FeO	ZnO	Total
1	5.29	13.86	0.93	0.40	53.53	0.97	23.34	n.d.	98.32
2	8.11	9.34	0.42	n.d.	56.78	n.d.	25.05	n.d.	99.69
3	13.10	31.02	0.75	n.d.	37.28	n.d.	18.95	n.d.	101.1
6	10.19	11.57	n.d.	n.d.	53.64	0.60	23.20	n.d.	99.21
7	6.10	11.16	0.99	0.35	57.14	0.75	22.44	0.59	99.52
8	13.52	26.71	0.74	n.d.	41.45	0.52	17.67	n.d.	100.6
9	6.06	10.04	1.82	0.44	58.03	1.31	19.48	3.54	100.7
10	9.12	0.34	n.d.	n.d.	50.71	2.82	33.62	3.20	99.82
11	7.44	24.59	0.93	n.d.	37.42	0.58	28.16	0.40	99.51
12	6.84	7.48	n.d.	n.d.	59.65	n.d.	27.63	n.d.	101.6
14	13.71	28.01	0.45	n.d.	34.99	0.31	22.85	n.d.	100.3
15	14.34	37.19	1.29	n.d.	21.61	n.d.	24.72	n.d.	99.14
21	10.72	36.20	0.49	n.d.	33.09	0.43	18.59	n.d.	99.51
24	6.06	15.92	0.46	0.31	46.25	0.59	29.61	0.32	99.53
25	13.27	27.57	n.d.	n.d.	44.36	0.42	14.73	0.24	100.6
27	9.50	24.77	0.98	0.31	33.45	0.53	29.75	0.27	99.56
28	7.40	6.30	n.d.	n.d.	59.36	0.59	25.88	n.d.	99.53
30	8.12	30.89	0.28	n.d.	36.42	n.d.	23.75	n.d.	99.46
33	3.93	3.47	8.57	n.d.	5.25	0.64	77.72	n.d.	99.58
34	10.59	12.01	n.d.	n.d.	53.98	n.d.	22.42	n.d.	99.00
35	11.67	30.69	0.65	0.38	19.89	n.d.	36.47	n.d.	99.76
36	0.94	0.42	0.30	n.d.	50.02	1.80	34.86	10.86	99.20
37[§]	2.74	3.93	n.d.	0.33	60.24	0.96	29.79	0.48	99.31
38	8.93	13.99	2.03	n.d.	49.39	n.d.	25.02	n.d.	99.36
39	2.46	4.73	1.73	0.32	51.07	0.79	38.09	0.45	99.65
40	5.10	7.73	0.74	0.30	61.00	1.63	17.80	5.43	99.73
41	13.70	37.02	0.52	n.d.	24.29	0.29	22.94	n.d.	98.76
42	1.48	27.46	n.d.	n.d.	33.10	1.22	29.49	7.11	99.86
43	13.55	28.64	1.08	n.d.	33.50	n.d.	22.01	n.d.	98.78
44	6.57	28.25	0.33	n.d.	34.03	0.49	31.23	0.62	101.5
46	9.82	33.81	0.32	0.24	29.38	0.44	25.35	n.d.	99.35
47	10.13	13.18	n.d.	n.d.	55.90	n.d.	17.20	2.94	99.35
48	4.41	19.44	n.d.	n.d.	47.32	0.71	26.76	1.24	99.88
49	6.93	14.71	1.56	n.d.	45.41	0.44	30.51	n.d.	99.56
50	13.88	20.28	0.59	n.d.	51.15	n.d.	15.49	n.d.	101.4
51	n.d.	2.9	n.d.	n.d.	51.24	1.39	35.54	9.32	99.39
52	12.38	23.71	1.09	n.d.	44.59	n.d.	17.41	n.d.	99.19
54	9.25	19.03	0.91	0.39	31.53	0.47	37.40	n.d.	98.99
55	0.30	15.91	0.66	0.25	39.76	1.18	38.35	3.70	100.1
56	13.93	13.14	n.d.	n.d.	57.27	n.d.	15.09	n.d.	99.42
58	17.00	33.79	0.32	n.d.	34.68	n.d.	13.65	n.d.	99.44
59	11.45	27.82	1.47	0.33	18.53	n.d.	39.84	n.d.	99.44
66	11.04	22.09	0.68	0.25	44.54	n.d.	21.68	n.d.	100.3
67	6.45	13.42	n.d.	n.d.	52.76	0.46	26.85	n.d.	99.94
68	5.54	26.70	n.d.	n.d.	32.74	n.d.	33.40	0.86	99.27
69	6.33	0.46	n.d.	n.d.	49.18	1.94	39.92	1.21	99.03
70	13.10	24.44	n.d.	0.32	46.22	0.45	14.39	0.47	99.39
13	8.94	18.67	1.07	0.54	30.17	n.d.	40.20	n.d.	99.58
16	8.89	17.41	3.23	0.66	21.37	0.41	48.46	n.d.	100.4

[§]Also NiO 0.85 wt%.

-3.12 to -3.27 m

Sample weight 28.1 kg. Results for >63 µm fraction from Schmitz and Häggström (2006), but 32-63 µm fraction studied here.

EC grains >63 µm

Grain	MgO	Al_2O_3	TiO_2	V_2O_3	Cr_2O_3	MnO	FeO	ZnO	Total
1	4.31	6.34	2.03	0.66	60.47	1.39	23.78	0.75	99.74

OtC-V grains >63 µm

Grain	MgO	Al_2O_3	TiO_2	V_2O_3	Cr_2O_3	MnO	FeO	ZnO	Total
1	6.09	11.34	0.95	0.93	56.88	0.95	22.45	0.28	99.86

OtC grains >63 µm

Grain	MgO	Al_2O_3	TiO_2	V_2O_3	Cr_2O_3	MnO	FeO	ZnO	Total
1	6.87	8.07	n.d.	0.19	57.38	0.67	25.54	n.d.	98.91

EC grains 32-63 µm

Grain	MgO	Al_2O_3	TiO_2	V_2O_3	Cr_2O_3	MnO	FeO	ZnO	Total
1	3.64	7.63	2.04	0.83	61.18	1.41	21.59	1.70	100.0
2	2.38	6.14	3.27	0.82	59.90	0.71	27.89	0.28	101.4
3	4.48	6.90	2.24	0.88	60.63	1.09	24.94	n.d.	101.2
4	2.20	5.78	2.98	0.83	58.10	1.04	29.26	n.d.	100.2
5	1.31	6.23	3.37	0.81	61.54	0.71	25.05	0.50	99.52
6	2.43	5.86	3.38	0.82	61.23	1.14	22.84	2.57	100.3
7	2.93	6.49	3.90	0.81	61.71	0.71	23.88	n.d.	100.4
8	2.27	6.95	3.65	0.77	65.48	0.69	20.69	0.74	101.2
9	2.71	5.98	3.79	0.71	57.29	0.87	27.63	1.08	100.1
10	1.92	6.22	3.71	0.73	59.08	0.80	26.89	0.86	100.2
11	3.21	7.05	2.43	0.73	64.19	0.89	20.63	1.20	100.3
12	3.93	6.19	4.62	0.76	61.41	0.62	23.18	n.d.	100.7
13	3.34	7.06	1.36	0.86	62.14	1.47	22.21	1.95	100.4
14	2.73	5.12	1.45	0.79	65.52	2.00	18.93	4.64	101.2
15	3.30	6.34	2.40	0.77	63.22	1.38	20.78	2.48	100.7
16	3.52	6.97	1.66	0.78	62.89	1.71	25.30	1.29	104.1

OtC-V grains 32-63 µm

Grain	MgO	Al_2O_3	TiO_2	V_2O_3	Cr_2O_3	MnO	FeO	ZnO	Total
1	0.38	10.43	1.77	0.72	53.96	0.46	31.14	1.09	99.95
2	3.98	8.55	0.75	0.61	61.68	1.46	24.05	0.41	101.5

OtC grains 32-63 µm

Grain	MgO	Al_2O_3	TiO_2	V_2O_3	Cr_2O_3	MnO	FeO	ZnO	Total
1	7.37	22.42	0.25	n.d.	46.99	n.d.	23.01	n.d.	100.1
2	9.46	32.94	0.34	n.d.	28.11	n.d.	29.53	0.67	101.0
3	14.46	39.12	1.28	n.d.	19.83	n.d.	23.94	n.d.	98.64
4	12.88	20.71	1.52	n.d.	42.82	n.d.	22.47	n.d.	100.4
5	0.29	17.04	0.55	0.39	39.53	1.67	36.34	5.04	100.9
6	15.24	26.57	0.53	0.25	38.99	n.d.	17.76	n.d.	99.34
7	14.04	39.83	n.d.	n.d.	24.13	n.d.	23.23	n.d.	101.2

8	7.34	13.14	0.78	n.d.	46.32	n.d.	32.46	n.d.	100.0
9	2.67	6.83	n.d.	n.d.	50.68	0.72	38.95	0.59	100.4
10	3.16	11.90	1.39	n.d.	53.99	0.44	28.67	0.50	100.0
11	7.83	27.73	0.41	n.d.	37.10	n.d.	26.95	0.37	100.4
12	9.33	15.67	1.85	0.41	44.93	n.d.	27.02	n.d.	99.21
13	2.22	12.25	n.d.	n.d.	52.01	2.79	30.49	1.48	101.3
14	7.35	6.51	n.d.	n.d.	62.35	1.05	23.20	n.d.	100.5
15	13.59	28.48	0.86	n.d.	38.62	n.d.	18.99	n.d.	100.5
16	9.59	18.73	n.d.	0.40	55.69	n.d.	14.70	0.81	99.92
17	8.53	13.91	1.44	n.d.	34.60	n.d.	41.26	n.d.	99.74
18	6.10	17.03	0.53	n.d.	47.00	0.53	29.33	0.44	101.0
19	2.58	3.21	n.d.	0.25	63.51	0.76	29.31	0.76	100.4
20	9.83	13.86	0.50	n.d.	54.05	n.d.	21.22	n.d.	99.45
21	0.30	16.32	1.77	n.d.	38.42	0.62	42.59	0.49	100.5
22	5.60	10.63	1.82	n.d.	28.37	0.59	53.32	n.d.	100.3
23	1.63	3.18	2.02	0.26	41.57	0.63	51.06	0.58	100.9
24	7.07	29.91	0.58	n.d.	25.70	0.42	35.47	0.42	99.57
25	11.94	35.21	0.53	n.d.	21.16	n.d.	31.42	n.d.	100.3
26	0.43	14.86	n.d.	n.d.	43.92	2.34	34.27	4.26	100.1
27	13.01	28.73	1.26	n.d.	36.74	n.d.	19.68	n.d.	99.42
28[§]	9.32	29.94	n.d.	n.d.	25.28	0.46	33.30	1.14	99.89
29	9.32	35.99	n.d.	n.d.	32.97	n.d.	21.51	0.34	100.1
30	0.96	2.17	1.25	0.95	49.15	1.37	35.46	6.77	98.07
31	1.74	8.66	1.14	1.12	49.33	0.50	36.27	1.67	100.4

[§]Also NiO 0.45 wt%.

-3.27 to 3.45 m

Sample weight 27.7 kg. Results for >63 µm fraction from Schmitz and Häggström (2006), but 32-63 µm fraction studied here.

EC grains >63 µm

Grain	MgO	Al_2O_3	TiO_2	V_2O_3	Cr_2O_3	MnO	FeO	ZnO	Total
1	4.76	5.84	2.39	0.70	60.21	1.37	23.94	0.79	100.0

EC grains 32-63 µm

Grain	MgO	Al_2O_3	TiO_2	V_2O_3	Cr_2O_3	MnO	FeO	ZnO	Total
1	2.66	6.17	3.99	0.77	57.32	0.86	28.68	0.68	101.1
2	2.59	5.72	4.07	0.73	56.39	0.79	29.07	0.50	99.85
3	4.73	6.90	1.87	0.77	60.26	1.41	23.26	2.31	101.5
4	2.27	6.43	2.79	0.82	61.31	0.92	25.71	n.d.	100.3
5	2.11	6.05	3.71	0.74	59.89	n.d.	27.75	0.43	100.7
6	5.78	6.03	4.23	0.77	57.51	n.d.	24.69	n.d.	99.01
7	3.06	7.63	1.91	0.66	59.96	1.29	25.77	0.52	100.8
8	4.38	6.41	2.01	0.76	60.29	1.28	23.78	0.93	99.85
9	2.63	6.27	2.96	0.80	58.26	0.79	29.09	0.59	101.4
10	1.98	5.49	3.69	0.72	61.26	0.77	25.78	0.45	100.1
11	4.41	5.02	1.16	0.88	63.31	0.58	23.61	0.69	99.66
12	4.69	5.76	3.76	0.74	57.99	0.70	25.81	0.44	99.88
13	2.93	6.82	2.10	0.80	61.58	1.40	23.91	0.60	100.1

OtC-V grains 32-63 µm

Grain	MgO	Al_2O_3	TiO_2	V_2O_3	Cr_2O_3	MnO	FeO	ZnO	Total
1	1.29	10.93	2.37	0.68	60.86	0.84	23.29	n.d.	100.3
2	2.26	12.81	3.60	0.82	38.96	0.52	25.73	15.70	100.4
3	0.20	8.36	7.75	0.61	49.84	0.74	32.39	n.d.	99.89

OtC grains 32-63 µm

Grain	MgO	Al_2O_3	TiO_2	V_2O_3	Cr_2O_3	MnO	FeO	ZnO	Total
1	6.67	25.50	1.36	0.36	28.02	0.53	36.14	0.37	98.95
2	7.24	16.75	0.33	n.d.	51.18	n.d.	23.17	n.d.	98.66
3	9.59	15.82	0.30	n.d.	49.91	0.56	23.98	n.d.	100.2
4§	0.62	3.79	2.26	0.30	42.28	0.91	32.88	12.36	99.36
5	13.72	18.21	0.32	n.d.	46.32	0.55	21.51	n.d.	100.6
6	8.23	12.17	n.d.	n.d.	55.21	n.d.	24.04	n.d.	99.64
7	6.59	30.55	n.d.	0.39	27.73	n.d.	34.07	0.86	100.2
8§	0.76	15.50	1.90	0.38	34.61	0.92	35.64	6.40	98.30
9	10.40	28.01	2.96	n.d.	24.03	n.d.	33.28	n.d.	98.68
10	13.32	38.34	1.28	0.22	21.94	n.d.	23.81	n.d.	98.92
11	11.45	31.85	0.58	n.d.	27.89	n.d.	27.87	n.d.	99.64
12	5.30	19.84	2.65	0.40	21.12	2.84	46.34	0.46	98.95
13	11.49	26.20	0.30	n.d.	37.88	0.39	24.55	n.d.	100.8
14	2.80	10.32	0.86	n.d.	47.48	0.87	37.68	0.52	100.5
15	8.19	14.64	0.42	n.d.	49.13	0.49	27.09	n.d.	99.96
16	8.11	21.47	1.03	0.25	37.87	n.d.	31.04	n.d.	99.78
17	9.19	24.34	0.84	0.36	32.89	n.d.	32.75	n.d.	100.4
18'	14.30	37.62	0.56	n.d.	28.59	n.d.	19.30	n.d.	100.8
19	3.00	6.27	7.33	0.60	24.20	0.67	54.23	0.44	96.74

§Also some Si, P, Ca, ' NiO 0.38 wt%.

-4.30 to -4.50 m

Sample weight 28.1 kg. Results for >63 µm fraction from Schmitz and Häggström (2006), but 32-63 µm fraction studied here.

EC grains 32-63 µm

Grain	MgO	Al_2O_3	TiO_2	V_2O_3	Cr_2O_3	MnO	FeO	ZnO	Total
1	2.09	5.56	2.72	0.66	59.87	0.72	27.88	0.33	99.83
2	1.82	6.13	2.70	0.77	59.33	0.93	29.53	0.45	101.7
3	7.22	6.26	1.32	0.69	63.03	0.84	20.54	0.43	100.3
4	2.45	6.74	1.94	0.71	57.90	1.26	26.11	1.33	98.43
5	2.91	6.06	3.69	0.68	58.14	0.73	28.77	n.d.	101.0
6	2.80	6.20	2.40	0.72	58.54	1.03	28.69	n.d	100.4
7	1.81	5.42	4.04	0.77	57.47	0.82	29.94	0.53	100.8
8	2.72	6.21	2.60	0.80	60.36	1.09	28.02	0.64	102.4

OtC-V grains 32-63 µm

Grain	MgO	Al_2O_3	TiO_2	V_2O_3	Cr_2O_3	MnO	FeO	ZnO	Total
1	2.74	10.36	2.96	0.55	54.35	0.66	30.22	n.d	101.9

OtC grains 32-63 µm

Grain	MgO	Al_2O_3	TiO_2	V_2O_3	Cr_2O_3	MnO	FeO	ZnO	Total
1	6.10	9.94	n.d.	0.32	67.38	n.d.	15.23	n.d.	98.97
2	13.55	31.26	0.75	0.24	26.58	n.d.	27.90	n.d.	100.3
3	6.84	28.88	0.40	n.d.	25.89	0.55	36.01	0.89	99.46
4	11.07	27.83	0.51	n.d.	32.44	n.d.	29.51	n.d.	101.4
5	9.86	14.66	n.d.	0.24	52.10	n.d.	23.78	n.d.	100.6
6	14.43	33.04	0.76	n.d.	26.36	n.d.	25.55	n.d.	100.1
7	12.69	28.47	0.80	n.d.	28.69	n.d.	27.83	n.d.	98.50
8	13.90	32.11	0.59	0.21	26.30	n.d.	26.09	n.d.	99.22
9	11.29	25.17	1.18	0.35	28.64	n.d.	33.26	n.d	99.89
10	11.95	26.10	0.92	0.32	29.04	n.d.	31.57	n.d.	99.90
11	4.95	23.05	0.67	n.d.	38.29	n.d.	32.51	n.d.	99.46
12	2.15	13.76	0.85	n.d.	32.15	n.d.	49.60	0.75	99.25
13	7.44	37.93	n.d.	n.d.	22.02	n.d.	30.80	0.84	99.03
14	10.93	16.15	n.d.	0.26	53.32	n.d.	18.94	n.d.	99.60

-4.50 to -4.58 m

Sample weight 20.6 kg. Results for >63 µm fraction from Schmitz and Häggström (2006), but 32-63 µm fraction studied here.

EC grains >63 µm

Grain	MgO	Al_2O_3	TiO_2	V_2O_3	Cr_2O_3	MnO	FeO	ZnO	Total
1	3.44	6.03	1.43	0.78	61.59	1.06	23.37	0.59	98.29
2	3.21	6.35	2.40	0.71	59.68	1.14	23.46	0.34	97.29

EC grains 32-63 µm

Grain	MgO	Al_2O_3	TiO_2	V_2O_3	Cr_2O_3	MnO	FeO	ZnO	Total
1	2.03	6.48	3.37	0.92	62.14	1.32	22.32	1.87	100.4
2	1.95	5.98	3.28	0.69	60.33	0.89	26.20	0.47	99.79
3	4.57	6.82	1.78	0.74	60.57	1.70	23.32	1.32	100.8
4	3.48	6.49	2.34	0.75	60.63	0.88	23.98	0.17	98.73
5	3.00	6.31	2.83	0.85	57.16	0.78	28.21	0.53	99.66
6	3.87	6.48	2.53	0.64	60.44	1.05	26.26	0.60	101.9
7	2.34	6.34	3.10	0.79	60.51	0.94	26.20	0.68	100.9

OtC grains 32-63 µm

Grain	MgO	Al_2O_3	TiO_2	V_2O_3	Cr_2O_3	MnO	FeO	ZnO	Total
1	15.03	38.29	n.d.	n.d.	30.60	n.d.	16.70	n.d.	100.6
2	6.18	10.87	n.d.	n.d.	59.35	0.58	22.26	0.22	99.47
3	11.48	31.36	n.d.	n.d.	36.13	n.d.	22.09	n.d.	101.1
4	9.45	22.38	0.40	n.d.	45.21	n.d.	21.10	n.d.	98.54
5	9.32	13.17	1.06	n.d.	57.95	n.d.	16.11	2.65	100.3
6	11.56	24.95	1.19	0.29	37.71	n.d.	25.57	n.d.	101.3
7	15.13	31.57	0.78	n.d.	33.99	n.d.	18.59	n.d.	100.1
8	11.33	26.84	0.40	n.d.	40.87	n.d.	20.10	n.d.	99.54
9	12.46	23.59	0.72	n.d.	42.48	n.d.	20.65	n.d.	99.89
10	6.70	21.89	0.37	n.d.	37.39	0.41	31.68	n.d.	98.46

11	1.92	5.83	4.80	0.51	23.49	0.47	62.83	n.d.	99.84

-5.00 to -5.25 m

Sample weight 30.5 kg. From Schmitz and Häggström (2006). Only searched for grains in >63 µm fraction, no chrome spinel grains found.

-5.45 to -5.70 m

Sample weight 28.0 kg. From Schmitz and Häggström (2006). Only searched for grains in >63 µm fraction, no chrome-spinel grains found.

-5.88 to -6.08 m

Sample weight 23.1 kg. Results for >63 µm fraction from Schmitz and Häggström (2006), but 32-63 µm fraction studied here.

OtC-V grains >63 µm

Grain	MgO	Al_2O_3	TiO_2	V_2O_3	Cr_2O_3	MnO	FeO	ZnO	Total
1	4.52	9.06	0.45	0.80	62.10	1.42	20.60	0.72	99.67

OtC grains >63 µm

Grain	MgO	Al_2O_3	TiO_2	V_2O_3	Cr_2O_3	MnO	FeO	ZnO	Total
1	12.15	33.71	1.03	0.23	30.12	0.49	20.81	0.63	99.17

EC grains 32-63 µm

Grain	MgO	Al_2O_3	TiO_2	V_2O_3	Cr_2O_3	MnO	FeO	ZnO	Total
1	7.36	5.90	3.62	0.70	59.40	0.58	22.86	n.d.	100.4
2	6.47	5.64	3.56	0.61	60.41	0.69	23.78	n.d.	101.2
3	1.39	6.49	3.83	0.69	59.17	n.d.	28.62	n.d.	100.2

OtC-V grains 32-63µm

Grain	MgO	Al_2O_3	TiO_2	V_2O_3	Cr_2O_3	MnO	FeO	ZnO	Total
1	5.28	9.21	0.45	0.81	61.68	1.61	19.41	1.19	99.65
2	5.24	9.40	0.51	0.69	60.51	1.71	19.44	2.28	99.78
3	5.33	12.21	0.32	0.48	53.31	n.d.	27.26	n.d.	98.92

OtC grains 32-63 µm

Grain	MgO	Al_2O_3	TiO_2	V_2O_3	Cr_2O_3	MnO	FeO	ZnO	Total
1	13.65	32.83	1.34	n.d.	32.73	n.d.	20.10	n.d.	100.7
2	14.20	23.59	0.48	n.d.	44.60	n.d.	17.32	n.d.	100.2
3	16.89	35.30	0.88	n.d.	30.33	n.d.	16.46	n.d.	99.86

4	14.75	39.12	1.39	n.d.	21.79	0.45	21.73	n.d.	99.23
5	9.68	19.55	0.75	n.d.	48.72	0.51	20.19	n.d.	99.41
6	11.20	23.35	0.69	n.d.	43.36	n.d.	20.91	n.d.	99.51
7	16.08	39.85	n.d.	n.d.	29.89	n.d.	13.82	n.d.	99.65
8	13.92	24.43	0.47	n.d.	44.56	n.d.	17.36	n.d.	100.7
9	11.69	32.43	0.85	n.d.	32.99	n.d.	21.25	n.d.	99.21
10	12.41	25.25	0.86	n.d.	42.38	n.d.	19.36	n.d.	100.3
11	10.63	20.17	0.77	n.d.	49.02	n.d.	18.43	n.d.	99.01
12	9.13	21.81	2.33	0.38	37.43	n.d.	27.80	n.d.	98.87
13	5.58	18.07	2.06	0.44	36.91	0.56	37.22	n.d.	100.8
14	12.55	22.95	0.59	n.d.	46.22	n.d.	18.25	n.d.	100.6
15	4.34	18.48	0.75	0.41	39.15	0.30	35.74	n.d.	99.17
16	11.48	22.55	0.57	n.d.	46.74	n.d.	17.69	n.d.	99.03
17	13.69	27.05	1.20	n.d.	40.49	n.d.	17.22	n.d.	99.65
18	13.80	32.28	0.91	n.d.	33.66	n.d.	18.71	n.d.	99.37
19	14.94	35.58	0.68	n.d.	32.63	n.d.	16.57	n.d.	100.4
20	15.07	31.55	0.75	n.d.	36.27	n.d.	16.44	n.d.	100.1
21	13.76	33.54	1.55	n.d.	31.15	n.d.	20.05	n.d.	100.1
22	10.66	26.26	0.34	n.d.	43.52	0.36	18.47	n.d.	99.61
23	11.57	22.98	n.d.	n.d.	45.66	n.d.	20.04	0.23	100.5
24	6.98	6.10	0.38	n.d.	57.56	n.d.	28.66	n.d.	99.68
25	12.14	31.11	0.73	n.d.	38.88	n.d.	18.1	n.d.	101.0
26	10.07	15.14	0.69	n.d.	47.46	n.d.	26.62	n.d.	99.99
27	11.39	15.25	0.67	n.d.	46.24	n.d.	25.18	n.d.	98.72
28	14.84	27.14	0.33	n.d.	41.23	n.d.	16.25	n.d.	99.79
29	9.43	11.03	n.d.	n.d.	57.97	0.65	20.98	n.d.	100.1
30	2.18	1.65	4.12	0.99	20.86	0.43	60.96	n.d.	91.20
31	12.31	31.93	0.87	0.57	30.58	n.d.	23.60	n.d.	99.86

-6.08 to -6.18 m

Sample weight 20.0 kg. Only the 32-63 µm fraction studied here. Schmitz and Häggström (2006) found no chrome spinel grains in the >63 µm fraction.

EC grains 32-63 µm

Grain	MgO	Al_2O_3	TiO_2	V_2O_3	Cr_2O_3	MnO	FeO	ZnO	Total
1	2.72	5.74	4.32	0.72	57.91	0.83	28.83	n.d.	101.1
2	2.93	6.12	2.97	0.84	57.92	1.00	27.02	0.75	99.55

OtC-V grains 32-63 µm

Grain	MgO	Al_2O_3	TiO_2	V_2O_3	Cr_2O_3	MnO	FeO	ZnO	Total
1	0.51	13.95	3.31	0.56	51.58	0.72	29.81	n.d.	100.4
2	4.50	10.69	2.88	0.56	54.26	n.d.	27.11	n.d.	100.0
3	4.53	11.29	2.19	0.46	56.27	0.53	24.14	n.d.	99.41

OtC grains 32-63 µm

Grain	MgO	Al_2O_3	TiO_2	V_2O_3	Cr_2O_3	MnO	FeO	ZnO	Total
1	15.93	36.33	0.91	n.d.	28.36	n.d.	18.81	n.d.	100.3
2	10.40	24.78	1.77	0.26	22.69	n.d.	40.38	n.d.	100.3
3	10.64	23.19	1.97	0.31	39.93	n.d.	23.44	0.19	99.68
4	12.96	25.30	0.96	n.d.	43.06	n.d.	17.33	n.d.	99.61

5	12.84	23.40	0.50	0.23	45.96	n.d.	17.31	n.d.	100.2
6	7.75	6.95	1.12	n.d.	56.74	n.d.	27.52	n.d.	100.1
7	4.23	17.44	n.d.	n.d.	40.54	0.58	35.78	0.48	99.04
8	10.67	8.26	0.25	0.29	42.68	n.d.	37.76	n.d.	99.91
9	11.66	30.75	0.80	0.25	24.05	n.d.	33.20	n.d.	100.7
10	12.54	28.68	1.62	n.d.	35.86	0.27	21.38	n.d.	100.4
11	9.92	9.02	n.d.	n.d.	65.53	0.40	14.61	0.32	99.80
12	4.82	19.19	0.49	0.43	49.53	0.53	24.32	n.d.	99.31
13	15.63	34.75	0.32	n.d.	34.20	n.d.	14.69	n.d.	99.59
14	13.89	26.90	1.66	0.29	33.28	n.d.	23.53	n.d.	99.55
15	14.39	31.05	0.80	n.d.	35.15	n.d.	17.87	n.d.	99.25
16	7.96	9.14	n.d.	0.44	57.12	n.d.	25.01	n.d.	99.67
17	7.93	18.02	3.54	0.70	31.96	0.48	37.23	n.d.	99.85
18	8.72	15.54	8.84	0.81	22.72	n.d.	41.23	n.d.	97.86

-9.05 to -9.15 m

Weight 26.4 kg. From Schmitz and Häggström (2006). Only searched for grains in >63 µm fraction, no chrome spinel grains found
