# Supplementary material for: An extraterrestrial trigger for the mid-Ordovician ice age: Dust from the breakup of the L-chondrite parent body
Source: Sci Adv. 2019 Sep 18;5(9):eaax4184. doi: 10.1126/sciadv.aax4184 (PMC6750910; doi:10.1126/sciadv.aax4184)
Supplement: Data file S5 [file aax4184_Data_file_S5.pdf]

DATA S5 - Lynna River section lower part - chrome-spinel chemical results

Element composition (wt%) of chrome spinel grains.

Samples in the "GAP" series, i.e. GG1, 2, 3, 3.5, 4, 6 and 7 were all prepared for this study. Samples 31, 30, 28u were prepared by Lindskog et al. (2011) (*76*), who only studied the >63 µm fractions. For this study we searched the 32-63 µm residues of the same samples for chrome spinels.

All analyses on polished grains, except where otherwise indicated. n.d. = not detected.

When grains are accepted as EC grains although single element concentrations lie outside the accepted EC range, the outlier values are marked in red, see further data S1.

Sample depths are given relative to the base of bed Ly1 in Lindskog et al. (*76*).

Sample G1

0.20-0.40 m below base of Ly1, sample weight: 14.2 kg.

EC grains 32-63 µm

Grain	MgO	Al_2O_3	TiO_2	V_2O_3	Cr_2O_3	MnO	FeO	ZnO	Total
1	2.95	6.26	2.76	0.81	58.90	1.06	26.10	1.23	100.1
2	2.52	5.82	3.22	0.69	58.62	0.89	28.43	n.d.	100.2
3	2.79	6.08	3.24	0.75	58.57	1.03	27.45	0.76	100.7
4	3.35	6.03	3.21	0.73	58.44	0.84	26.77	0.47	99.85
5	2.85	6.14	3.34	0.71	59.97	0.88	26.32	n.d.	100.2
6	2.58	6.69	3.23	0.68	59.61	0.84	26.74	0.50	100.9
7	3.05	6.17	3.29	0.73	59.54	0.99	27.07	n.d.	100.8
8	2.24	6.70	2.88	0.74	59.13	0.91	25.09	1.81	99.50
9	2.61	6.17	3.26	0.69	58.16	0.91	28.65	n.d.	100.5
10	1.98	6.69	2.76	0.76	58.92	0.78	25.47	2.45	99.80
11	2.43	6.26	3.02	0.73	59.58	0.81	27.21	0.47	100.5
12	2.57	5.84	2.74	0.74	59.15	1.10	28.19	0.52	100.8
13	2.74	6.19	2.74	0.78	59.73	1.12	22.39	3.61	99.30
14	2.53	6.94	2.82	0.84	58.93	0.97	22.81	4.16	100.0
15	3.00	5.75	3.18	0.73	58.60	0.91	27.56	n.d.	99.73
16	1.90	5.76	3.90	0.70	57.33	0.93	27.21	1.08	98.80
17	3.36	4.73	2.69	0.62	61.77	0.57	26.11	n.d.	99.85
18	2.85	6.60	2.88	0.77	59.15	1.05	26.03	0.83	100.1
19	3.37	5.91	3.30	0.71	59.11	1.02	26.62	0.61	100.7
20	2.70	6.23	3.25	0.69	58.93	0.95	25.06	3.01	100.8
21	2.49	6.86	2.35	0.78	59.62	0.97	22.87	3.30	99.25
22	2.37	6.29	2.21	0.77	60.31	1.15	21.99	5.15	100.2
23	3.06	5.97	3.17	0.70	58.66	1.06	27.44	n.d.	100.1
24	3.34	6.10	3.10	0.71	58.60	0.83	27.45	0.39	100.5
25	2.81	6.38	2.91	0.75	60.32	1.04	21.95	4.50	100.7
26	2.36	6.04	2.76	0.81	59.69	0.79	28.09	0.42	101.0

27	2.83	6.54	2.93	0.78	59.65	0.88	26.64	n.d.	100.3
28	3.00	6.02	3.05	0.73	58.62	1.02	27.65	n.d.	100.1
29	2.89	6.26	3.03	0.70	58.21	0.85	27.23	0.69	99.86
30	2.87	6.06	3.03	0.79	58.40	1.12	22.02	5.14	99.43

EC grains >63 μm

Grain	MgO	Al_2O_3	TiO_2	V_2O_3	Cr_2O_3	MnO	FeO	ZnO	Total
1	2.74	6.06	3.09	0.69	58.79	0.82	27.13	0.48	99.79

OtC-V grains 32-63 μm

Grain	MgO	Al_2O_3	TiO_2	V_2O_3	Cr_2O_3	MnO	FeO	ZnO	Total
1	5.29	16.06	1.11	0.78	49.22	n.d.	27.00	n.d.	99.45

OtC grains 32-63 μm

Grain	MgO	Al_2O_3	TiO_2	V_2O_3	Cr_2O_3	MnO	FeO	ZnO	Total
1	6.11	6.23	n.d.	n.d.	55.74	n.d.	30.73	n.d.	99.21
2	14.31	33.79	n.d.	n.d.	30.17	n.d.	21.6	n.d.	99.87

Sample G2

0.70-0.90 m below base of Ly1, sample weight: 11.7 kg.

EC grains 32-63 μm

Grain	MgO	Al_2O_3	TiO_2	V_2O_3	Cr_2O_3	MnO	FeO	ZnO	Total
1*	1.40	5.93	2.73	0.78	54.19	n.d.	27.45	n.d.	92.48

*analysis on unpolished grain

OtC grains 32-63 μm

Grain	MgO	Al_2O_3	TiO_2	V_2O_3	Cr_2O_3	MnO	FeO	ZnO	Total
1	14.02	9.92	n.d.	n.d.	58.44	n.d	17.43	n.d.	99.80
2*	10.58	17.37	0.66	n.d.	47.04	n.d.	19.73	n.d.	95.67

*analysis on unpolished grain

Sample G3

1.10-1.30 m below base of Ly1, sample weight: 12.3 kg.

EC grains 32-63 μm

Grain*	MgO	Al_2O_3	TiO_2	V_2O_3	Cr_2O_3	MnO	FeO	ZnO	Total
1	1.43	6.14	3.11	0.82	60.26	1.00	21.91	5.34	100.0

2	3.35	6.74	2.44	0.69	58.71	1.20	25.11	1.56	99.81

* one grain with similar composition as the two others was lost during polishing

OtC grain 32-63 µm

Grain	MgO	Al_2O_3	TiO_2	V_2O_3	Cr_2O_3	MnO	FeO	ZnO	Total
1*	9.64	19.41	0.68	n.d.	27.42	n.d	25.53	n.d.	82.68

*analysis on unpolished grain

Samples G3.5 and G4

G3.5, 1.90-2.06 m below base of Ly1, sample weight: 8.8 kg. No chrome spinel grains in 32-63 µm and >63 µm fractions.

G4, 2.06-2.13 m below base of Ly1, sample weight: 10.9 kg. No chrome spinel grains in 32-63 µm and >63 µm fractions.

Sample 31

2.10-2.30 m below base of Ly1, sample weight: 9.8 kg, see Lindskog et al. (*76*).

EC grain 32-63 µm

Grain	MgO	Al_2O_3	TiO_2	V_2O_3	Cr_2O_3	MnO	FeO	ZnO	Total
1	3.19	7.61	1.76	0.77	58.81	1.32	26.43	0.37	100.3

OtC-V grain 32-63 µm

Grain	MgO	Al_2O_3	TiO_2	V_2O_3	Cr_2O_3	MnO	FeO	ZnO	Total
1	2.50	9.20	0.51	0.67	58.49	0.59	27.91	0.35	100.2

OtC grains 32-63 µm

Grain	MgO	Al_2O_3	TiO_2	V_2O_3	Cr_2O_3	MnO	FeO	ZnO	Total
1	12.63	24.04	0.73	n.d.	30.36	n.d.	32.66	n.d.	100.4
2	12.83	6.30	n.d.	n.d.	62.43	n.d.	18.08	n.d.	99.64

Sample 30

2.30 - 2.50 m below base of Ly1, sample weight: 14.4 kg, see Lindskog et al. (*76*).

EC grains 32-63 µm

Grain	MgO	Al_2O_3	TiO_2	V_2O_3	Cr_2O_3	MnO	FeO	ZnO	Total

1	3.12	6.37	2.41	0.77	60.04	1.04	26.25	0.37	100.4
2	2.87	6.53	2.34	0.69	60.14	0.77	26.43	0.65	100.4
3	2.32	6.03	2.38	0.78	59.38	0.88	28.51	0.32	100.6
4	3.45	7.12	1.53	0.77	60.44	1.07	23.71	2.46	100.5
5	4.19	6.69	2.19	0.73	58.40	0.99	25.91	n.d.	99.09
6	3.87	6.63	3.68	0.67	55.60	n.d.	28.67	0.37	99.49

EC grains >63 µm

Grain	MgO	Al_2O_3	TiO_2	V_2O_3	Cr_2O_3	MnO	FeO	ZnO	Total
1	2.91	3.49	2.55	0.66	60.87	0.69	28.22	n.d.	99.40

OtC-V grains 32-63 µm

Grain	MgO	Al_2O_3	TiO_2	V_2O_3	Cr_2O_3	MnO	FeO	ZnO	Total
1	5.53	16.01	0.97	0.58	48.30	n.d.	28.57	n.d.	99.96
2	3.58	12.91	0.48	0.71	54.27	0.77	27.14	n.d.	99.86

OtC grains 32-63 µm

Grain	MgO	Al_2O_3	TiO_2	V_2O_3	Cr_2O_3	MnO	FeO	ZnO	Total
1	6.96	18.68	1.09	n.d.	50.13	0.73	22.07	n.d.	99.67
2	13.26	21.63	0.72	n.d.	40.44	n.d.	23.62	n.d.	99.67

Sample 28u

2.80 - 2.95 m below base of Ly1, sample weight: 14.0 kg, see Lindskog et al. (*76*).

EC grains 32-63 µm

Grain	MgO	Al_2O_3	TiO_2	V_2O_3	Cr_2O_3	MnO	FeO	ZnO	Total
1	3.47	5.57	1.94	0.83	61.04	1.00	25.30	1.15	100.3
2	3.31	6.51	2.27	0.68	59.04	0.99	27.74	n.d.	100.6
3	4.13	7.02	2.26	0.74	58.54	1.06	25.55	n.d.	99.31
4	4.59	7.34	1.65	0.67	58.32	1.01	26.07	0.28	99.93
5	2.75	6.72	2.37	0.75	58.65	0.82	27.78	n.d.	99.83
6	2.13	7.01	3.06	0.83	58.40	0.56	26.69	0.96	99.63
7	2.79	6.39	2.22	0.86	59.23	0.79	27.82	n.d.	100.1
8	6.04	6.39	2.62	0.74	59.30	0.76	24.49	n.d.	100.3
9	2.65	3.23	1.45	0.94	63.96	1.43	23.38	2.97	100.0
10	1.99	6.72	3.42	0.75	59.08	0.62	27.69	n.d.	100.3
11	4.49	5.03	1.50	0.74	62.16	0.77	24.72	0.56	99.98
12	3.24	7.24	2.12	0.71	60.27	0.82	23.98	0.92	99.31

EC grain >63 µm

Grain	MgO	Al_2O_3	TiO_2	V_2O_3	Cr_2O_3	MnO	FeO	ZnO	Total

1*	4.37	6.25	2.40	0.75	56.79	0.72	26.48	n.d.	99.14

* Grain also contains 1.39 wt% NiO

OtC-V grains 32-63 μm

Grain	MgO	Al_2O_3	TiO_2	V_2O_3	Cr_2O_3	MnO	FeO	ZnO	Total
1	5.76	6.24	0.85	0.61	62.57	0.91	22.76	n.d.	99.71
2	5.79	11.14	0.80	0.56	58.03	0.54	23.95	n.d.	100.8
3	2.12	5.83	0.89	0.78	62.47	0.87	27.24	0.58	100.8
4	7.91	7.73	1.00	0.74	60.66	0.75	20.13	n.d.	98.92
5	1.75	n.d.	0.56	0.83	68.10	0.93	22.42	5.74	100.3
6	5.45	12.44	0.91	0.66	55.70	n.d.	25.46	n.d.	100.6
7	5.91	3.16	1.02	0.62	64.16	0.60	24.40	n.d.	99.87

OtC-V grains >63 μm

Grain	MgO	Al_2O_3	TiO_2	V_2O_3	Cr_2O_3	MnO	FeO	ZnO	Total
1	3.58	8.78	0.88	0.52	59.28	n.d.	26.72	n.d.	99.76
2	10.13	17.84	0.95	0.51	50.93	0.56	19.04	n.d.	99.96

OtC grains 32-63 μm

Grain	MgO	Al_2O_3	TiO_2	V_2O_3	Cr_2O_3	MnO	FeO	ZnO	Total
1	10.69	16.09	0.89	n.d.	42.23	n.d.	29.44	n.d.	99.34
2	7.65	20.21	n.d.	n.d.	51.08	0.93	18.17	1.79	99.83
3	7.94	21.57	n.d.	n.d.	53.41	n.d.	16.27	n.d.	99.19
4	12.86	30.31	0.41	n.d.	34.03	n.d.	22.63	n.d.	100.2
5	10.81	21.09	n.d.	0.39	47.07	n.d.	19.92	n.d.	99.27
6	8.15	22.28	0.68	0.26	46.22	n.d.	22.38	n.d.	99.97

Sample G6

3.95-4.05 m below base of Ly1, sample weight: 13.0 kg.

EC grains 32-63 μm

Grain	MgO	Al_2O_3	TiO_2	V_2O_3	Cr_2O_3	MnO	FeO	ZnO	Total
1	1.97	6.12	2.61	0.76	58.92	0.77	29.71	0.35	101.2
2	2.83	5.00	1.64	0.74	62.64	1.48	25.42	1.31	101.1

OtC-V grains 32-63 μm

Grain	MgO	Al_2O_3	TiO_2	V_2O_3	Cr_2O_3	MnO	FeO	ZnO	Total
1	1.94	12.27	2.84	0.61	45.03	0.38	34.60	0.51	98.18
2	4.87	12.69	0.92	0.70	52.91	n.d.	27.89	n.d.	99.97
3	7.67	11.09	n.d.	0.49	59.00	2.21	13.78	6.36	100.6
4	9.15	7.00	2.21	0.71	60.24	0.50	21.32	n.d.	101.1

OtC grains 32-63 µm

Grain	MgO	Al_2O_3	TiO_2	V_2O_3	Cr_2O_3	MnO	FeO	ZnO	Total
1	12.02	20.91	n.d.	n.d.	51.71	n.d.	15.12	n.d.	99.76
2	10.79	11.90	3.11	n.d.	33.21	n.d.	41.29	n.d.	100.3

Sample G7

Ca. 4.75 m below base of Ly1 (2-15 cm above "Trypanites Bed"), sample weight: 13.0 kg.

EC grains 32-63 µm

Grain	MgO	Al_2O_3	TiO_2	V_2O_3	Cr_2O_3	MnO	FeO	ZnO	Total
1	1.88	6.27	3.73	0.81	56.53	0.76	30.37	0.47	100.8
2	3.00	6.35	2.76	0.88	59.46	0.89	27.71	0.37	101.4
3	2.37	5.22	3.79	0.81	58.22	0.76	29.45	n.d.	100.6
4	3.09	6.12	2.20	0.70	60.55	0.89	27.18	n.d.	100.7
5	2.23	5.70	4.39	0.82	57.94	0.63	29.45	n.d.	101.2
6	2.44	6.23	3.90	0.69	57.65	0.73	28.95	n.d.	100.6
7	2.43	6.53	3.80	0.69	58.41	0.63	27.96	n.d.	100.5
8	1.93	6.53	3.01	0.74	57.30	0.73	29.53	0.57	100.3
9	3.25	5.98	2.52	0.68	59.77	1.18	26.56	0.28	100.2
10	2.59	6.75	1.60	0.76	59.73	1.32	27.07	0.47	100.3
11	1.98	5.77	2.14	0.69	59.89	1.06	28.14	n.d.	99.66

EC grains >63 µm

Grain	MgO	Al_2O_3	TiO_2	V_2O_3	Cr_2O_3	MnO	FeO	ZnO	Total
1	5.57	5.57	3.86	0.86	58.76	n.d.	26.27	n.d.	100.9
2	6.24	6.79	1.21	0.63	62.85	0.73	22.36	n.d.	100.8

OtC-V grains 32-63 µm

Grain	MgO	Al_2O_3	TiO_2	V_2O_3	Cr_2O_3	MnO	FeO	ZnO	Total
1	2.01	0.75	1.46	0.59	41.33	0.81	52.16	1.17	100.3
2	4.07	14.86	1.54	0.61	51.22	n.d.	27.60	n.d.	99.90

OtC grains 32-63 µm

Grain	MgO	Al_2O_3	TiO_2	V_2O_3	Cr_2O_3	MnO	FeO	ZnO	Total
1	8.56	16.91	n.d.	0.45	51.08	n.d.	22.80	0.43	100.2
2	8.67	11.77	1.18	n.d.	49.34	n.d.	28.95	n.d.	99.90
3	11.35	9.76	0.37	n.d.	57.29	n.d.	21.50	n.d.	100.3
4	12.03	10.73	0.39	n.d.	56.55	0.46	19.29	n.d.	99.45
5	7.31	10.18	n.d.	n.d.	69.14	n.d.	12.73	1.32	100.7

6	5.59	20.98	n.d.	n.d.	42.40	0.43	30.37	0.41	100.2
7	8.02	9.11	1.07	n.d.	48.43	n.d.	33.57	n.d.	100.2
8	8.03	18.14	n.d.	n.d.	56.33	0.49	16.73	n.d.	99.73
9	11.14	28.74	n.d.	n.d.	45.72	n.d.	14.44	n.d.	100.0
10	7.49	12.57	n.d.	n.d.	60.63	n.d.	19.45	n.d.	100.2
11	12.15	19.06	0.97	n.d.	42.37	n.d.	25.30	n.d.	99.85
12	4.36	15.64	1.38	0.43	44.90	n.d.	33.18	0.40	100.3
13	10.89	15.77	1.06	0.34	45.85	n.d.	25.60	n.d.	99.51
